# Sabatier‐Adjusted *d*‐Band Centers of Scalable Asymmetric Iron Sites toward Dynamic Nonradical Network for Fast Mineralization with Low‐Amount Oxidant

**DOI:** 10.1002/advs.202515593

**Published:** 2025-11-25

**Authors:** Yue Chen, Xunheng Jiang, Zhiyu Pan, Zhenjie Li, Chaohuang Chen, Can Li, Chenghui Luo, Shengkun Zhang, Daohui Lin, Xinhua Xu, Jiang Xu

**Affiliations:** ^1^ State Key Laboratory of Soil Pollution Control and Safety Zhejiang University Hangzhou 310058 China; ^2^ College of Environmental and Resource Sciences Zhejiang University Hangzhou 310058 China; ^3^ Zhejiang Provincial Key Laboratory of Organic Pollution Process and Control Zhejiang University Hangzhou 310058 China

**Keywords:** asymmetrical coordination structures, environmental nanotechnology, Fe single‐atom catalyst, nonradical network, *p*‐block element

## Abstract

Nonradicals relying on peroxymonosulfate (PMS) activation face inherent kinetic and thermodynamic limitations in pollutant mineralization. This is overcome using asymmetric iron single‐atom catalysts (Fe‐SACs) with Sabatier‐adjusted *d*‐band centers that establish a synergistic nonradical network of pathways, achieving exceptional mineralization (≈85%) with only one‐tenth conventional PMS dosage. Direct coordination of high‐loading Fe (≈10 wt.%) with controllable *p*‐block elements (S, P, B) simultaneously facilitates electron‐transfer pathways and selective generation of nonradicals (^1^O_2_, Fe^IV ^= O). Compared to symmetric Fe_1_‐N_4_, they enhance pollutant removal and mineralization by 4.2‐fold and 6.3‐fold, respectively. Atomic‐resolution characterization and theory reveal that the dopant's electronegativity governs the electronic perturbations of the Fe center, dictating PMS adsorption. Pollutant‐specific charge transfer and adsorption affinity critically determine nonradical activity. The asymmetric Fe SACs exhibit excellent stability and applicability in complex matrices and continuous‐flow wastewater treatment. This work provides a transformative strategy to overcome fundamental limitations in advanced oxidation processes and a design framework for sustainable environmental technologies.

## Graphical abstract

Asymmetric iron single‐atom catalysts with Sabatier‐adjusted *d*‐band centers overcome the inherent limitations of peroxymonosulfate (PMS) activation by establishing a synergistic nonradical pathway network, achieving exceptional pollutant mineralization (≈85%) at only one‐tenth conventional PMS dosage. The stability and scalability offer a transformative solution for sustainable wastewater treatment.

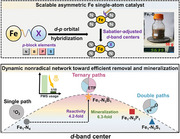

## 1 Introduction

Various traditional and emerging contaminants with bio‐recalcitrance in wastewater and groundwater have brought global challenges to environmental health and sustainable water supply.^[^ ^1^, ^2^, ^3^ ^]^ Nonradicals derived from peroxymonosulfate (PMS) activations possess high selectivity, long lifetimes, and broad pH adaptability, holding a compelling promise for water treatments.^[^ ^4^, ^5^, ^6^ ^]^ Although organic contaminants can be efficiently removed by nonradicals over radical scavengers (e.g., natural organic matter and anions) due to their strong resistance to complex water matrices, their mineralization efficiencies are often low.^[^ ^7^ ^]^

The versatile activities of nonradicals provide a potential strategy for breaking the balance between removal and mineralization efficiencies. Generally, singlet oxygen (^1^O_2_) with an unoccupied π* orbital can selectively remove electron‐rich contaminants, while its low redox potential (1.88 V) and lifetime (≈2–4 µs) lead to insufficient oxidation. High‐valent iron‐oxo species (Fe^IV ^= O, *E *= 2.2 V, pH = 3) with longer lifetimes (≈7 s) and steady‐state concentrations (10^−8^ m) provide sustained oxidative capacity at the cost of sluggish kinetics.^[^ ^8^, ^9^ ^]^ In contrast, the electron transfer pathway (ETP) revolutionizes PMS utilization by directly extracting electrons from pollutants, bypassing short‐lived ROS generation.^[^ ^10^ ^]^ ETP enables rapid oxidation initiation but fails to maintain deep degradations due to the transient reactive intermediates and absence of active species, which promotes the polymerization of electron‐depleted contaminants that subsequently block the catalytic sites.^[^ ^11^ ^]^ This kinetic‐thermodynamic trade‐off stems from the inability of a single nonradical pathway to concurrently balance activation energy barriers and oxidative longevity.^[^ ^12^ ^]^ A tandem reaction system integrating low‐barrier electron transfer with sustained ^1^O_2_ and Fe^IV ^= O is expected to overcome this bottleneck by establishing a dynamic degradation network, where sequential oxidation steps synergistically mineralize recalcitrant intermediates.^[^ ^13^ ^]^

The simple structural model and well‐defined active sites of iron single‐atom catalysts (Fe SACs) hold promise for a paradigm shift in the design of the catalyst's microenvironment, offering unparalleled precision in tailoring PMS activation toward efficient and selective generation of nonradicals.^[^ ^14^, ^15^, ^16^, ^17^, ^18^ ^]^ However, symmetrical Fe–N_4_ coordination inherently constrains charge density distribution, limiting the tunability of PMS adsorption modes and forcing suboptimal activation pathways.^[^ ^19^, ^20^ ^]^ Recent efforts to construct asymmetrical coordination structures, such as introducing heteroatoms or defects, have shown promise in breaking the symmetry of the metal center's charge density, thereby enabling multi‐pathway activations.^[^ ^21^, ^22^, ^23^ ^]^ The disparate electronegativities of non‐metal *p*‐block elements exemplified by boron (*χ* = 2.04), phosphorus (*χ* = 2.19), and sulfur (*χ* = 2.58) relative to nitrogen (*χ* = 3.04) induce electronic heterogeneity at the iron metal center when these *p*‐block elements are coordinated.^[^ ^24^, ^25^, ^26^, ^27^ ^]^ Furthermore, the pronounced electronegativity contrast between *d*‐block Fe and nonmetallic *p*‐block elements drives *p–d* orbital coupling, generating hybridized orbitals that effectively engage in the dynamic adsorption of PMS or pollutant molecules.^[^ ^28^, ^29^ ^]^ However, the systematic frameworks to link coordination asymmetries of Fe sites with the selective generation of nonradicals remain unclear, or whether deep mineralization of contaminants could be achieved by tandem reaction of multiple nonradicals, even at relatively low PMS dosages.^[^ ^30^ ^]^

Central to such synergy of multiple nonradicals is the precise modulation of PMS adsorption configurations at catalytic sites, guided by the Sabatier principle of the volcano for PMS adsorption—a thermodynamic sweet spot that balances adsorption strength and intermediate desorption.^[^ ^31^ ^]^ Achieving this balance, however, demands atomic‐level control over catalyst electronic structures to orchestrate multi‐pathway activation.^[^ ^32^ ^]^ The *d*‐band theory explains the reactivity of transition metals by linking it to the position of the *d*‐band center, which is influenced by their unfilled *d* orbitals.^[^ ^33^ ^]^ The high electronegativity and diverse bonding configurations of *p*‐block dopants disrupt charge symmetry at Fe sites, shifting *d*‐band centers to weaken metal–PMS bonds (facilitating electron transfer) while positioning PMS adsorption near the Sabatier optimum (enabling ^1^O_2_ or Fe^IV ^= O generation).^[^ ^34^, ^35^ ^]^ However, the correlations between the partially filled *p* orbital and tunable reactivity are still an open question, leaving the design of dynamic degradation networks largely empirical.

We chose a typical 2D layered material, carbon nitride (CN), as an ideal carrier for single‐atom catalysts. To address these existing gaps, this work engineers asymmetrical Fe‐N_3_X_1_ centers (X denoted as N, S, P, and B), demonstrating how electronegativity gradients and *d*‐band modulation orchestrate tandem nonradical pathways. Precisely constructing asymmetrical high‐loading Fe sites (≈10 wt.%) with *p*‐block element coordination enabled the controlled synthesis of nonradicals (e.g., single, double, or ternary). This dynamic degradation network enables rapid degradation of organic contaminants with deep mineralization at low PMS dosage, exhibiting universal activity across various contaminants and water matrices. This study opens an avenue for advancing water treatments by decoding the interplay between the coordination asymmetry of SACs and the dynamic degradation network of nonradicals.

## 2 Results and Discussion

### 2.1 Constructions of Asymmetric High‐Loading Fe SACs with *d–p* Orbital Hybridization

**Figure 1:**
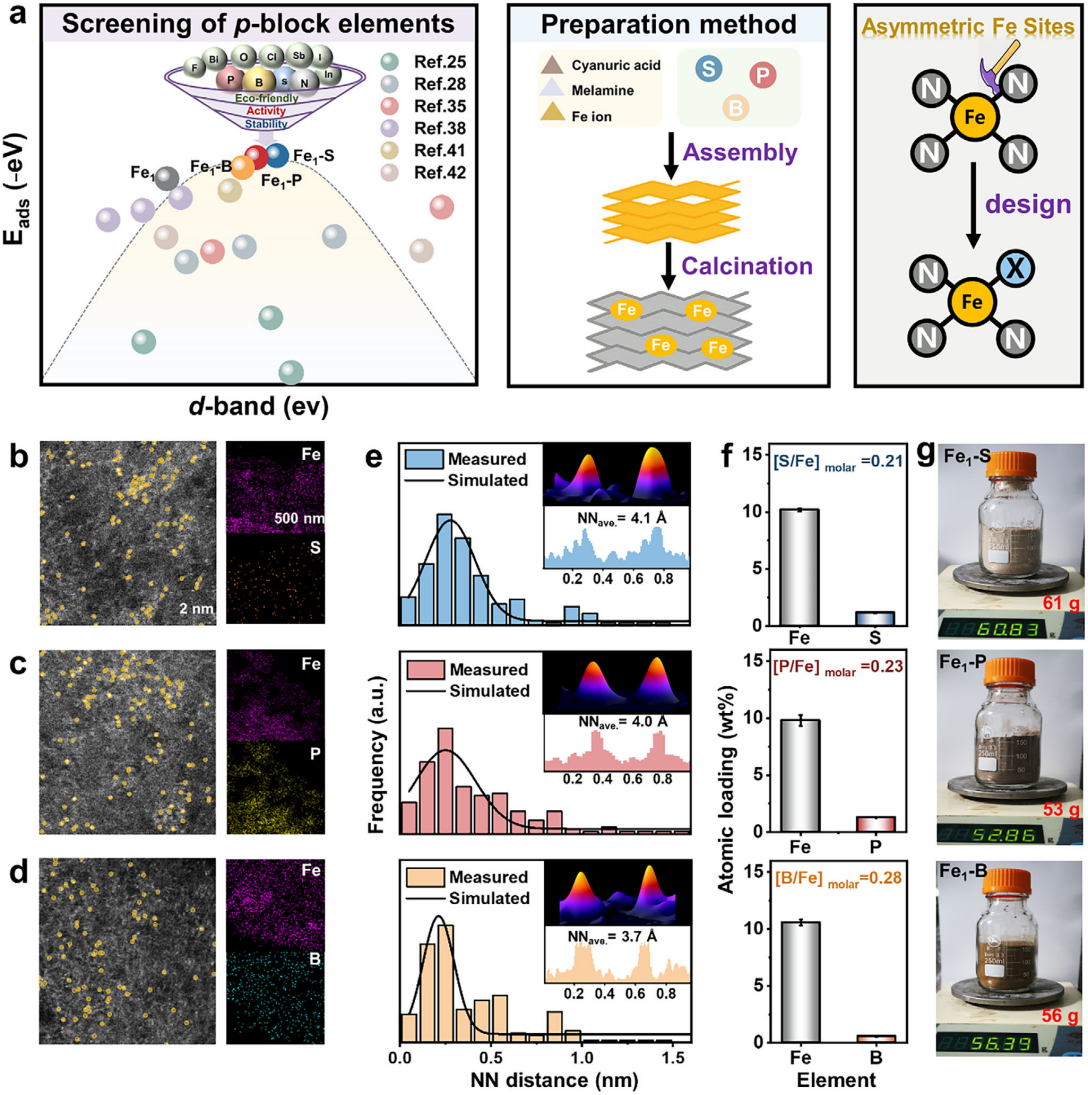
Characteristics of asymmetric Fe SACs. a) Reasonable selection and design of *p*‐block element coordinated Fe SACs. AC‐HAADF‐STEM images and EDS images of b) Fe_1_‐S, c) Fe_1_‐P, and d) Fe_1_‐B. e) Histogram and fitting of distance distribution between neighboring Fe atoms (inset represents the 3D intensity and line profile of neighboring Fe atoms). f) Atomic loading of single‐atomic Fe and *p*‐block elements in asymmetric catalysts. g) The digital image of 50 g Fe_1_‐S, Fe_1_‐P, and Fe_1_‐B.

The *d*‐band center of transition metal catalysts regulates PMS adsorption and activation, thereby governing the generation of reactive species and pollutant degradation. Using *PMS intermediate binding energy as a descriptor, we identified the Fe‐N_3_X_1_ (where *X* = B, P, S, N) configuration as a potential high‐activity site for PMS activation. This data‐driven screening strategy proactively predicts structure–activity relationships between catalysts and *PMS descriptors, accelerating the rational design of advanced oxidation catalysts (Table S6, Supporting Information). The synthesis was based on an acid‐modulated supramolecular self‐assembly approach, where dopant acids protonated nitrogen‐rich precursors, facilitating simultaneous coordination of Fe^2+^ with heteroatoms during self‐assembly (Figure S1, Supporting Information).^[^ ^36^ ^]^ The local Fe coordination was designed to be transformed from a symmetric Fe_1_‐N_4_ environment to an asymmetric Fe_1_‐N_3_X_1_ (*X* = S, P, or B) structure via a *d–p* orbital hybridization strategy (**Figure** **1**a), that is, between the Fe 3*d* orbitals and the valence *p* orbitals (2*p* for B; 3*p* for P and S). Aberration‐corrected high‐angle annular dark‐field scanning transmission electron microscopy (AC‐HAADF‐STEM) visualized isolated bright spots, indicative of atomically dispersed Fe atoms (Figure 1b–d; Figures S2,S3, Supporting Information), consistent with the absence of diffraction peaks corresponding to crystalline iron species in the X‐ray diffraction (XRD) patterns (Figure S4, Supporting Information). Energy‐dispersive X‐ray spectroscopy (EDS) elemental mapping further revealed the homogeneous distribution of C, N, Fe, and the respective *p*block elements (S, P, or B) throughout the support.

The average nearest‐neighbor (NN) Fe distances (derived from >100 Fe atoms) for Fe_1_‐S (0.41 nm), Fe_1_‐P (0.40 nm), and Fe_1_‐B (0.37 nm) were close to Fe_1_ (0.40 nm) (Figure 1e; Figure S3f, Supporting Information), indicating negligible impacts of the asymmetric coordination on Fe dispersion. These distances were substantially greater than the Fe–Fe metallic bond length (0.207 nm), and the electron delocalization between Fe *d*‐orbitals and dopant *p*‐orbitals would effectively enhance metal–support interactions and stabilize atomically dispersed Fe species. Notably, this precisely controlled heteroatom‐doped strategy preserved the fundamental architecture of the CN framework, as evidenced by the intact tri‐s‐triazine units observed in Fourier transform infrared spectroscopy (FTIR) (Figure S5, Supporting Information) and retention of the characteristic crystal plane (002) with interlayer *π–π* stacking in CN observed via XRD patterns.^[^ ^37^ ^]^ Inductively coupled plasma optical emission spectroscopy (ICP‐OES) suggests that the asymmetric coordination with *p*‐block elements did not significantly change the Fe loading (i.e., maintaining ≈10 wt.% across all catalysts) (Table S1, Supporting Information), and *p*‐block/Fe atomic ratios ranged from 0.21–0.28 (Figure 1f). Consequently, this facile coordination between the inherent properties of the *d*‐block and *p*‐block elements provided a facile mass‐production (53–61 g) for asymmetric Fe sites (Figure 1g) with high Fe loading (≈10 wt.%), well exceeding the loading of other commercial and scalable single‐atom catalysts (typically less than 2 wt.%). These comprehensive structural characterizations confirmed both the atomic dispersion of Fe and the overall structural integrity of the catalysts.

### 2.2 Local Structure and Properties of First‐Shell Coordinated Asymmetric Fe SACs

**Figure 2:**
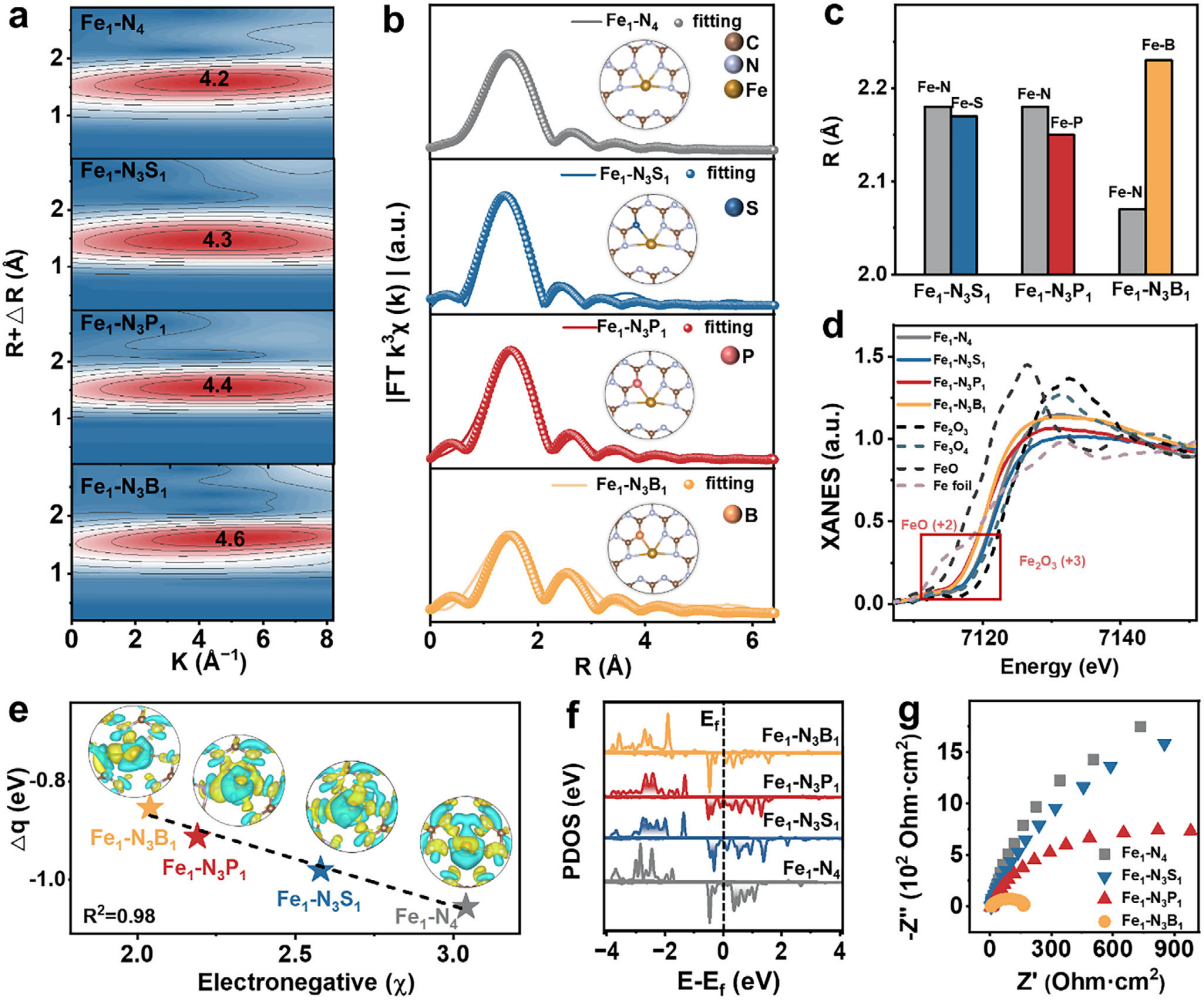
Atomic local structure and physicochemical properties of symmetric and asymmetric Fe SACs. a) WT‐EXAFS profiles at Fe K‐edge and b) EXAFS fitting curves (solid lines: experimental data; dots: fitted results) with coordination structure schematics (inset). c) Variation of bond length after asymmetric coordination. d) Normalized XANES spectra showing edge energy shifts versus reference materials. e) Correlation between charge density change (Δ*q*) at Fe sites and dopant electronegativity (*χ*) with charge redistribution maps (inset: blue/yellow = electron depletion/accumulation). f) PDOS comparison highlighting the *d*‐band center (dashed lines) shifting induced by asymmetric coordination. g) EIS of electrodes made from catalysts.

The local structure and coordination environment of asymmetric Fe SACs were analyzed by Fe K‐edge extended X‐ray absorption fine structure (EXAFS) spectra using both Wavelet Transform (WT) and Fourier Transform (FT) methods. The absence of Fe‐Fe scattering paths in WT‐EXAFS and metallic Fe─Fe bond peak in FT‐EXAFS (**Figure** **2**a; Figures S6,S7, Supporting Information) further established the exclusive presence of isolated Fe sites, corroborating HAADF‐STEM observations (Figure 1b–d). Concurrently, the asymmetric coordination of S, P, and B shifted the WT intensity of characteristic Fe‐N coordination (*k* = 4.1 Å^−1^) to 4.3, 4.4, and 4.6 Å^−1^, respectively. Distinct FT‐EXAFS features in the first coordination shell (1.5–2 Å) varied systematically with the dopant identity, demonstrating successful local Fe coordination tuning via targeted heteroatom integration (Figure 2b). Quantitative EXAFS fitting revealed distorted and asymmetric coordination geometries Fe‐N_3_X_1_ (*X* = S, P, or B), indicating distinct alterations in the Fe‐centric bond distances. B dopant induced elongated Fe─B bonds, while S and P dopants engendered shorter Fe─S and Fe─P bonds, respectively (Figure 2c, Table S2, Supporting Information).

This structural asymmetry was hypothesized to enhance catalytic activity by inducing local charge polarization and optimizing orbital interactions, thereby potentially lowering activation barriers for key reaction intermediates. The Fe K‐edge X‐ray absorption near‐edge structure (XANES) profiles of the catalysts, with Fe^2+^ (FeO) and Fe^3+^ (Fe_2_O_3_) as reference standards. The energy shift of the absorption edge indicates that the average Fe oxidation state in all materials ranges between +2 and +3 (Figure 2d), following the order of Fe_1_‐N_4_ > Fe_1_‐N_3_S_1_ > Fe_1_‐N_3_P_1_ > Fe_1_‐N_3_B_1_. This trend was consistent with Fe 2*p* XPS results (Figure S8, Supporting Information), which were further substantiated by theoretical calculations. Quantitative analysis revealed a clear inverse correlation between the calculated charge density change (Δ*q*) at the Fe centers and the electronegativity (*χ*) of the coordinated ligands (Figure 2e; Figure S9, Table S3, Supporting Information). Associated electron density difference plots (blue: depletion; yellow: accumulation) suggest distinct electronic interactions between the Fe center and coordinated *p*‐block elements. These modifications were governed by the trend in dopants’ electronegativity (*χ*): N (3.04) > S (2.58) > P (2.19) > B (2.04). The high electronegativity in the reference Fe_1_‐N_4_ structure significantly withdrew electron density from Fe, stabilizing a higher effective oxidation state. Conversely, the low electronegativity of S, P, and B promoted net electron donation to the Fe center (significant yellow accumulation), thereby lowering its effective oxidation state. Additionally, this modulation of the local electronic structure directly changed the energy of the *d*‐band center relative to the Fermi level (Figure S10, Supporting Information). Generally, the Fermi level is a critical descriptor influencing the adsorption energetics of reactants and intermediates, and a lower electron density at the Fe site leads to a downshift of its *d*‐band center relative to E_f_.^[^ ^38^ ^]^ For instance, the Fe_1_‐N_3_B_1_ catalyst exhibits a relatively lower *d*‐band center compared to Fe_1_‐N_4_, which predicts weakened adsorption strength toward reaction species (Figure 2f).^[^ ^39^ ^]^ Such moderated adsorption is often advantageous for catalytic cycles dependent on rapid adsorption/desorption steps. Electrochemical impedance spectroscopy (EIS) revealed that the asymmetric coordination of Fe with S, P, or B could lower the charge‐transfer resistance (Figure 2g), suggesting enhanced interfacial electron transfer capabilities during the reaction. This optimization was expected to contribute to improved catalytic efficiency and alter the reaction selectivity. Detailed experimental results substantiating these structure–property relationships are presented in subsequent sections.

### 2.3 Dynamic Nonradical Network Induced by p‐Block Element Coordinated Asymmetric Sites

**Figure 3:**
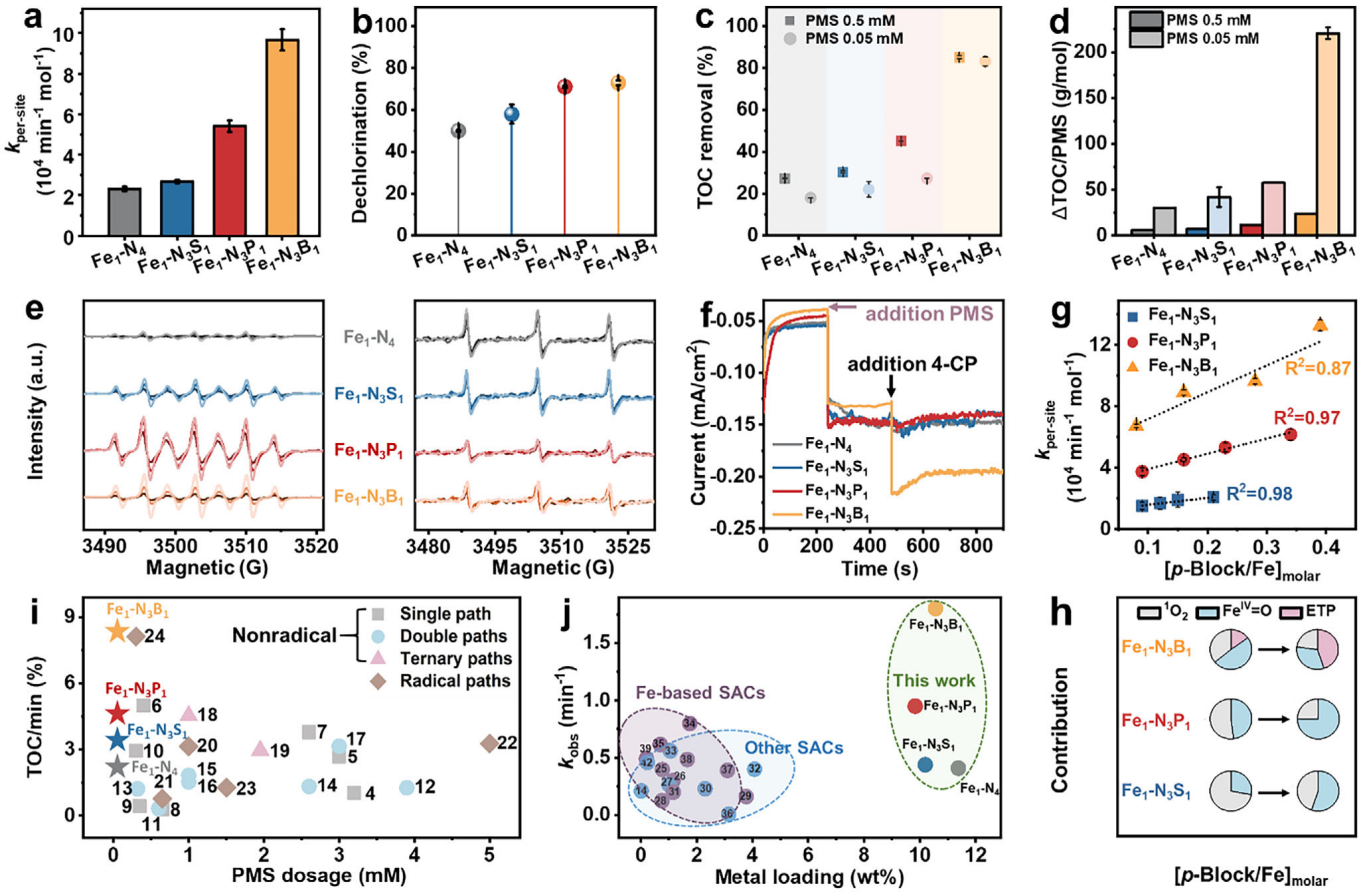
Dynamic nonradical network induced by *p*‐block element coordinated asymmetric sites for enhanced Fenton‐like performance and tunable active species generation. a) degradation kinetics and b) dechlorination efficiency of 4‐CP by asymmetric Fe SACs at a conventional dosage of PMS. Comparison of c) overall TOC removal efficiency d) TOC removal per mole of PMS at conventional and low (1/10) PMS dosages. EPR spectra identifying e) TEMP‐^1^O_2_ and DMPOX adducts. f) *i*–*t* curves with sequential PMS and 4‐CP additions. g) Correlations of degradation kinetics with doping ratios and h) tunable contributions of nonradical pathways to 4‐CP degradation, the ETP contribution was indirectly assessed by subtracting the quantified contributions from other reactive species. Comparison of i) mineralization rate and PMS dosage and j) reaction rate and metal loading of asymmetric Fe SACs/PMS with literature‐reported systems.

The enhanced performance of Fe SACs by asymmetric coordination was examined with a commonly used reactivity probe, that is, 4‐chlorophenol (4‐CP). The Fe content normalized degradation rate (*k*_per‐site_) of Fe_1_‐N_3_S_1_, Fe_1_‐N_3_P_1_, and Fe_1_‐N_3_B_1_ was 1.2, 2.3, and 4.2 times higher than that of the pristine Fe_1_‐N_4_ (**Figure** **3**a; Figure S11, Supporting Information), consistent with their orders of electronic structures and properties (Figure 2). Meanwhile, the dechlorination efficiency of 4‐CP was also enhanced accordingly (Figure 3b), in agreement with the nonradical degradation pathway that targets electron‐rich groups (e.g., ─OH), as discussed below.^[^ ^40^, ^41^ ^]^ While the selective generation of ^1^O_2_ by symmetrical Fe_1_‐N_4_ sites and its role in 4‐CP degradation with limited mineralization had been well established by us and other groups, the asymmetric coordination with S, P, and B can improve pollutant mineralization efficiency to different degrees. This indicates that asymmetric coordination of Fe preferentially steers the PMS activation toward generating multiple reactive species with mineralization capability. Notably, the highest values of *k*_per‐site_, dechlorination efficiency, and total organic carbon (TOC) removal for Fe_1_‐N_3_B_1_ indicate the significance of mineralization rather than solely accelerating the initial dechlorination step. Moreover, this asymmetric Fe_1_‐N_3_B_1_ coordination maintained comparable TOC removal (≈85%) even with ultralow PMS concentration (1/10 of the initial dosage, i.e., 0.05 mm) (Figure 3c). Furthermore, in comparison to other configurations, Fe_1_‐N_3_B_1_ achieved a significant enhancement in pollutant mineralization per mole of PMS consumed (Figure 3d). This suggests the relatively high PMS utilization by Fe_1_‐N_3_B_1_ efficiency and its versatile activation pathways. The detailed dynamic nonradical network of asymmetric Fe SACs is discussed below.

Reactive species generated during PMS activation by Fe SACs were identified by electron paramagnetic resonance (EPR) spectroscopy using trapping agents. Only 2,2,6,6‐tetramethylproline (TEMP)‐^1^O_2_ triple signals were detected for the Fe_1_‐N_4_ catalyst, suggesting a ≈100% selective generation of ^1^O_2_. The asymmetric coordination with S maintained the primary generation of Fe^IV ^= O with a heptad signal of 5,5‐dimethylpyrrolidone‐2‐(oxy) (DMPOX) adducts, which were attributed to Fe^IV ^= O‐mediated oxidation (Figure 3e; Figure S12, Supporting Information). In contrast, the Fe_1_‐N_3_P_1_ exhibited the most intense DMPOX signals, with an increasing trend during the PMS activation observed in the Fe_1_‐N_3_P_1_ system. Notably, both TEMP‐^1^O_2_ and DMPOX signals displayed a significant increasing trend over the reaction period for the Fe_1_‐N_3_B_1_ system. The absence of EPR signals for radicals and their limited contributions to 4‐CP degradation in quenching tests (Figure S23, Supporting Information) confirmed that the asymmetrically coordinated Fe SACs could selectively generate nonradicals. The generated nonradicals and consumed PMS were quantified to assess the PMS utilization efficiency (Figures S13–S15, Supporting Information). Consistent with EPR observations, the Fe_1_‐N_3_B_1_ system demonstrated increasing ^1^O_2_ and Fe^IV ^= O generation efficiency throughout the reaction, suggesting sustained generation or an increasingly significant role in later stages. Intriguingly, despite its superior pollutant degradation performance, Fe_1_‐N_3_B_1_ exhibited the lowest overall combined ^1^O_2_ and Fe^IV ^= O yield (Figure S16, Supporting Information). This discrepancy strongly suggests the alternative activation pathways for the Fe_1_‐N_3_B_1_ system as discussed below.^[^ ^42^ ^]^

Distinct chronoamperometric (*i–t*) current transients in the Fe_1_‐N_3_B_1_ system (upon sequential PMS and 4‐CP addition) (Figure 3f) provided the first proof of enhanced electron transfer from 4‐CP to PMS. This was likely mediated by reactive surface species (Figure S17, Supporting Information), offering direct electrochemical evidence for ETP.^[^ ^13^ ^]^ Besides, a hallmark of ETP was that the PMS consumption by Fe_1_‐N_3_B_1_ uniquely exhibited substrate‐dependent activation, with its consumption increasing ≈40% upon the 4‐CP addition. In contrast, other catalysts remained relatively stable in PMS consumption (Figure S18, Supporting Information).^[^ ^43^ ^]^ These results confirmed that the symmetric Fe_1_‐N_4_ coordination provided a single nonradical path (^1^O_2_), the asymmetric coordination of S and P upgraded to a double nonradical path (^1^O_2_ and Fe^IV ^= O), and asymmetrically coordinating Fe with B further promoted to a ternary nonradical path (^1^O_2_, Fe^IV ^= O, and ETP). Fe_1_‐N_3_B_1_ could act as an electron shuttle for the rapid initiation of contaminant degradation, while ^1^O_2_ and Fe^IV ^= O subsequently ensured sustained degradation, offering a high degradation rate and mineralization efficiency (Figure 3a,b).

Systematic variation of the initial dopant‐to‐iron molar ratio ([X/Fe]) showed a strong positive correlation with the *k*_per‐site_ (*R* ^2^ = 0.81–0.99) (Figure 3g; Figure S19, Supporting Information). Quenching experiments subsequently dissected the contributions of distinct nonradical pathways (^1^O_2_, Fe^IV ^= O, ETP) to the overall degradation, demonstrating that altering the doping ratio significantly shifted relative pathway contributions (Figure 3h; Figures S20–S23, Supporting Information). Intriguingly, maximizing a specific element's doping level did not invariably yield performance superior to the optimal dopant type. This suggests that while dopant loading could modulate catalytic activity, the fundamental chemical properties of the dopant element played a predominant role in determining both the ultimate catalytic efficiency and the prevailing reaction mechanism, as elucidated below. Comparative analysis further underscores the superior mineralization performance of asymmetric Fe SACs against literature‐reported catalysts with nonradical pathways, even at the lowest dosage of PMS (Figure 3i, Table S4, Supporting Information). Furthermore, the asymmetric Fe SACs in this work also outcompete most state‐of‐the‐art Fe SACs and other SACs in both degradation rate and metal loading (Figure 3j, Table S5, Supporting Information). Collectively, these comparisons establish highly promising and sustainable catalysts (especially Fe_1_‐N_3_B_1_) for advanced oxidation processes.

### 2.4 Sabatier‐Adjusted *d*‐Band Centers of Iron Sites toward Optimized Nonradical Generation

**Figure 4:**
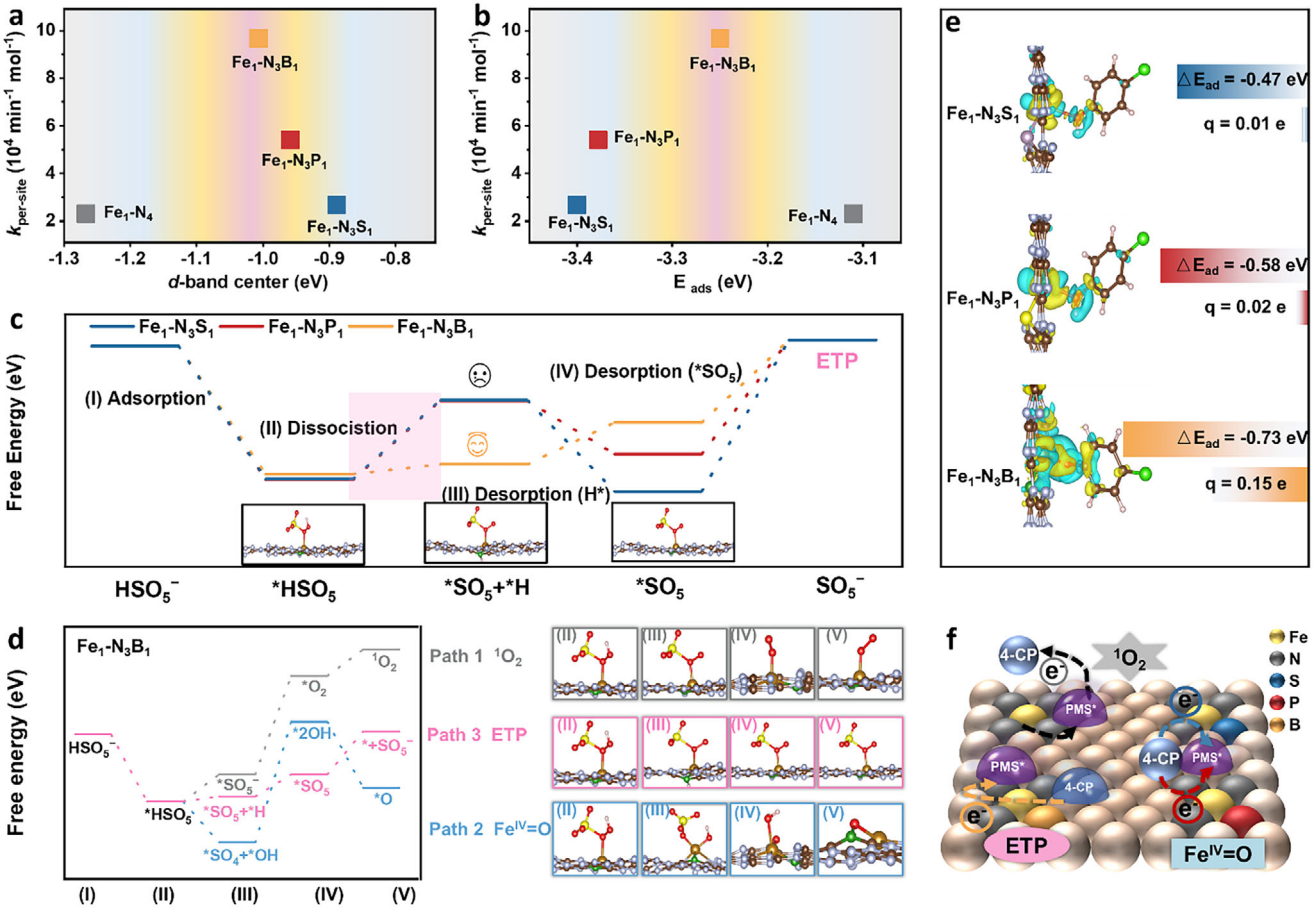
Mechanism analysis of asymmetric coordination‐driven selective generation of reactive species. Theoretical activity volcano plots of correlating a) *d*‐band center and b) PMS adsorption energy with *k*_per‐site_. c) Comparison of energy barrier diagrams for the ETP of different asymmetric Fe SACs. d) Energy barrier profiles and structure evolution for the generation of ^1^O_2_, Fe^IV ^= O, and ETP on the Fe_1_‐N_3_B_1_. e) 4‐CP adsorption energy on asymmetric Fe SACs with Bader charge analysis (blue: electron accumulation, yellow: electron depletion). f) Schematic reaction mechanisms for ^1^O_2_, Fe^IV ^= O, and electron transfer pathways.

Distinctive *d*‐band center modulation effects of asymmetric coordination on Fe SACs activity were revealed via integrating theoretical calculations with kinetics. The interfacial interaction analysis demonstrated volcano‐type structure–activity relationships governed by the *d*‐band center and PMS adsorption energy (*E*_ads_) (**Figure** **4**a,b), where the Fe_1_‐N_3_B_1_ was located at the volcano top. The heteroatom‐induced electronic reconstruction enabled self‐adaptive electronic coupling at active sites through *p‐d* orbital hybridization, strategically shifting Fe sites at the Sabatier volcano apex and optimizing the adsorption–desorption equilibrium of critical intermediates (e.g., *SO_5_, *H) (Figure 4c). Notably, the asymmetric coordination microenvironment created in the Fe_1_‐N_3_B_1_ system dynamically redistributed charges, enhancing PMS adsorption and reducing the *HSO_5_ dissociation energy as a significant activation barrier.^[^ ^44^ ^]^

Relative thermodynamic trends for ^1^O_2_, Fe^IV ^= O, and ETP generation pathways during PMS activation by Fe_1_‐N_3_B_1_ were further elucidated (Figure 4d), with accompanying structural models (right panel) depicting the evolution of PMS‐surface interactions and conformational changes. All pathways initiated with exothermic HSO_5_^−^ adsorption onto active sites (state I→II), indicating a favorable initial interaction. Subsequently, pathways diverged. The ^1^O_2_ pathway involved *SO_5_^−^ conversion to adsorbed *O_2_, followed by ^1^O_2_ desorption, and the Fe^IV ^= O pathway proceeded via *HSO_5_ decomposition to *SO_4_ and *OH, followed by the transformation to a surface‐bound *O representing Fe^IV ^= O. In contrast, the ETP pathway consistently presented the lowest free energy barriers for key steps (e.g., II→III) and the most accessible formation of crucial intermediates. This free energy analysis thereby compellingly suggests ETP as the dominant and energetically preferred reaction pathway, providing thermodynamic insights into the complex PMS activation network and underscoring the ETP's pivotal role.

Catalyst–pollutant interactions were investigated to delineate the electronic underpinnings of preferential ETP on asymmetric Fe SACs. Differential charge density analysis revealed pronounced electron redistribution upon 4‐CP adsorption onto Fe_1_‐N_3_B_1_, indicating stronger interfacial coupling compared to S and P coordinated versions (Figure 4e). Quantitative analysis corroborated this trend, demonstrating substantially larger charge transfer (Δ*q* = 0.15 e) from 4‐CP to Fe_1_‐N_3_B_1_ relative to Fe_1_‐N_3_S_1_ (0.01 e) and Fe_1_‐N_3_P_1_ (0.02 e). This significant charge transfer is identified as a critical determinant for initiating ETP, as it augments Fe_1_‐N_3_B_1_ capacity to extract electrons from adsorbed 4‐CP, thereby lowering the 4‐CP adsorption activation barrier and promoting the redox reaction. In aggregate, these findings comprehensively demonstrate that pollutant degradation facilitated by *p*‐block element coordination of Fe SACs under PMS activation is fundamentally dictated by intricate interfacial adsorption and subsequent electron transfer dynamics. Using the Fe_1_‐N_3_X_1_ catalyst system for 4‐CP degradation as a paradigm, three distinct mechanistic activation pathways are delineated (Figure 4f): (I) the ^1^O_2_ pathway, wherein PMS adsorption on the catalyst facilitated electron transfer to Fe metal center, yielding ^1^O_2_ that subsequently oxidized dissolved 4‐CP; (II) the Fe^IV ^= O pathway, where interaction of PMS with the Fe center generated Fe^IV ^= O species, which directly oxidized adsorbed 4‐CP via interfacial electron transfer; and (III) the ETP pathway, initiated by 4‐CP adsorption, which enabled electron shuttling from the pollutant to PMS, mediated by the Fe metal center. The synergistic interplay of these pathways established a multifaceted degradation network, underpinning sustained oxidative activity and enhanced process robustness.

### 2.5 Application Potential Assessments of Versatile Asymmetric Fe SACs for Water Treatments

**Figure 5:**
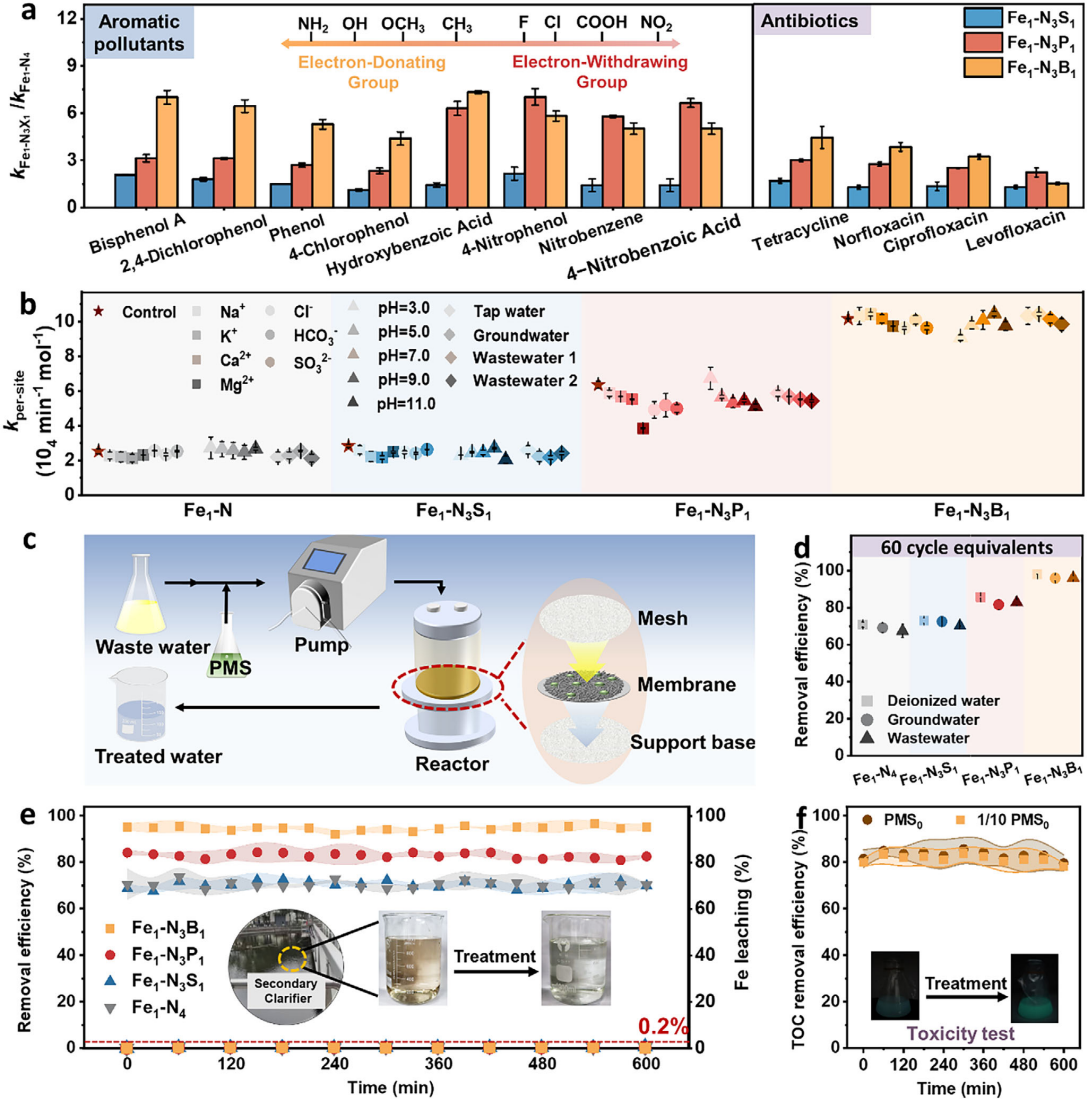
Application potential assessments of asymmetric Fe SACs for water treatments. a) Enhanced degradation of various organic contaminants with different electronic properties by asymmetric Fe SACs compared to Fe_1_‐N_4_. b) Performance of asymmetric Fe SACs under varied pH, coexisting ions, and water matrices. c) Schematic continuous‐flow device fabricated with catalysts. d) Average removal efficiencies of 4‐CP in continuous operation under different water matrices. e) Comparison of different asymmetric Fe SACs for deep treatment of 4‐CP in industrial wastewater and Fe leaching during continuous‐flow treatment. f) Stable mineralization performance during continuous‐flow treatment with comparison to the 1/10 dosage of PMS.

Various organic contaminants with different electronic properties were used to assess the universal performance of these asymmetric Fe SACs, including chlorophenols, antibiotics, and nitrobenzenes. All the asymmetric coordination enhanced the activity compared to Fe_1_‐N_4_ (**Figure** **5**a; Figures S24,S25, Supporting Information). Generally, Fe_1_‐N_3_P_1_ and Fe_1_‐N_3_B_1_ catalysts with double and ternary activation pathways show better universality than Fe_1_‐N_4_ and Fe_1_‐N_3_S_1_ catalysts. The Fe_1_‐N_3_P_1_ was superior for pollutants with electron‐withdrawing groups, whereas Fe_1_‐N_3_B_1_ excelled with pollutants with electron‐donating groups.^[^ ^45^ ^]^ The relatively high performance of Fe_1_‐N_3_B_1_ for pollutants with EDGs could be explained by the electrophilicity index (*ω*) (Figure S26, Table S7, Supporting Information). A higher *ω* value signifies a greater driving force for electron acceptance.^[^ ^46^ ^]^ The more rapid degradation of pollutants with lower *ω* values (e.g., electron‐rich 4‐CP and electron‐deficient HBA) indicates their greater propensity as electron donors to interact with the catalyst active sites, thereby facilitating essential electron transfer pathways for PMS activation and contaminant degradation. This strategic consideration of matching catalyst characteristics with pollutant electronic structures offers critical theoretical insights for the rational design of highly efficient Fenton‐like reactions.

The application potential of these catalysts was further evaluated in various water matrices and a continuous‐flow device. Specifically, Fe_1_‐N_3_B_1_ presented better anti‐interference from the complex matrices and wider pH tolerance (pH = 3–11) for 4‐CP degradation than other catalysts (Figure 5b; Figures S27–S29, Supporting Information). This was consistent with that Fe_1_‐N_3_B_1_ held the promise of a dynamic nonradical network, and ternary activation pathways (^1^O_2_, Fe^IV ^= O, and ETP) allowed more selective degradation, better stability, and higher mineralization. This was further verified in continuous‐flow treatments, where catalysts were supported on a mesh base for uniform catalyst‐reactant contact (Figure 5c; Figure S30, Supporting Information). After 60 cycle equivalents of continuous operation treating wastewater, Fe_1_‐N_3_B_1_ could still possess near‐complete pollutant degradation performance in deionized water, groundwater, and wastewater (Figure 5d; Figure S31, Supporting Information). All the catalysts maintained stable activity in the continuous‐flow reactor even with an industrial wastewater matrix (Figure 5e). Notably, Fe_1_‐N_3_B_1_ also sustained effective 4‐CP degradation and significant TOC removal during 60‐cycle equivalents of treatments, even when the PMS concentration was reduced tenfold (Figure 5f). This long‐term performance with high stability was confirmed by the characterizations (e.g., XRD and FTIR) of recovered catalysts, which showed their excellent structural integrity (Figure S32, Supporting Information). Limited Fe was leached during the continuous treatments (consistently below 0.2%), and the ecotoxicity assessment using luminescent bacteria revealed a reduction in toxicity for all treated water samples (Figure S33, Supporting Information), preventing secondary contamination risks. In conclusion, these findings collectively establish *p*‐block element‐coordinated asymmetric Fe SACs as a superior catalytic system, distinguished by their high efficacy, exceptional operational robustness, and minimal environmental footprint.

## 3 Conclusion

This study resolves the longstanding kinetic‐thermodynamic dichotomy in PMS‐based advanced oxidation processes by the strategic design of asymmetrically coordinated Fe_1_‐N_3_X_1_ SACs. Localized charge asymmetry at Fe sites was engineered by incorporating *p*‐block elements, which orchestrated a tandem reaction network that combined rapid electron transfer and persistent ^1^O_2_/Fe^IV ^= O pathways to degrade pollutants synergistically. Crucially, the electronegativity of *p*‐block elements directly modulated Fe *d*‐band centers, optimizing PMS adsorption at the thermodynamic sweet spot. The optimized catalyst (i.e., Fe_1_‐N_3_B_1_) maintained high mineralization (≈85%) even at ultralow PMS dosages (0.05 mM), addressing both energy and cost barriers in practical environmental remediation. By decoding the interplay between coordination symmetry and oxidative pathway dynamics, this work not only delivers a transformative strategy for adaptive degradation networks but also offers a generalizable framework for the rational design of advanced oxidation systems that transcend single‐pathway constraints. These insights open avenues for engineering multi‐functional SACs tailored to complex environmental challenges (Figure S34, Supporting Information).

## 4 Experimental Section

### Chemicals and Reagents

Melamine (MA, C_3_H_6_N_6_, 99%, CAS: 108−78−1), cyanuric acid (CA, C_3_H_3_N_3_O_3_, 98%, CAS: 108−80−5), oxalic acid (OA, C_3_H_6_N_6_, 99%, CAS: 144−62−7), sulfuric acid (H_2_SO_4_, AR, 95–98%, CAS: 7664‐93‐9), boric acid (H_3_BO_3_, AR, 99.5%, CAS:10043‐35‐3), phosphoric acid (H_3_PO_4_, AR, CAS: 7664‐38‐2), ferrous sulfate heptahydrate (FeSO_4_•7H_2_O, 99.95%, CAS: 7782‐63‐0), methyl alcohol (MeOH, CH_3_OH, AR, CAS: 67−56−1), ethanol (C_2_H_5_OH, AR, CAS: 64−17−5), tert‐butanol (TBA, C_4_H_10_O, AR, CAS: 75−65−0), nitrotetrazolium blue chloride (NBT, 98%, CAS: 298‐83‐9), hydroxybenzoic acid (C_7_H_6_O_3_, AR, 98%, CAS: 99−96−7), benzoic acid (C_7_H_6_O_2_, AR, 98%, CAS: 65−85−0), *p*‐benzoquinone (C_7_H_6_O_3_, AR, 98%, CAS: 106−51−4), methyl phenyl sulfoxide (PMSO, AR, 99%, CAS: 1193−82−4), methyl phenyl sulfone (PMSO_2_, AR, 99%, CAS: 3112−85−4), cobalt chloride hexyahydrate (CoCl_2_•6H_2_O, AR, CAS: 7791−13−1), potassium peroxymonosulfate (PMS, KHSO_5_•0.5KHSO_4_•0.5K_2_SO_4_, 99%, CAS: 70693−62−8), 1, 3‐diphenylisobenzofuran (DPBF, CAS: 5471−63−6), Bisphenol A (BPA, C_15_H_16_O_2_, 99%, CAS: 80−05−7), 4−Hydroxybenzoic Acid (HBA, C_7_H_6_O_3_, 98%, CAS: 99−96−7), 4−chlorophenol (4−CP, C_6_H_5_ClO, 99%, CAS: 106−48−9), ciprofloxacin (CIP, C_17_H_18_FN_3_O_3_, 98%, CAS: 85721−33−1), tetracycline (TC, C_22_H_24_N_2_O_8_, 98%, CAS: 60−54−8), 4−nitrobenzoic Acid (NBA, C_7_H_5_NO_4_, 98%, CAS: 62−23−7), 2,4−dichlorophenol (2,4−DCP, C_6_H_4_Cl_2_O, 99%, CAS: 120−83−2), phenol (PhOH, C_6_H_6_O, 99%, CAS: 108−95−2), norfloxacin (NOR, C_16_H_18_FN_3_O_3_, 98%, CAS: 70458−96−7), levofloxacin (LEV, C_18_H_20_FN_3_O_4_, 98%, CAS: 100 986−85−4), nitrobenzene (NB, C_6_H_5_NO_2_, 99%, CAS: 98−95−3), 4−nitrophenol (PNP, C_6_H_5_NO_3_, 99%, CAS: 100−02−7) were purchased from Aladdin Biochemical Technology Co., Ltd (Shanghai, China). 5,5−dimethyl−1−pyrroline−N−oxide (DMPO, CAS: 3117−61−1), 2,2,6,6−tetramethyl piperidinyloxyl (TEMP, CAS: 33973−59−0) were purchased from Dojindo Laboratories (Shanghai, China) Co., Ltd. Sodium azide (NaN_3_, AR, CAS: 26628−22−8) and 2,2−azino−bis(3−ethylbenzonthiazoline)−6−sulfonic acid diammonium (ABTS, 99%, CAS: 30931−67−0) were purchased from Sigma−Aldrich (USA). Acetonitrile (CAS: 75−05−8) and methanol (for high−performance liquid chromatography (HPLC)) were purchased from Sinopharm.

### Synthetic Methods—Synthesis of Fe_1_‐N_4_

MA (24 mmol) and CA (19.2 mmol) were dissolved in 300 and 360 mL DI water at 90 °C for 30 min to obtain solutions A and B, respectively. FeSO_4_•7H_2_O (1.2 mmol) and OA (4.8 mmol) were dissolved in 90 mL of DI water with vigorous magnetic stirring at room temperature for 10 min to obtain solution C. Next, solution C was added to solution B. After 5 min of stirring, the mixed solution was added to solution A with continuous stirring for 4 h at room temperature. The supermolecule precursor was collected by suction filtration and dried at 60 °C overnight. After calcination under an Ar atmosphere at 600 °C for 4 h with a heating rate of 5 °C min^−1^, the Fe_1_‐N_4_ was obtained.

### Synthetic Methods—Synthesis of Fe_1_‐N_1_X_3_

MA (24 mmol) and CA (9.6 mmol) were dissolved in 300 and 360 mL DI water at 90 °C for 30 min to obtain solutions A and B, respectively. FeSO_4_•7H_2_O (1.2 mmol), H_2_SO_4_ (H_3_PO_4_, H_3_BO_3_)(9.6 mmol), and OA (4.8 mmol) were dissolved in 90 mL of DI water with vigorous magnetic stirring at room temperature for 10 min to obtain solution C. Next, solution C was added to solution B. After 5 min of stirring, the mixed solution was added to solution A with continuous stirring for 4 h at room temperature. The supermolecule precursor was collected by suction filtration and dried at 60 °C overnight. After calcination under an Ar atmosphere at 600 °C for 4 h with a heating rate of 5 °C min^−1^, the Fe_1_‐N_3_X_1_ was obtained. The Fe_1_‐N_3_X_1_ with different Fe and X loading were obtained by changing the amount of H_2_SO_4_ (H_3_PO_4_, H_3_BO_3_) and FeSO_4_•7H_2_O.

### Characterizations

A spherical aberration corrector high‐angle annular dark‐field image was acquired with a FEI Spectra 300 STEM instrument. A transmission electron microscope (TEM) was acquired with JEM‐2100F. The Fe and *p*‐block elements (S, P, B) content of the as‐prepared catalysts was examined by inductively coupled plasma mass spectrometry (ICP‐OES, ThermoICP6300, UK). XRD patterns were recorded on a Bruker D8 Advance diffractometer with Cu‐Kα radiation (*λ* = 0.1541 nm) in the range of 2*θ* from 10° to 90°. FTIR spectra were obtained with a NICOLET iS50FT‐IR spectrometer. XPS measurements were conducted on a Thermal Escalab 250Xi electron spectrometer with an Al Kα radiation X‐ray source (*hν* = 1486.6 eV). EPR spectra were obtained from Bruker A300 X‐band EPR. The photoelectric properties of the prepared samples were evaluated with a CHI660E electrochemical workstation (Chenhua, China). Cl^−^ concentration in solution was determined using ion chromatography (IC, Thermo Fisher Scientific AQUION RFIC, USA). TOC removal was measured using a TOC analyzer (Shimadzu TOC‐V, Japan). The Fe K‐edge X‐ray absorption spectra were recorded at the RapidXAFS 2 m (Anhui Absorption Spectroscopy Analysis Instrument Co., Ltd, China) using a transmission mode. The Detailed preparation method of samples was as follows: 1) The Fe content of the sample and other information were inputted in the software SAMPLEM4M to calculate the required quality of the tablet. 2) Used the tablet press to make thin sheets in the mold. 3) Wrapped the sheet in the central hollow plate with tape. To ensure the sample height and photon number meet the test requirements, the Fe content in the test samples was similar.

### Experimental Methods

The batch experiments were conducted in 50 mL beakers containing a 0.1 mm 4−CP solution (or other target contaminants) and 0.5 g L^−1^ catalysts. The mixtures were ultrasonically dispersed and then continuously magnetically stirred for 30 min to achieve adsorption–desorption equilibrium. Subsequently, 0.5 mm PMS was added as an oxidant to initiate the reaction. Samples were collected periodically and immediately quenched by the addition of methanol. The collected samples were then filtered through a 0.22 µm membrane. The concentrations of target contaminants, including BPA, 2,4−DCP, PhOH, 4−CP, HBA, PNP, NB, NBA, TC, CIP, NOX, LEV, PMSO, and PMSO_2_ were determined using a high‐performance liquid chromatography (HPLC) system (Shimadzu LC‐20A, Japan). Detailed analytical procedures are provided in Table S6 (Supporting Information). Samples were analyzed with a flow rate of 1 mL min^−1^ and an injection volume of 20 µL.

The production of superoxide radical (O_2_ **^·^** ^−^) was quantified using the NBT method. Sulfate radical (SO_4_^•−^) was indirectly determined by measuring the concentration of BQ, a major degradation byproduct of HBA. High‐valent iron‐oxo species (Fe^IV ^= O) were identified via the formation of PMSO_2_ from PMSO, which served as a chemical probe. Singlet oxygen (^1^O_2_) was detected using 1,3‐diphenylisobenzofuran (DPBF) as a trapping agent. The PMS concentration was measured by an ABTS colorimetric method.

Subsequently, the quantitative contributions of ·OH, SO_4_ **^·^** ^−^, O_2_ **^·^** ^−^, Fe^IV ^= O, and ^1^O_2_ were further assessed. A certain amount of all scavengers such as k_1_(·OH, MeOH) = 1.6–7.7 × 10^7^ m ^−1^ s^−1^, k_2_(SO_4_^·−^, MeOH) = 1.2–2.8 × 10^9^ m ^−1^ s^−1^), TBA (k_1_(**·**OH, TBA) = 6 × 10^8^ m ^−1^ s^−1^), k_1_(O_2_^·−^, NBT) = 5.88 × 10_4_ m ^−1^ s^−1^), and k_1_(^1^O_2_, NaN_3_) = 1 × 10^9^ m ^−1^ s^−1^)which were calibrated by the molar ratio with PMS to distinguish the participation of ·OH, SO_4_^·−^, O_2_^·−^, and ^1^O_2_.

For pH effect studies, the initial pH of the solution was adjusted to 3, 5, 9, or 11 before the addition of the catalyst and PMS. Coexisting ions (Na^+^, K^+^, Ca^2+^, Mg^2+^, Cl^−^, SO_3_^2−^, and HCO_3_^−^) were introduced at a concentration of 10 mM each. For real water matrix experiments, deionized (DI) water was replaced by an equal volume of lake water (from West Lake), groundwater (from the West Lake underground aquifer), and wastewater (printing and dyeing wastewater and effluent from secondary sedimentation tanks of sewage treatment plants), while keeping all other experimental conditions constant. The detailed physicochemical properties of these water samples are provided in Table S7 (Supporting Information). All batch experiments were conducted in triplicate, and the results were presented as average values with corresponding standard deviations. A 200 mg catalyst was placed on a ≈9 cm × 9 cm polyethersulfone (PES) membrane (0.22 µm pore size), which was supported by filter paper to prevent catalyst dislodgement during the catalytic reaction. The 1 mM PMS solution and deionized water were separately pumped into a continuous flow reactor. Samples were collected every 30 min to assess degradation, and the concentration of leached iron ions from the catalyst was determined by ICP‐OES. For long‐term stability studies, the experimental conditions were consistent with the continuous flow reactor experiments, with the PMS solution concentration increased to 2 mM in wastewater.

Electrochemical measurements were performed using a CHI 660E electrochemical workstation equipped with a three‐electrode system, employing techniques such as chronoamperometry (*i–t* curve), linear sweep voltammetry, EIS, and Tafel analyses. For working electrode preparation, a homogeneous dispersion was prepared by sonicating a mixture of 5 mg of catalyst, 40 µL Nafion solution, and 960 µL isopropanol. Subsequently, 100 µL of this dispersion was drop‐cast onto carbon cloth and allowed to dry completely at room temperature, forming the working electrode. A platinum sheet electrode served as the counter electrode, and an Ag/AgCl electrode was used as the reference electrode. A 0.5 m sodium sulfate solution served as the electrolyte. The reaction system initially contained 50 mL of electrolyte solution. At *t* = 240 s, the PMS solution was introduced, followed by the addition of the 4‐CP solution at *t* = 480 s.

### Calculation Methods—Nearest Neighbor (NN) Distance Distributions

The dispersion of Fe atoms on the catalysts was further assessed by comparing the measured and theoretical (random dispersion) nearest neighbor (NN) distance distributions between Fe atoms.1, 2 First, the images of AC‐HAADF‐STEM images of each catalyst were filtered by Laplacian‐of‐Gaussian filter to enhance the atom contrast, then the Gaussians were fitted as the single Fe atom in AC‐HAADF‐STEM images and the center of Gaussians were set as the Fe atom locations in next analyses. The areal density (marked as *λ*) was calculated by counting the number of Fe atoms in the selected images and dividing by the corresponding area. If all Fe atoms were randomly deposited on the surface of the CN support, the locations of Fe atoms would be described by a Poisson random field, and the probability density functions (PDF) for the distribution of NN distances (marked as *r*) should follow a Rayleigh distribution as Equation (1):

(1) $$\mathrm{PDF}\left(r\right)\;=\;2\pi \lambda r\times \exp\left(\pi \lambda r^{2}\right)$$

where the mean value of the distribution was given by (*λ* ^−1/2^)/2. By comparing the experimental NN distances of Fe atoms to the Rayleigh distribution, the degree of clustering of Fe atoms in each catalyst could be evaluated.

### Calculation Methods—The Degradation Kinetics Constant Rate for the Degradation of the Pollutant

The degradation kinetics of the pollutant was evaluated using a pseudo−first−order kinetic model, as described by the following equation:

(2) $$\ln\left(C_{t}/C_{0}\right)=-k_{obs}\;\times \;t$$

The per−site *k* values can be calculated using:

(3) $$k_{\mathrm{per}-\mathrm{site}}=k_{\mathrm{obs}}\;\times \;M/\left(m\;\times \;\mathrm{wt}.\%\right)$$

where *C_t_* is the pollutant concentration at a certain reaction time (*t*) and C_0_ is the initial pollutant concentration. *k*_obs_ is the apparent kinetics rate constant, *M* is the relative atomic mass, *m* is the catalyst's mass, and wt.% is the metal ions content determined by ICP‐OES data.

### Calculation Methods—Measurements and Calculating the Contribution of the Active Species, Generative Selectivity, and PMS Utilization

The PMS concentration was quantified by measuring its absorbance at 415 nm after adding ABTS and cobalt chloride. Singlet oxygen (^1^O_2_) concentration was determined by monitoring the consumption of 1,3‐diphenylisobenzofuran (DPBF), which served as a specific ^1^O_2_ trapping agent. Fe^IV ^= O concentrations were quantified by HPLC analysis of their characteristic reaction products. (Detailed HPLC methods are presented in Table S6, Supporting Information). Furthermore, the individual contributions of ·OH, SO_4_^·−^, O_2_^·−^, Fe^IV ^= O, and ^1^O_2_ were assessed through scavenging experiments. Reaction rate constants were defined as *k*_0_ (without a quenching agent), and *k*_1_, *k*_2_ in the presence of NaN_3_ and PMSO, respectively. The contributions (*λ*) of ^1^O_2_, Fe^IV ^= O, and ETP were subsequently calculated according to Equations (4)–(6).
(4)

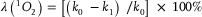

(5) $$\lambda \left(Fe^{\mathrm{IV}}=O\right)=\left[\left(k_{0}\;-k_{2}\right)\;/k_{0}\right]\;\times \;100\%$$

(6)

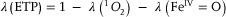

where *λ*(Fe^IV ^= O) and *λ*(^1^O_2_) are the contributions of Fe^IV ^= O and ^1^O_2_ to the degradation of 4−CP, respectively. The PMS utilization was calculated using Equation (7) below

(7) $$\mathrm{PM}S_{\mathrm{utilization}}\left(\%\right)=\sum \left(\mathrm{ROS}\right)/C\left(\mathrm{PM}S_{\mathrm{comsumption}}\right)$$

where ∑(ROS) is the generation of ROS (i.e., Fe^IV ^= O, and ^1^O_2_), and C(PMS consumption) is the consumption of PMS at certain reaction times.

### Calculation Methods—DFT Calculations

All calculations were carried out based on density functional theory (DFT) as implemented in the Vienna ab initio simulation package with the exchange‐correlation functional of generalized gradient approximation of Perdew, Burke, and Ernzerhof method. A grid of 3 × 3 × 1 Monkhorst–Pack k‐points was used for the structural relaxation.

A vacuum layer of 15 Å was adopted in the direction perpendicular to the monolayer surface to avoid the interactions between periodic slabs. The energy cutoff was set to be 520 eV. The convergence criterion for the energy and maximum force for the optimization was set to 10^−5^ eV and 0.05 eV Å^−1^, respectively. The adsorption ability and stability with the surface were evaluated by comparing the adsorption energy, the adsorption energy is defined as:

(8) $$E_{\mathrm{ads}}=E_{\mathrm{adsorb}/\mathrm{surf}\;}-\;E_{\mathrm{surf}\;}\;-\;E_{\mathrm{adsorb}}$$

where *E*_adsorb/surf_, *E*_surf_, and *E*_adsorb_ are the calculated total energies of the substrate with adsorbate(s), the clean substrate, and the isolated adsorbate, respectively.

To further explain the relative reaction trend of Li_2_Sn and S_8_ species, the free energy changes were calculated according to the following equation,

(9) $$\Delta G=\Delta E+\Delta E_{\mathrm{ZPE}}\;-\;T\Delta S$$

where Δ*E*, Δ*E_ZPE_*, and Δ*S* are the differences in total energy, zero‐point energy, and entropy between the product and reactants, respectively. Here, the ground states of S‐containing species at the temperature of 0 K were considered. So, the contribution from the entropy term was zero.

### Calculation Methods—Electrophilic Index Calculation Method

The chemical potential (*μ*), which reflects the tendency of a molecule to exchange electrons with its environment, can be approximated using the energies of the highest occupied molecular orbital (HOMO, HOMO) and the lowest unoccupied molecular orbital (LUMO, ELUMO). The chemical potential (*μ*) was calculated according to Equation (10):

(10) $$\mu \;\approx \;\left(E_{\mathrm{HOMO}}+E_{\mathrm{LUMO}}\right)/2$$

The chemical hardness (*η*), describing a molecule's resistance to electron cloud deformation, the chemical hardness (*η*) is calculated as:

(11) $$\eta \;\approx \;E_{\mathrm{LUMO}}\;-\;E_{\mathrm{HOMO}}$$

The electrophilicity index (*ω*) is calculated using the formula:

(12) $$\omega =\mu^{2}/\left(2\eta \right)$$

where *μ* is the chemical potential and *η* is the chemical hardness.

## Conflict of Interest

The authors declare no conflict of interest.

## Author Contributions

This project was conceived and supervised by J.X. and X.H.X. Y.C. performed the synthesis, characterization, and catalytic performance tests. X.H.J., Z.Y.P., C.H.L., C.L., and S.K.Z. assisted with catalyst synthesis and the execution of experiments. Z.J.L. and C.H.C. guided computational methods and EXAFS tests. All authors discussed the results and commented on the manuscript.

## Supporting information



Supporting Information

## Data Availability

The data that support the findings of this study are available from the corresponding author upon reasonable request.
