# Supplementary material for: Sabatier‐Adjusted d‐Band Centers of Scalable Asymmetric Iron Sites toward Dynamic Nonradical Network for Fast Mineralization with Low‐Amount Oxidant
Source: Adv Sci (Weinh). 2025 Nov 25;13(8):e15593. doi: 10.1002/advs.202515593 (PMC12884809; doi:10.1002/advs.202515593)
Supplement: Supplementary file 1 — Supporting Information [file ADVS-13-e15593-s001.pdf]

Supplementary Information

Sabatier-Adjusted *d*-Band Centers of Scalable Asymmetric Iron Sites toward Dynamic Nonradical Network for Fast Mineralization with Low-Amount Oxidant

Yue Chen, Xunheng Jiang, Zhiyu Pan, Zhenjie Li, Chaohuang Chen, Can Li, Chenghui Luo, Shengkun Zhang, Daohui Lin, Xinhua Xu, Jiang Xu**

Y. Chen, X. H. Jiang, Z. Y. Pan, Z. J. Li, C. H. Chen, C. Li, C. H. Luo, S. K. Zhang, D. H. Lin, X. H. Xu, J. Xu.

State Key Laboratory of Soil Pollution Control and Safety, Zhejiang University, Hangzhou 310058, China

E-mail: xujiang6@zju.edu.cn. xuxinhua@zju.edu.cn

Y. Chen, X. H. Jiang, Z. Y. Pan, Z. J. Li, C. H. Chen, C. Li, C. H. Luo, S. K. Zhang, D. H. Lin, X. H. Xu, J. Xu.

College of Environmental and Resource Sciences, Zhejiang University, Hangzhou 310058, China

D. H. Lin, J. Xu.

Zhejiang Provincial Key Laboratory of Organic Pollution Process and Control, Zhejiang University, Hangzhou 310058, China

Keywords: Fe single-atom catalyst; *p*-block element-doped; asymmetrical coordination structure; nonradical network; environmental natotechnology.

This PDF file includes:

Experimental Procedures

Supplementary Figures 1 to 34

Supplementary Tables 1 to 9

References

Table of Contents

1. Experimental Procedures 4

1.1 Chemicals and reagents...4

1.2 Synthetic methods 4

1.3 Characterizations...5

1.4 Experimental methods..6

1.5 Calculation methods...7

2. Supplementary Figures 10

Figure S1. The synthesis route diagram of the catalyst. 10

Figure S2. TEM images and HAADF-TEM of Fe_1-N_3B_1, Fe_1-N_3P_1, and Fe_1-N_3S_1. 11

Figure S3. HAADF-TEM images and elemental maps of Fe_1-N_4 12

Figure S4. XRD patterns of Fe_1-N_4, Fe_1-N_3B_1, Fe_1-N_3P_1, and Fe_1-N_3B_1..13

Figure S5. FTIR spectra of the Fe_1-N_4, Fe_1-N_3B_1, Fe_1-N_3P_1, and Fe_1-N_3B_1. 14

Figure S6. WT-EXAFS plots of FePc and Fe foil 15

Figure S7. k^3-weighted Fourier transform spectra of the Fe_1-N_4, Fe_1-N_3S_1, Fe_1-N_3P_1, Fe_1-N_3B_1 ...16

Figure S8. XPS spectra of Fe 2*p* for the Fe_1-N_4, Fe_1-N_3B_1, Fe_1-N_3P_1, and Fe_1-N_3B_1......................17

Figure S9. Chemical structure models of Fe_1-N_4, Fe_1-N_3B_1, Fe_1-N_3P_1, and Fe_1-N_3B_1 systems 18

Figure S10. PDOS profiles of Fe_1-N_4, Fe_1-N_3B_1, Fe_1-N_3P_1, and Fe_1-N_3B_1 systems.......................19

Figure S11. Comparison of the degradation of 4-CP by activated PMS with different catalysts....20

Figure S12. EPR spectra of background systems and DMPO-trapped $O_2^{\cdot-}$ 21

Figure S13. Fe^{IV}=O concentration in Fe_1-N_4, Fe_1-N_3B_1, Fe_1-N_3P_1, and Fe_1-N_3B_1 systems............22

Figure S14. Quantitative experiments of Fe^{IV}=O within 10 minutes...23

Figure S15. The consumption of PMS during the reaction process..24

Figure S16. PMS utilization rate experiment of reaction time...25

Figure S17. FTIR spectra of the Fe_1-N_3B_1 during the reaction process...26

Figure S18. PMS consumption with or without 4-CP..27

Figure S19. Degradation of 4-CP degradation by Fe_1-X with different doping ratios. 28

Figure S20. 4-CP degradation efficiency after the addition of quenchers by Fe_1............................29

Figure S21. 4-CP degradation efficiency after the addition of quenchers by Fe_1-S 30

Figure S22. 4-CP degradation efficiency after the addition of quenchers by Fe_1-P 31

Figure S23. 4-CP degradation efficiency after the addition of quenchers by Fe_1-B........................32

Figure S24. The degradation efficiency of various aromatic pollutants..33

Figure S25. The degradation efficiency of various antibiotics 34

Figure S26. Linearity between the electrophilic indexes of different pollutants and ln(k_{obs})..........35

Figure S27. The 4-CP degradation efficiency at different initial pH..36

Figure S28. The 4-CP degradation efficiency with background ions 37

Figure S29. The 4-CP degradation efficiency in different waters. 37

Figure S30. The physical diagram of the continuous flow membrane reaction device 39

Figure S31. The removal rate of 4-CP during the 600-minute device experiment..........................40

Figure S32. XRD patterns and FTIR spectra before and after the reaction. 41

Figure S33. The change in the inhibition rate of luminescent bacteria after the reaction................42

Figure S34. Summary of the results……………………………………………………………43

3. Supplementary Tables …........……………………………………………………… ….. 44

Table S1. Fe and p-block element content of samples..44

Table S2. EXAFS fitting parameters at the Fe K-edge for various samples...................................45

Table S3. The variation in the charge of elements in the Fe_1-N_4 and Fe_1-N_3X_1. 46

Table S4. A comparison of TOC in the recently reported Fenton-like catalysts 47

Table S5. A comparison of k_{obs} and loading in the recently reported catalysts. 48

Table S6. The *d*-band of Fe and the PMS* in the recently reported catalysts. 49

Table S7. Relative parameters of different pollutants...50

Table S8. The HPLC analysis conditions for different substrates. 51

Table S9. Properties of the water sample...52

4. References……... ………………..53

1. **Experimental Procedures**

1.1 Chemicals and reagents

Melamine (MA, $C_3H_6N_6$, 99%, CAS: 108−78−1), cyanuric acid (CA, $C_3H_3N_3O_3$, 98%, CAS: 108−80−5), oxalic acid (OA, $C_3H_6N_6$, 99%, CAS: 144−62−7), sulfuric acid (H_2SO_4, AR, 95.0～98.0%, CAS: 7664-93-9),boric acid (H_3BO_3, AR, 99.5%, CAS:10043-35-3), phosphoric acid (H_3PO_4, AR, CAS: 7664-38-2), ferrous sulfate heptahydrate ($FeSO_4$•$7H_2O$, 99.95%, CAS: 7782-63-0), methyl alcohol (MeOH, CH_3OH, AR, CAS: 67−56−1), ethanol (C_2H_5OH, AR, CAS: 64−17−5), tert-butanol (TBA, $C_4H_{10}O$, AR, CAS: 75−65−0), nitrotetrazolium blue chloride (NBT, 98%, CAS: 298-83-9), hydroxybenzoic acid ($C_7H_6O_3$, AR, 98%, CAS: 99−96−7), benzoic acid ($C_7H_6O_2$, AR, 98%, CAS: 65−85−0), *p*-benzoquinone ($C_7H_6O_3$, AR, 98%, CAS: 106−51−4), methyl phenyl sulfoxide (PMSO, AR, 99%, CAS: 1193−82−4), methyl phenyl sulfone ($PMSO_2$, AR, 99%, CAS: 3112−85−4), cobalt chloride hexyahydrate ($CoCl_2$•$6H_2O$, AR, CAS: 7791−13−1), potassium peroxymonosulfate (PMS, $KHSO_5$•$0.5KHSO_4$•$0.5K_2SO_4$, 99%, CAS: 70693−62−8), 1,3-diphenylisobenzofuran (DPBF, CAS: 5471−63−6), Bisphenol A (BPA, $C_{15}H_{16}O_2$, 99%, CAS: 80−05−7), 4−Hydroxybenzoic Acid (HBA, $C_7H_6O_3$, 98%, CAS: 99−96−7), 4−chlorophenol (4−CP, C_6H_5ClO, 99%, CAS: 106−48−9), ciprofloxacin (CIP, $C_{17}H_{18}FN_3O_3$, 98%, CAS: 85721−33−1), tetracycline (TC, $C_{22}H_{24}N_2O_8$, 98%, CAS: 60−54−8), 4−nitrobenzoic Acid (NBA, $C_7H_5NO_4$, 98%, CAS: 62−23−7), 2,4−dichlorophenol (2,4−DCP, $C_6H_4Cl_2O$, 99%, CAS: 120−83−2), phenol (PhOH, C_6H_6O, 99%, CAS: 108−95−2), norfloxacin (NOR, $C_{16}H_{18}FN_3O_3$, 98%, CAS: 70458−96−7), levofloxacin (LEV, $C_{18}H_{20}FN_3O_4$, 98%, CAS: 100986−85−4), nitrobenzene (NB, $C_6H_5NO_2$, 99%, CAS: 98−95−3), 4−nitrophenol (PNP, $C_6H_5NO_3$, 99%, CAS: 100−02−7) were purchased from Aladdin Biochemical Technology Co., Ltd (Shanghai, China). 5,5−dimethyl−1−pyrroline−N−oxide (DMPO, CAS: 3117−61−1), 2,2,6,6−tetramethyl piperidinyloxyl (TEMP, CAS: 33973−59−0) were purchased from Dojindo Laboratories (Shanghai, China) Co., Ltd. Sodium azide (NaN_3, AR, CAS: 26628−22−8) and 2,2−azino−bis(3−ethylbenzonthiazoline)−6−sulfonic acid diammonium (ABTS, 99%, CAS: 30931−67−0) were purchased from Sigma−Aldrich (USA). Acetonitrile (CAS: 75−05−8) and methanol (for high−performance liquid chromatography (HPLC)) were purchased from Sinopharm.

1.2 Synthetic methods

Synthesis of Fe_1-N_4

MA (24 mmol) and CA (19.2 mmol) were dissolved in 300 mL and 360 mL DI water at 90°C for 30 min to obtain solutions A and B, respectively. $FeSO_4$•$7H_2O$ (1.2 mmol) and OA (4.8 mmol) were dissolved in 90 mL of DI water with vigorous magnetic stirring at room temperature for 10 min to obtain solution C. Next, solution C was added to solution B. After 5 min of stirring, the mixed solution

was added to solution A with continuous stirring for 4 h at room temperature. The supermolecule precursor was collected by suction filtration and dried at 60°C overnight. After calcination under an Ar atmosphere at 600°C for 4 h with a heating rate of 5°C min^{-1}, the Fe_1-N_4 was obtained.

Synthesis of Fe_1-N_1X_3

MA (24 mmol) and CA (9.6 mmol) were dissolved in 300 mL and 360 mL DI water at 90°C for 30 min to obtain solutions A and B, respectively. $FeSO_4$•$7H_2O$ (1.2 mmol), H_2SO_4 (H_3PO_4, H_3BO_3)(9.6 mmol), and OA (4.8 mmol) were dissolved in 90 mL of DI water with vigorous magnetic stirring at room temperature for 10 min to obtain solution C. Next, solution C was added to solution B. After 5 min of stirring, the mixed solution was added to solution A with continuous stirring for 4 h at room temperature. The supermolecule precursor was collected by suction filtration and dried at 60°C overnight. After calcination under an Ar atmosphere at 600°C for 4 h with a heating rate of 5°C min^{-1}, the Fe_1-N_3X_1 was obtained. The Fe_1-N_3X_1 with different Fe and X loading were obtained by changing the amount of H_2SO_4 (H_3PO_4, H_3BO_3) and $FeSO_4$•$7H_2O$.

1.3 Characterizations

A spherical aberration corrector high-angle annular dark-field (AC-HAADF-TEM) image was acquired with a FEI Spectra 300 STEM instrument. A transmission electron microscope (TEM) was acquired with JEM-2100F. The Fe and *p*-block elements (S, P, B) content of the as-prepared catalysts was examined by inductively coupled plasma mass spectrometry (ICP-OES, ThermoICP6300, UK). XRD patterns were recorded on a Bruker D8 Advance diffractometer with Cu-Kα radiation (λ = 0.1541 nm) in the range of 2θ from 10° to 90°. FTIR spectra were obtained with a NICOLET iS50FT-IR spectrometer. XPS measurements were conducted on a Thermal Escalab 250Xi electron spectrometer with an Al Kα radiation X-ray source (hν = 1486.6 eV). EPR spectra were obtained from Bruker A300 X-band EPR. The photoelectric properties of the prepared samples were evaluated with a CHI660E electrochemical workstation (Chenhua, China). Cl^- concentration in solution was determined using ion chromatography (IC, Thermo Fisher Scientific AQUION RFIC, USA). Total organic carbon (TOC) removal was measured using a TOC analyzer (Shimadzu TOC-V, Japan). The Fe K-edge X-ray absorption spectra were recorded at the RapidXAFS 2 M (Anhui Absorption Spectroscopy Analysis Instrument Co., Ltd, China) using a transmission mode. The Detailed preparation method of samples is as follows: 1) Input the Fe content of the sample and other information in the software SAMPLEM4M to calculate the required quality of the tablet. 2) Use the tablet press to make thin sheets in the mold. 3) Wrap the sheet in the central hollow plate with tape. To ensure the sample height and photon number meet the test requirements, the Fe content in the test samples is similar.

1.4 Experimental methods

The batch experiments were conducted in 50 mL beakers containing a 0.1 mM 4−CP solution (or other target contaminants) and 0.5 g/L catalysts. The mixtures were ultrasonically dispersed and then continuously magnetically stirred for 30 minutes to achieve adsorption−desorption equilibrium. Subsequently, 0.5 mM PMS was added as an oxidant to initiate the reaction. Samples were collected periodically and immediately quenched by the addition of methanol. The collected samples were then filtered through a 0.22 μm membrane. The concentrations of target contaminants, including BPA, 2,4−DCP, PhOH, 4−CP, HBA, PNP, NB, NBA, TC, CIP, NOX, LEV, PMSO, and $PMSO_2$ were determined using a high-performance liquid chromatography (HPLC) system (Shimadzu LC-20A, Japan). Detailed analytical procedures are provided in Table S6. Samples were analyzed with a flow rate of 1.0 mL/min and an injection volume of 20 μL.

The production of superoxide radical ($O_2^{\cdot-}$) was quantified using the NBT method. Sulfate radical ($SO_4^{\bullet-}$) was indirectly determined by measuring the concentration of BQ, a major degradation byproduct of HBA. High-valent iron-oxo species (Fe^{IV}=O) were identified via the formation of $PMSO_2$ from PMSO, which served as a chemical probe. Singlet oxygen ($^1O_2$) was detected using 1,3-diphenylisobenzofuran (DPBF) as a trapping agent. The PMS concentration was measured by an ABTS colorimetric method.

Subsequently, the quantitative contributions of ·OH, $SO_4^{\cdot-}$, $O_2^{\cdot-}$, Fe^{IV}=O, and $^1O_2$ were further assessed. A certain amount of all scavengers such as k_1(·OH, MeOH) = 1.6-7.7 × 10^7 M^{-1} s^{-1}, k_2($SO_4^{\cdot-}$, MeOH) = 1.2−2.8 × 10^9 M^{-1} s^{-1}), TBA (k_1(·OH, TBA) = 6.0 × 10^8 M^{-1} s^{-1}), k_1($O_2^{\cdot-}$, NBT) = 5.88 × 10_4 M^{-1} s^{-1}), and k_1($^1O_2$, NaN_3) = 1.0 × 10^9 M^{-1} s^{-1}), PMSO (k_1(·OH, TBA) = 6.0 × 10^8 M^{-1} s^{-1}) which were calibrated by the molar ratio with PMS to distinguish the participation of ·OH, $SO_4^{\cdot-}$, $O_2^{\cdot-}$, and $^1O_2$.[1]

For pH effect studies, the initial pH of the solution was adjusted to 3, 5, 9, or 11 before the addition of the catalyst and PMS. Coexisting ions (Na^+, K^+, Ca^{2+}, Mg^{2+}, Cl^-, SO_3^{2-}, and HCO_3^-) were introduced at a concentration of 10 mmol/L each. For real water matrix experiments, deionized (DI) water was replaced by an equal volume of lake water (from West Lake), groundwater (from the West Lake underground aquifer), and wastewater (printing and dyeing wastewater and effluent from secondary sedimentation tanks of sewage treatment plants), while keeping all other experimental conditions constant. The detailed physicochemical properties of these water samples are provided in Table 7. All batch experiments were conducted in triplicate, and the results are presented as average values with corresponding standard deviations. A 200 mg catalyst was placed on an approximately 9 cm × 9 cm polyethersulfone (PES) membrane (0.22 μm pore size), which was supported by filter paper to prevent catalyst dislodgement during the catalytic reaction. The 1 mM PMS solution and deionized

water were separately pumped into a continuous flow reactor. Samples were collected every 30 min to assess degradation, and the concentration of leached iron ions from the catalyst was determined by ICP-OES. For long-term stability studies, the experimental conditions were consistent with the continuous flow reactor experiments, with the PMS solution concentration increased to 2 mM in wastewater.

Electrochemical measurements were performed using a CHI 660E electrochemical workstation equipped with a three-electrode system, employing techniques such as chronoamperometry (***i-t*** curve), linear sweep voltammetry (LSV), electrochemical impedance spectroscopy (EIS), and Tafel analyses. For working electrode preparation, a homogeneous dispersion was prepared by sonicating a mixture of 5 mg of catalyst, 40 μL Nafion solution, and 960 μL isopropanol. Subsequently, 100 μL of this dispersion was drop-cast onto carbon cloth and allowed to dry completely at room temperature, forming the working electrode. A platinum sheet electrode served as the counter electrode, and an Ag/AgCl electrode was used as the reference electrode. A 0.5 M sodium sulfate solution served as the electrolyte. The reaction system initially contained 50 mL of electrolyte solution. At t = 240 s, the PMS solution was introduced, followed by the addition of the 4-CP solution at t = 480 s.

1.5 Calculation methods

Nearest neighbor (NN) distance distributions

The dispersion of Fe atoms on the catalysts was further assessed by comparing the measured and theoretical (random dispersion) nearest neighbor (NN) distance distributions between Fe atoms.1, 2 Firstly, the images of AC-HAADF-STEM images of each catalyst were filtered by Laplacian-of-Gaussian filter to enhance the atom contrast, then the Gaussians were fitted as the single Fe atom in AC-HAADF-STEM images and the center of Gaussians were set as the Fe atom locations in next analyses. The areal density (marked as λ) was calculated by counting the number of Fe atoms in the selected images and dividing by the corresponding area. If all Fe atoms were randomly deposited on the surface of the CN support, the locations of Fe atoms would be described by a Poisson random field, and the probability density functions (PDF) for the distribution of NN distances (marked as r) should follow a Rayleigh distribution as Eq. (1):

$$\mathrm{PDF}(r)=2\pi\lambda r\cdot\exp(\pi\lambda r^{2}) \quad (1)$$

where the mean value of the distribution was given by $(\lambda^{-1/2})/2$. By comparing the experimental NN distances of Fe atoms to the Rayleigh distribution, we can evaluate the degree of clustering of Fe atoms in each catalyst.[2]

The degradation kinetics constant rate for the degradation of the pollutant

The degradation kinetics of the pollutant is evaluated using a pseudo−first−order kinetic model, as described by the following equation:

$$\ln(C_t/C_0) = -k_{obs} \times t \quad (2)$$

The per−site k values can be calculated using

$$k_{per-site} = k_{obs} \times M / (m \times wt\ \%). \quad (3)$$

Where C_t is the pollutant concentration at a certain reaction time (t) and C_0 is the initial pollutant concentration. k_{obs} is the apparent kinetics rate constant, M is the relative atomic mass, m is the catalyst's mass, and wt % % is the metal ions content determined by ICP-OES data.

Measurements and calculating the contribution of the active species, generative selectivity, and PMS utilization

The PMS concentration was quantified by measuring its absorbance at 415 nm after adding ABTS and cobalt chloride. Singlet oxygen ($^1O_2$) concentration was determined by monitoring the consumption of 1,3-diphenylisobenzofuran (DPBF), which served as a specific $^1O_2$ trapping agent. Fe^{IV}=O concentrations were quantified by HPLC analysis of their characteristic reaction products. (Detailed HPLC methods are presented in Table S6). Furthermore, the individual contributions of ·OH, $SO_4^{\cdot-}$, $O_2^{\cdot-}$, Fe^{IV}=O, and $^1O_2$ were assessed through scavenging experiments. Reaction rate constants were defined as k_0 (without a quenching agent), and k_1, k_2, and k_3 in the presence of TBA, MeOH, and PMSO, respectively. The contributions (λ) of $^1O_2$, Fe^{IV}=O, and ETP were subsequently calculated according to Eqs. (4−6).

$$\lambda(^1O_2) = [(k_0 - k_1) / k_0] \times 100\% \quad (4)$$

$$\lambda(Fe^{IV}{=}O) = [(k_0 - k_2) / k_0] \times 100\% \quad (5)$$

$$\lambda(\text{ETP}) = 1 - \lambda(^1O_2) - \lambda(Fe^{IV}{=}O) \quad (6)$$

where λ(Fe^{IV}=O) and λ($^1O_2$) are the contributions of Fe^{IV}=O and $^1O_2$ to the degradation of 4−CP, respectively. The PMS utilization was calculated using Eq.(7) below

$$PMS_{utilization}(\%) = \sum(ROS) / C(PMS_{comsumption}) \quad (7)$$

where ∑(ROS) is the generation of ROS (i.e., Fe^{IV}=O, and $^1O_2$), and C(PMS consumption) is the consumption of PMS at certain reaction times.[3]

DFT calculations

All calculations were carried out based on density functional theory (DFT) as implemented in the Vienna ab initio simulation package (VASP) with the exchange-correlation functional of generalized

gradient approximation (GGA) of Perdew, Burke, and Ernzerhof (PBE) method. A grid of 3 × 3 × 1 Monkhorst-Pack k-points was used for the structural relaxation.

A vacuum layer of 15 Å is adopted in the direction perpendicular to the monolayer surface to avoid the interactions between periodic slabs. The energy cutoff was set to be 520 eV. The convergence criterion for the energy and maximum force for the optimization was set to 10-5 eV and 0.05 eV/Å, respectively. The adsorption ability and stability with the surface were evaluated by comparing the adsorption energy, the adsorption energy is defined as:

$$E_{ads} = Ead_{sorb/surf} - E_{surf} - E_{adsorb} \quad (8)$$

where $E_{adsorb/surf}$, E_{surf}, and $E_{adsobare}$ are the calculated total energies of the substrate with adsorbate(s), the clean substrate, and the isolated adsorbate, respectively.

To further explain the relative reaction trend of Li_2Sn and S_8 species, the free energy changes are calculated according to the following equation,

$$\triangle G = \triangle E + \triangle EZPE - T\triangle S \quad (9)$$

where ΔE, ΔEZPE, and ΔS are the differences in total energy, zero point energy, and entropy between the product and reactants, respectively. Here, we consider the ground states of S-containing species at the temperature of 0 K. So, the contribution from the entropy term is zero.

Electrophilic index calculation method

The chemical potential (μ), which reflects the tendency of a molecule to exchange electrons with its environment, can be approximated using the energies of the highest occupied molecular orbital (HOMO, HOMO) and the lowest unoccupied molecular orbital (LUMO, ELUMO), the chemical potential (μ) was calculated according to Eq. (10):

$$\mu \approx (E_{HOMO} + E_{LUMO})/2 \quad (10)$$

The chemical hardness (η), describing a molecule's resistance to electron cloud deformation, the chemical hardness (η) is calculated as:

$$\eta \approx E_{LUMO} - E_{HOMO} \quad (11)$$

The electrophilicity index (ω) is calculated using the formula:

$$\omega = \mu^2/(2\eta) \quad (12)$$

where μ is the chemical potential and η is the chemical hardness.

2. Supplementary Figures

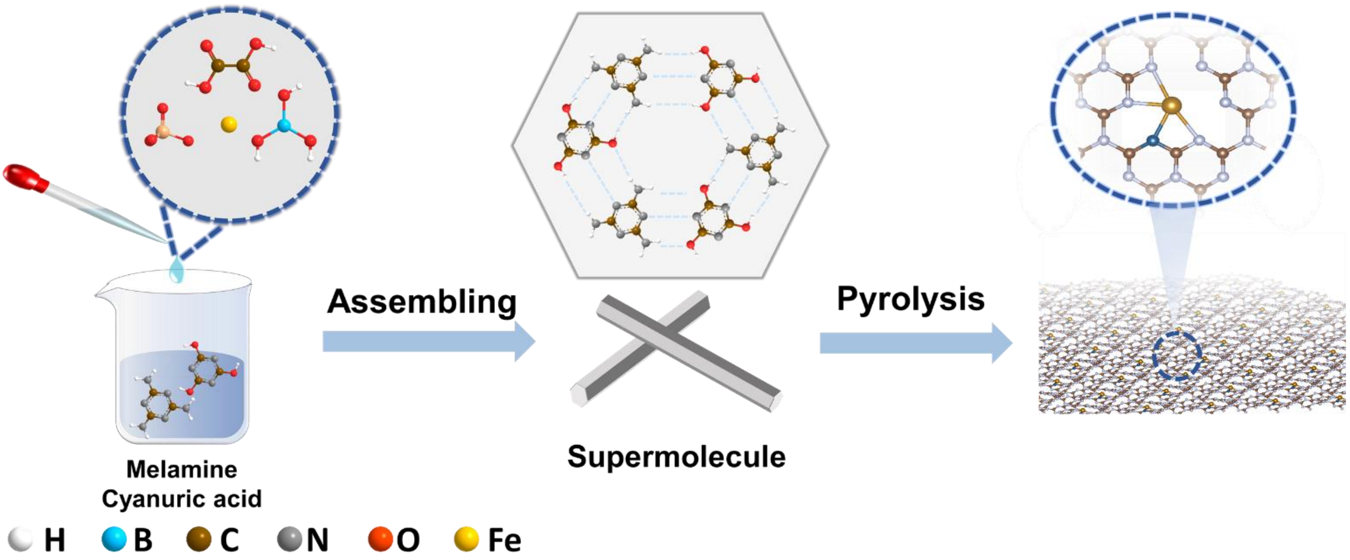

Figure S1. The synthesis route diagram of the catalyst.

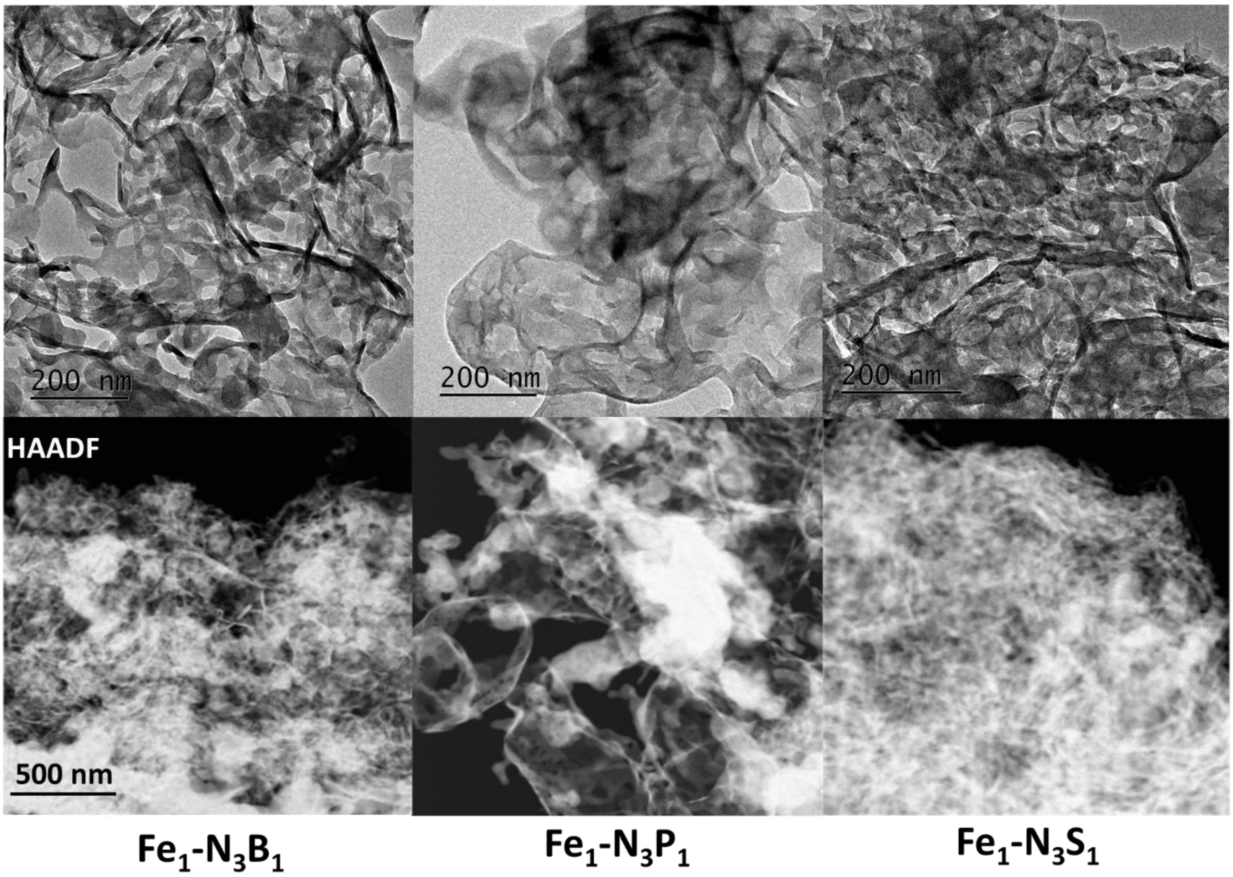

Figure S2. TEM images and HAADF-TEM of Fe_1-N_3B_1, Fe_1-N_3P_1, and Fe_1-N_3S_1.

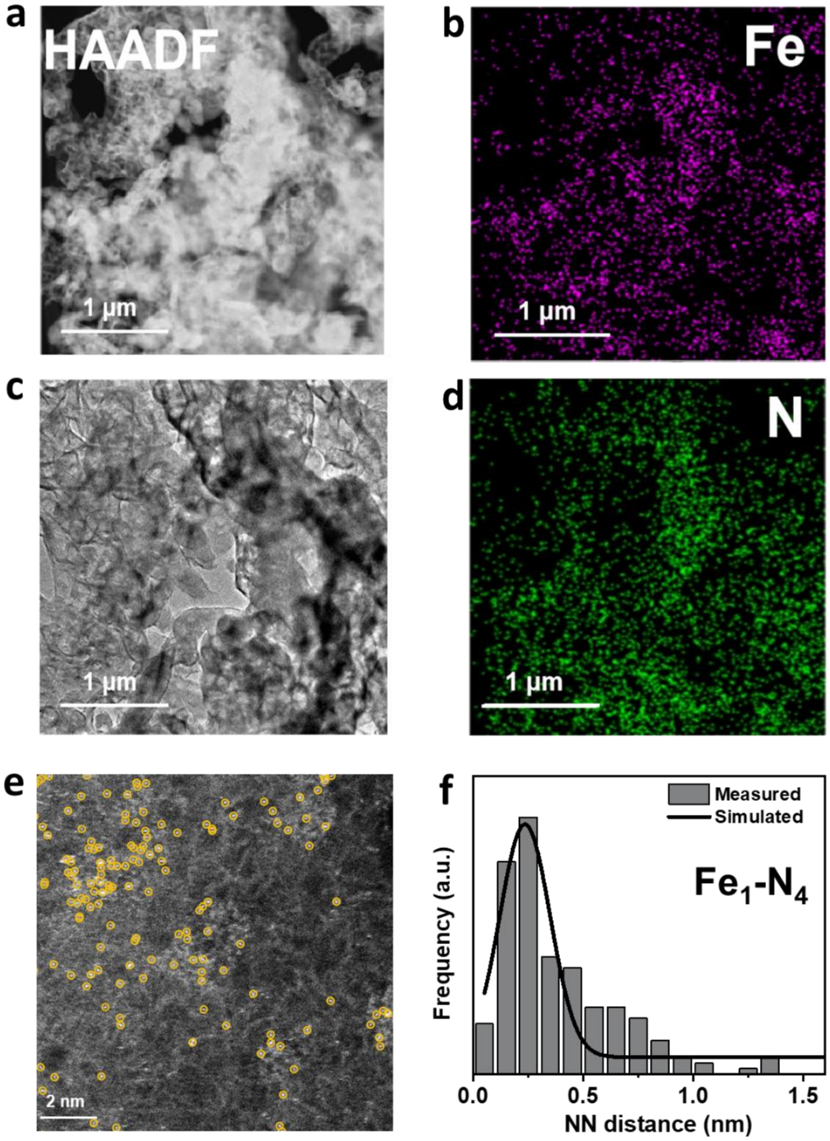

Figure S3. (a-d) HAADF-TEM images and elemental maps of Fe_1-N_4. (e)AC-HAADF-STEM images of Fe_1-N_4.(f) Corresponding Histogram and fitting of the distance distribution between neighboring Fe atoms of Fe_1-N_4.

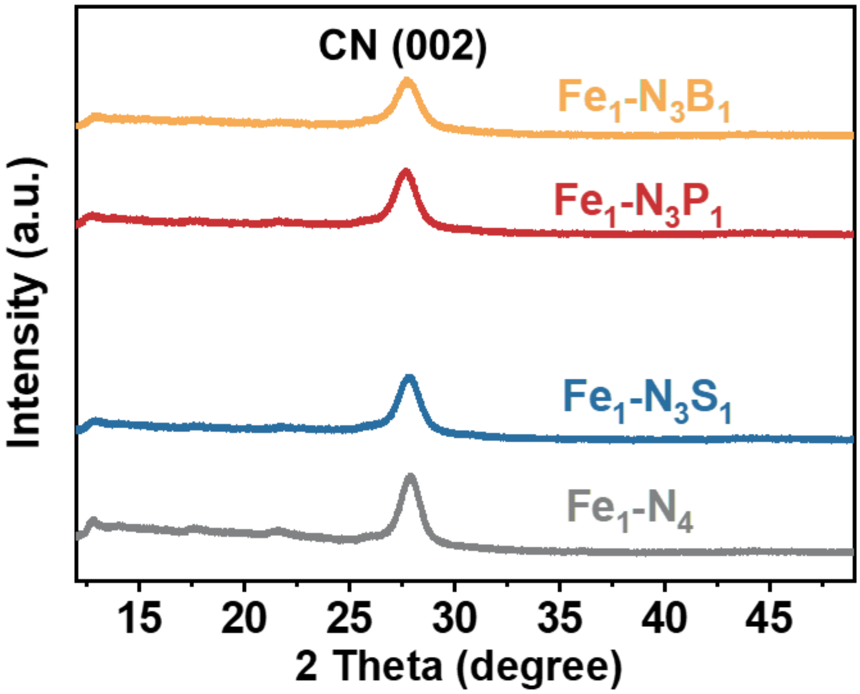

Figure S4. XRD patterns of Fe_1-N_4, Fe_1-N_3B_1, Fe_1-N_3P_1, and Fe_1-N_3B_1.

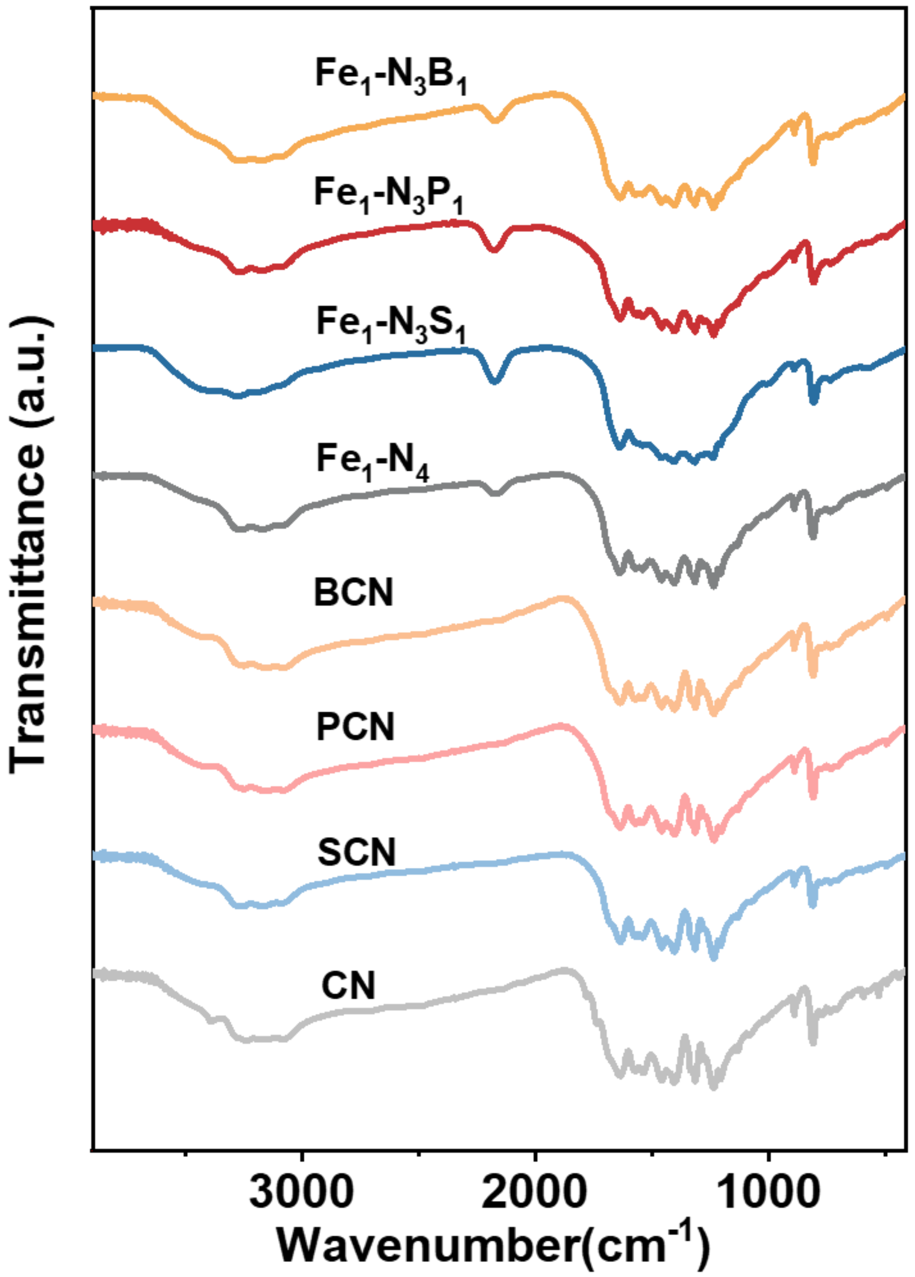

Figure S5. FTIR spectra of the CN, SCN, PCN, BCN, Fe_1-N_4, Fe_1-N_3B_1, Fe_1-N_3P_1, and Fe_1-N_3B_1.

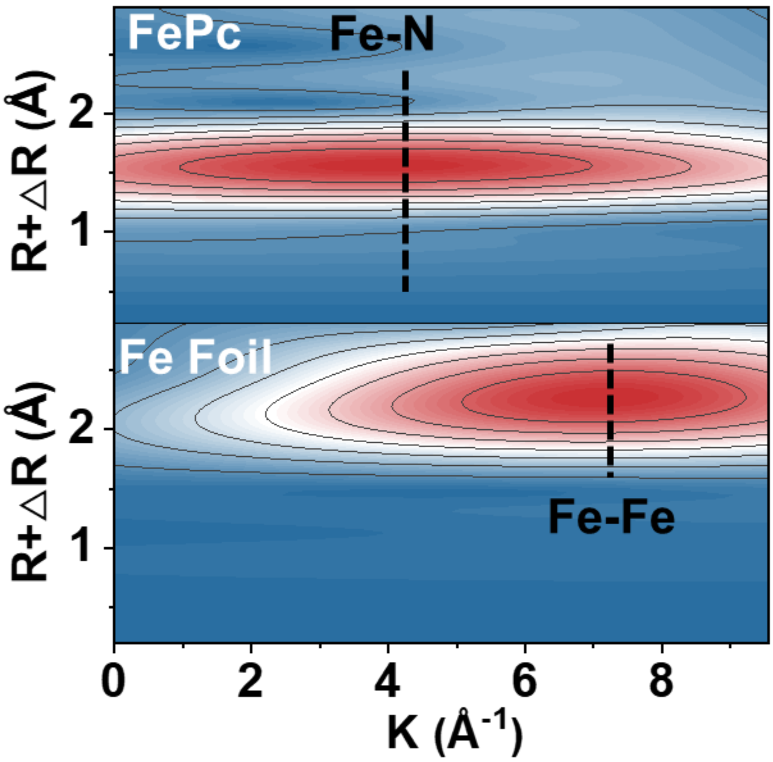

Figure S6. WT-EXAFS plots of FePc and Fe foil.

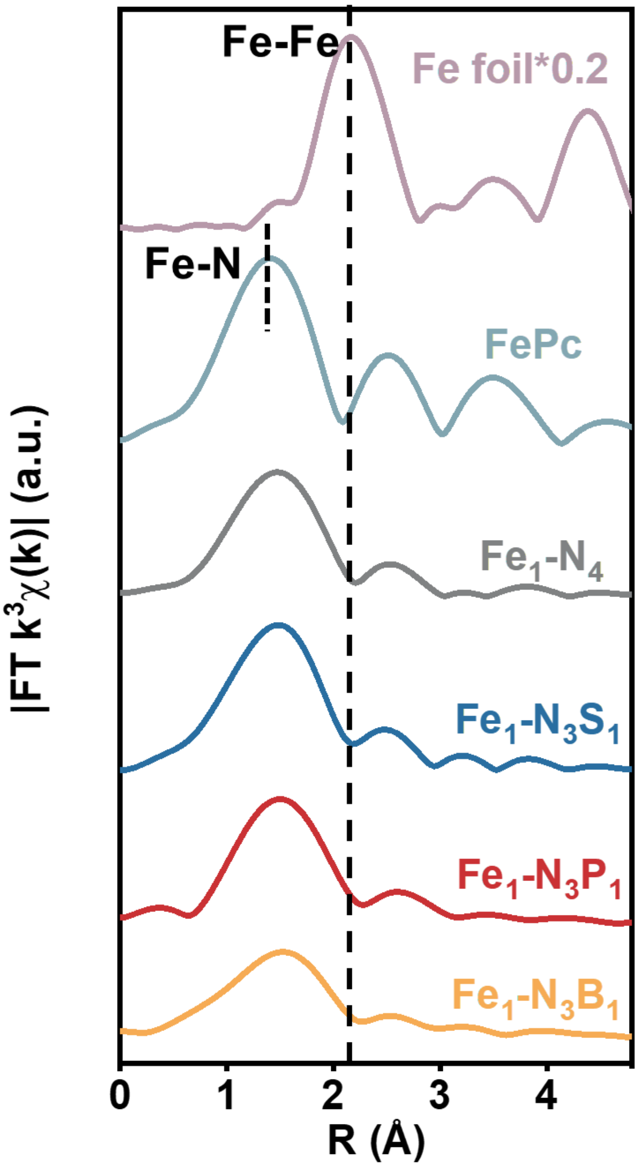

Figure S7. k^3-weighted Fourier transform spectra of the Fe_1-N_4, Fe_1-N_3S_1, Fe_1-N_3P_1, Fe_1-N_3B_1, and reference samples.

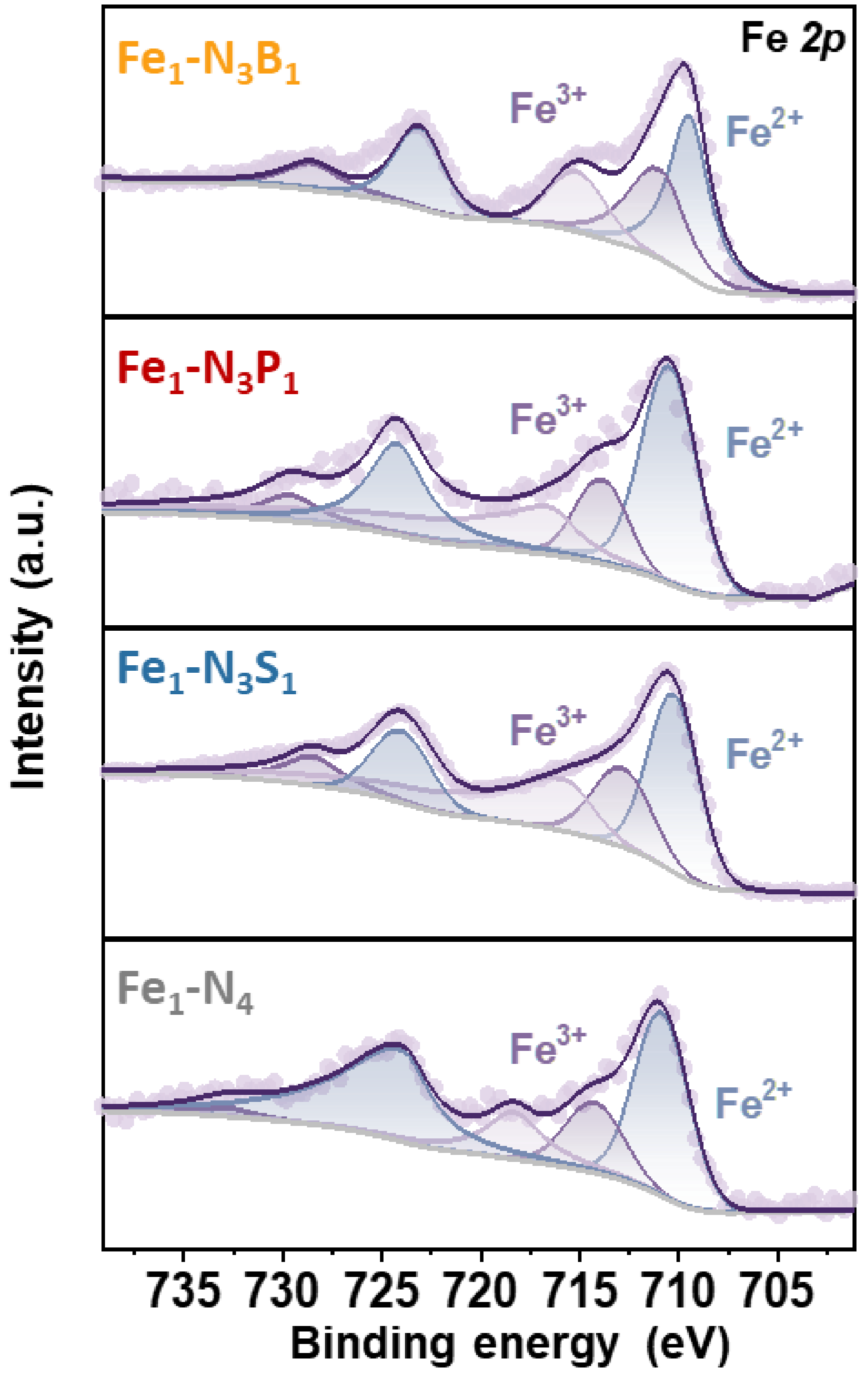

Figure S8. XPS spectra of Fe 2*p* for the Fe_1-N_4, Fe_1-N_3B_1, Fe_1-N_3P_1, and Fe_1-N_3B_1.

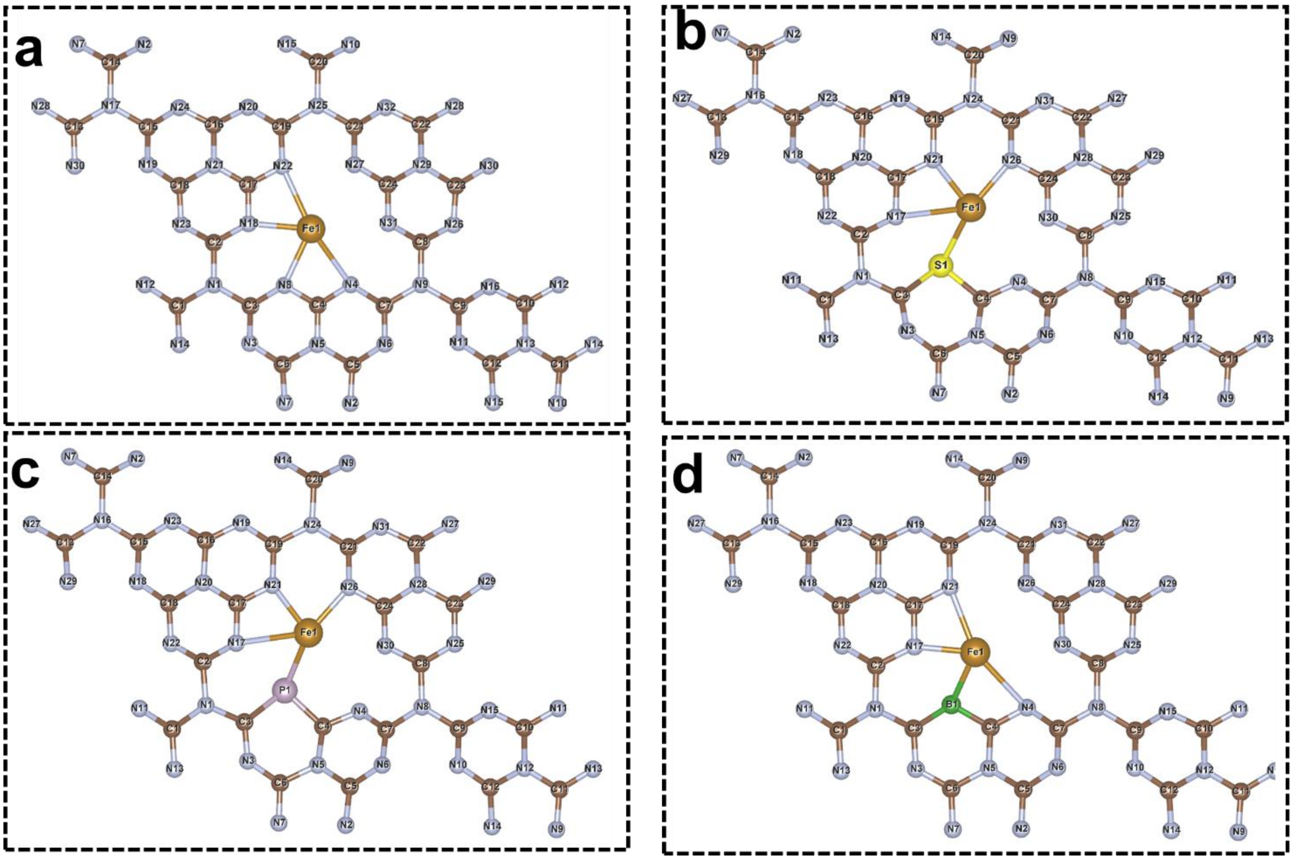

Figure S9. Chemical structure models of Fe_1-N_4, Fe_1-N_3B_1, Fe_1-N_3P_1, and Fe_1-N_3B_1 systems.

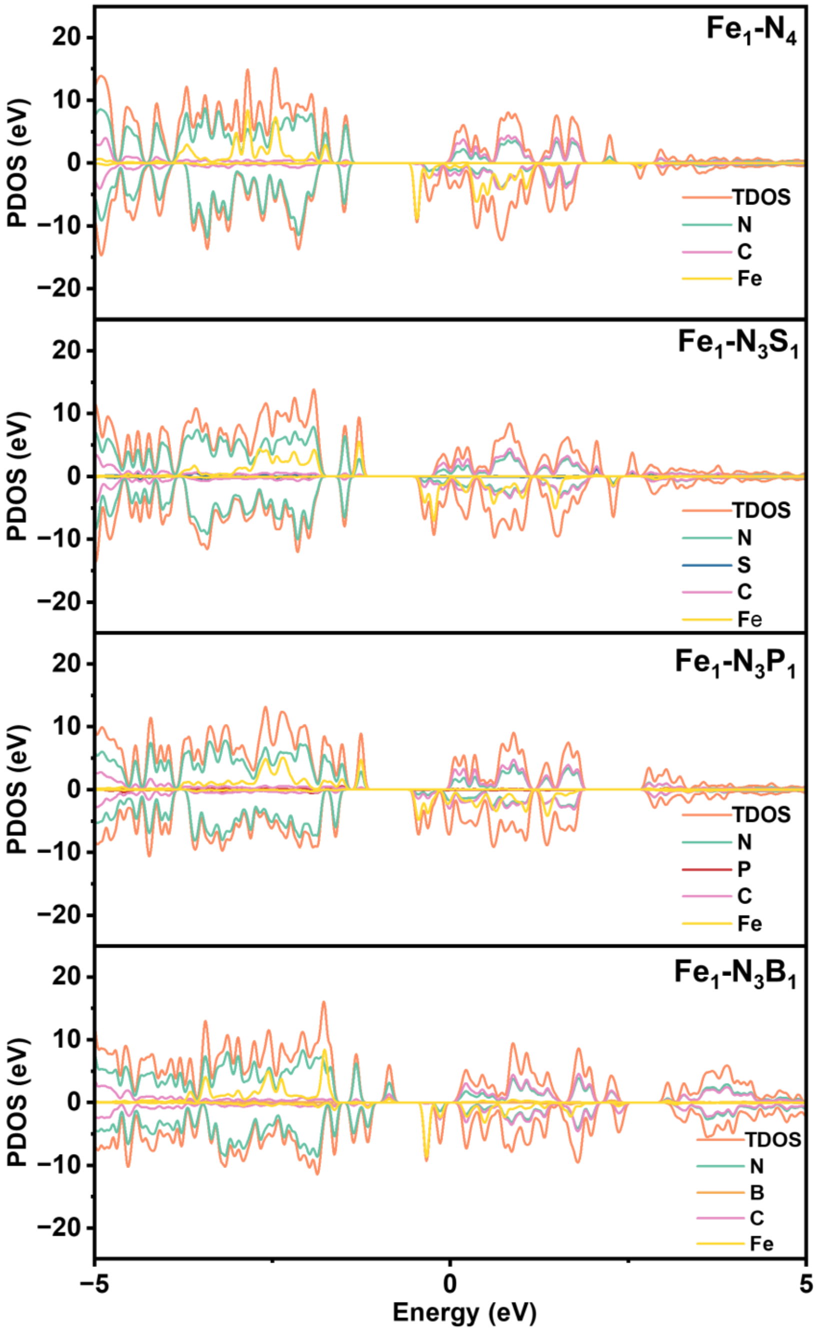

Figure S10. PDOS profiles of Fe_1-N_4, Fe_1-N_3B_1, Fe_1-N_3P_1, and Fe_1-N_3B_1 systems.

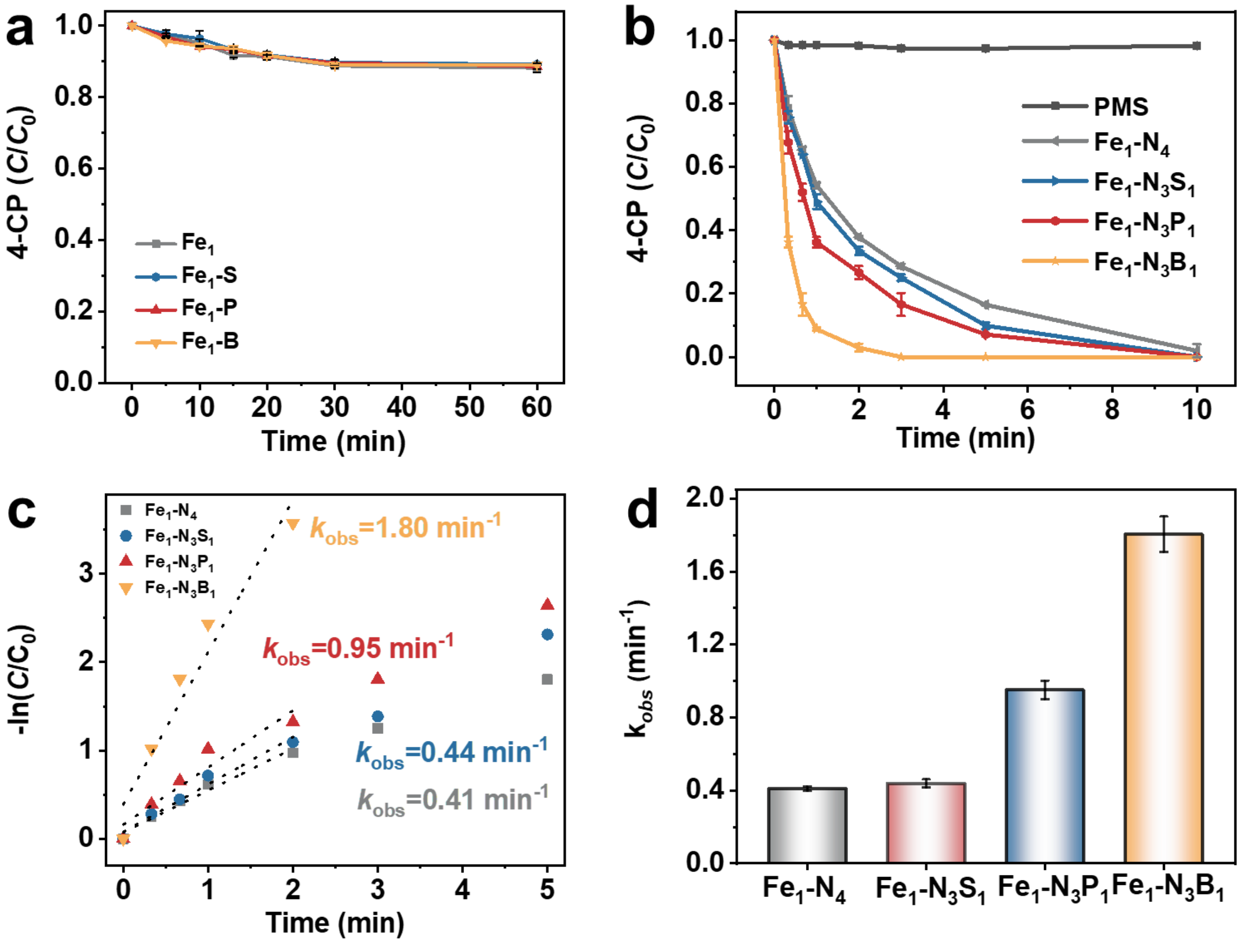

Figure S11. (a) The adsorption curve of the catalysts for 4-CP within 30 minutes, (b) Comparison of the degradation of 4-CP by activated PMS with different catalysts, (c)The pseudo-first order kinetics for different catalytic systems, and (d) The corresponding reaction rate constants. (Conditions: 4-CP = 0.1 mM, PMS = 0.5 mM, catalysts = 0.5 g L^{-1}).

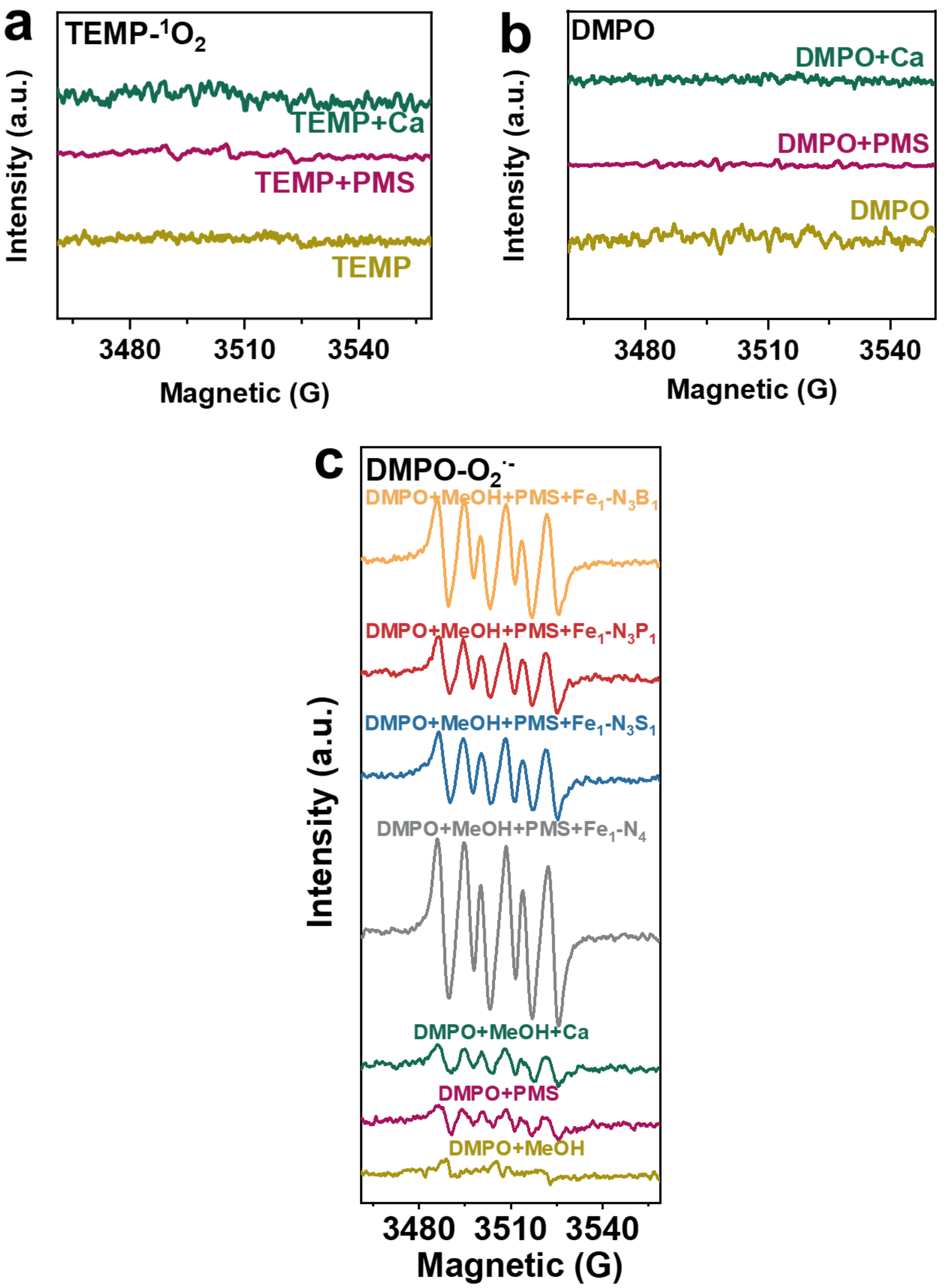

Figure S12. (a) EPR spectra of background systems. (b) DMPO-trapped $O_2^{\cdot -}$. (Conditions: PMS = 0.5 mM, catalysts = 0.5 g L^{-1}, TEMP=DMPO = 50.0 mM).

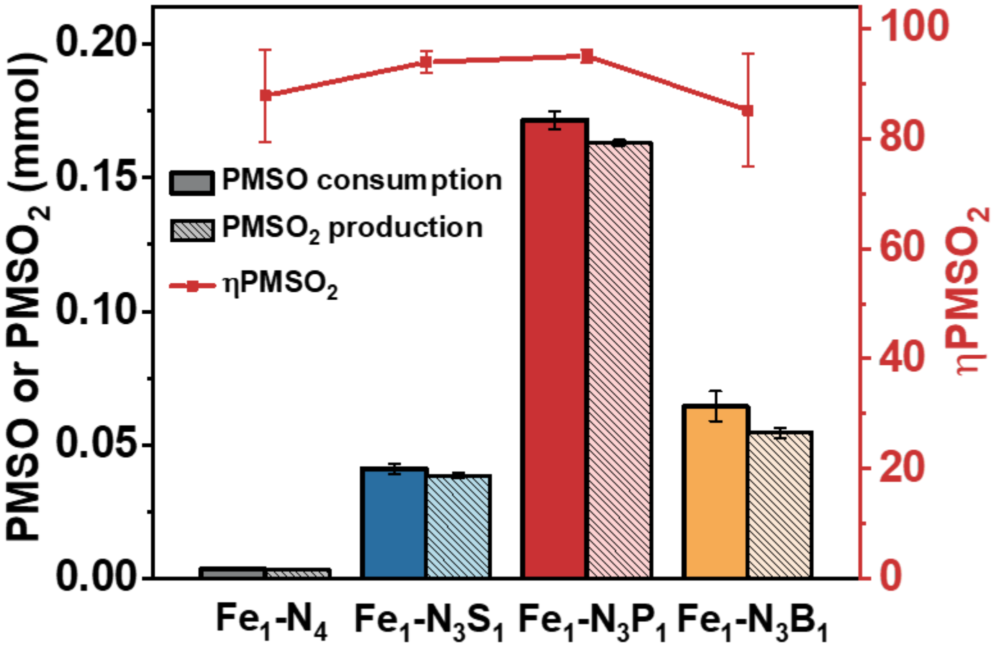

Figure S13. (A) Fe^{IV}=O concentration in Fe_1-N_4, Fe_1-N_3B_1, Fe_1-N_3P_1, and Fe_1-N_3B_1 systems activated PMS systems as a function. (Conditions: PMS = 0.5 mM, catalysts = 0.5 g L^{-1}).

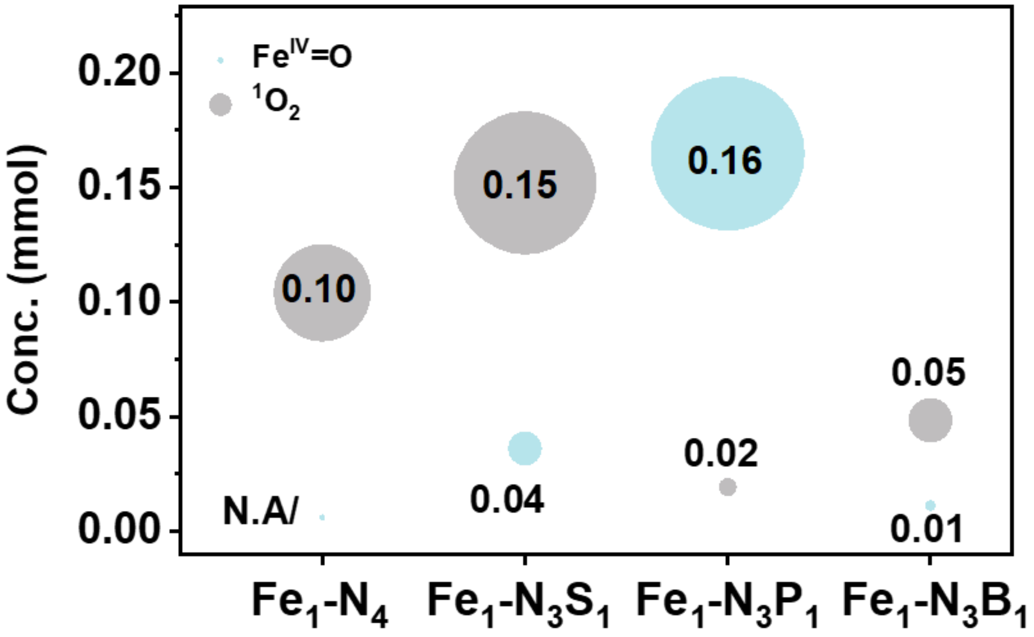

Figure S14. Quantitative experiments of Fe^{IV}=O in Fe_1-N_4, Fe_1-N_3B_1, Fe_1-N_3P_1, and Fe_1-N_3B_1 systems within 10 minutes.

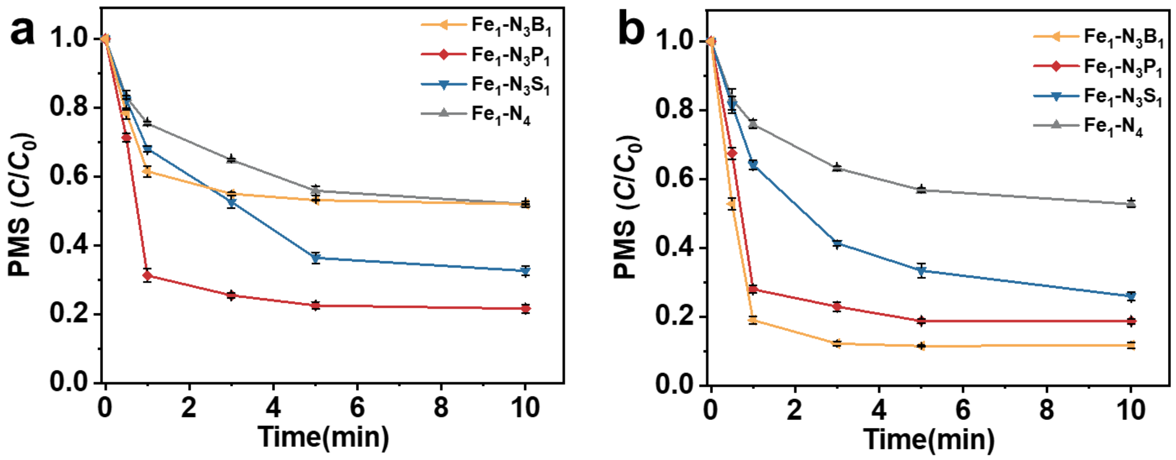

Figure S15. The consumption of PMS during the reaction process of (a) PMS+catalyst system and (b) PMS + catalyst + 4-CP system. (Conditions: 4-CP =0 or 0.1 mM, PMS = 0.5 mM, catalysts = 0.5 g L^{-1}).

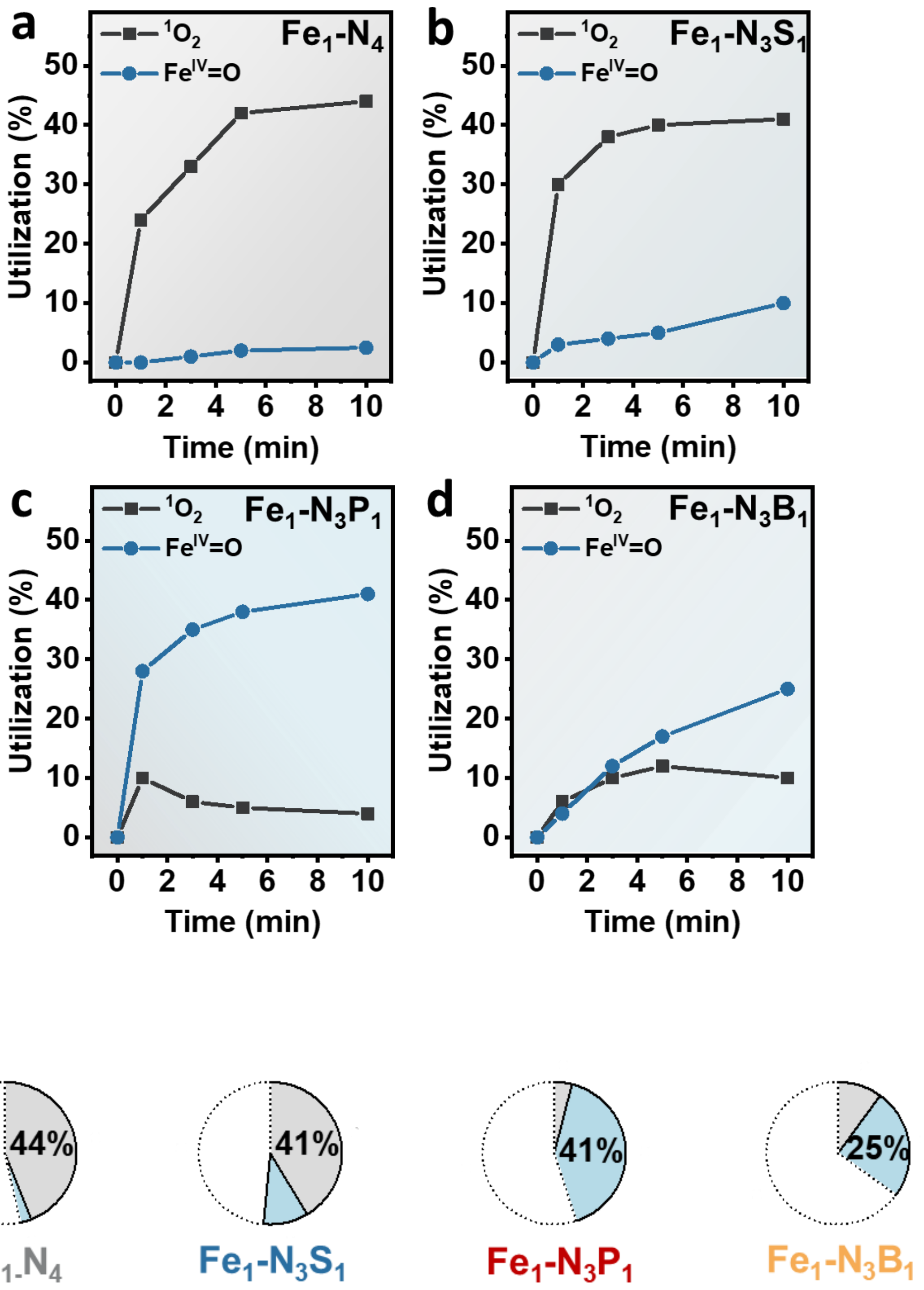

Figure S16. PMS utilization rate experiment of reaction time (respectively for $^1O_2$ and Fe^{IV}=O) of (a) Fe_1-N_4, (b) Fe_1-N_3S_1, (c) Fe_1-N_3P_1, and (d) Fe_1-N_3B_1.

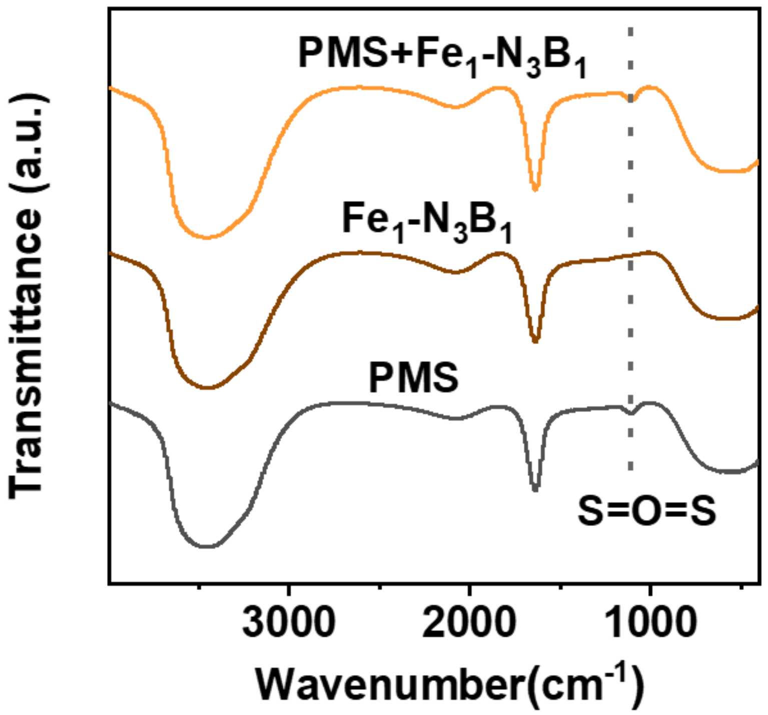

Figure S17. FTIR spectra of the Fe_1-N_3B_1 during the reaction process.

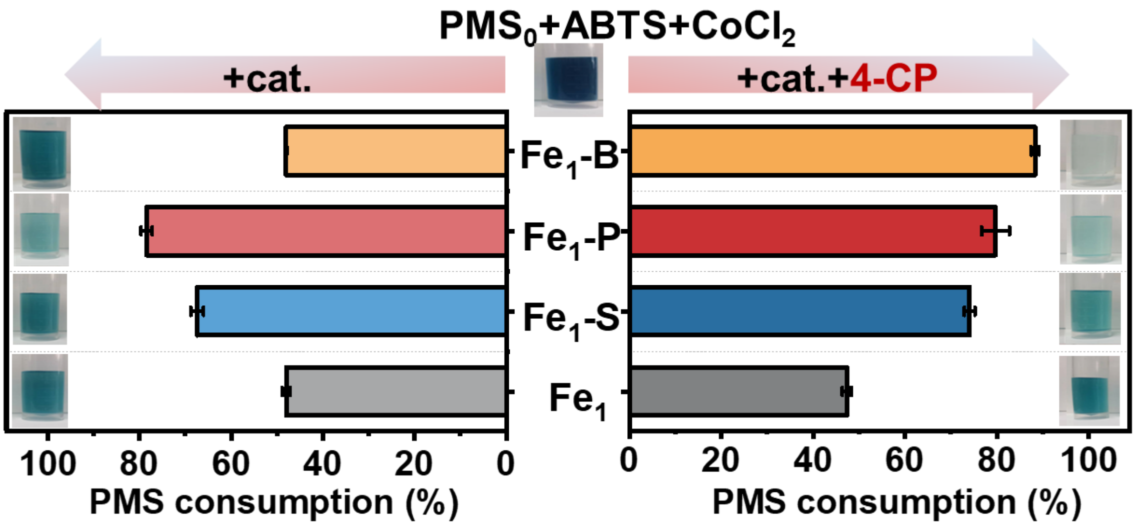

Figure S18. PMS consumption with or without 4-CP (the pictures show the remaining concentration of PMS after 10 minutes).

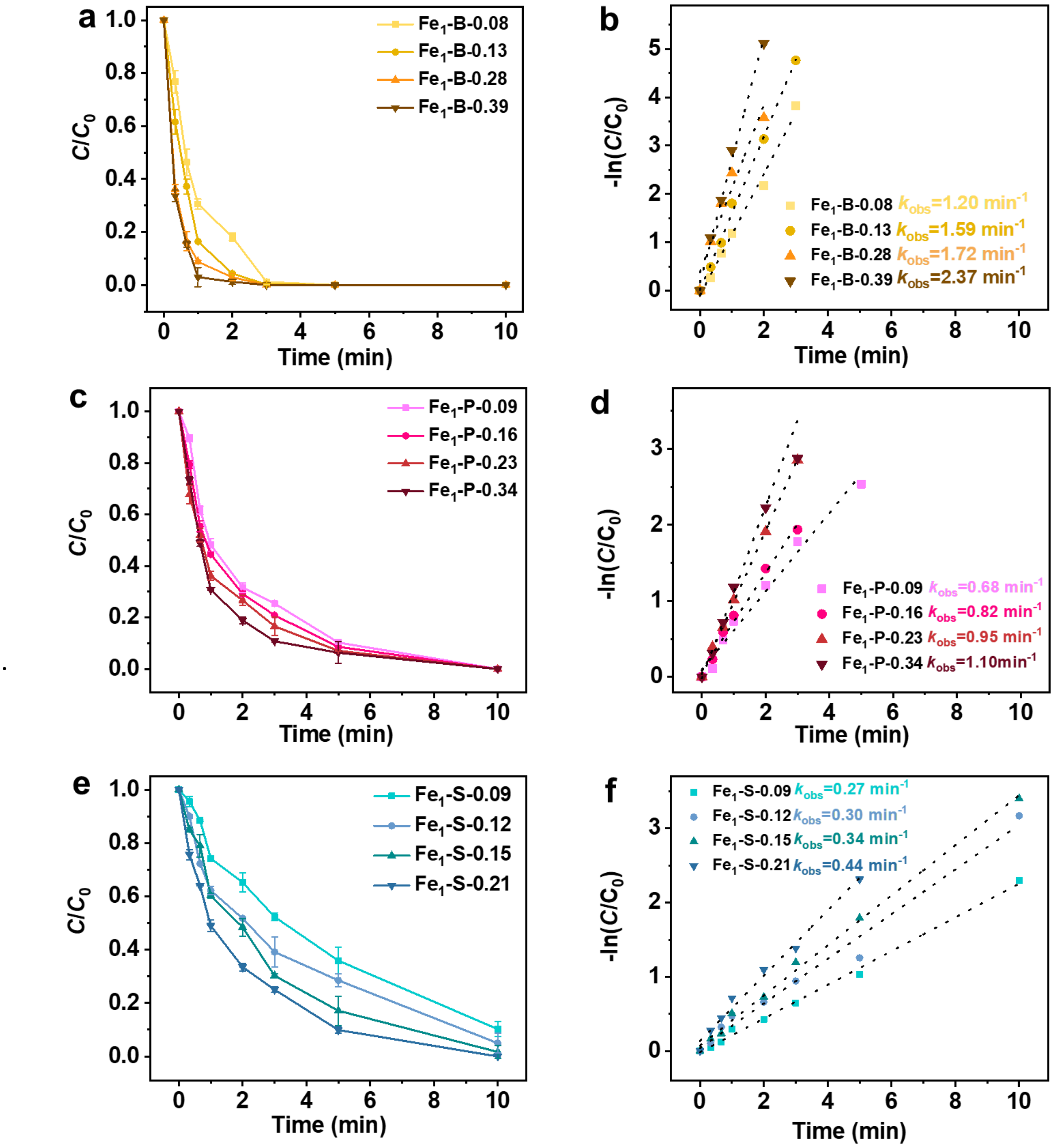

Figure S19. Degradation curves and kinetic fitting of 4-CP degradation by Fe_1-X with different doping ratios. (Conditions: 4-CP = 0.1 mM, PMS = 0.5 mM, catalysts = 0.5 g L^{-1}).

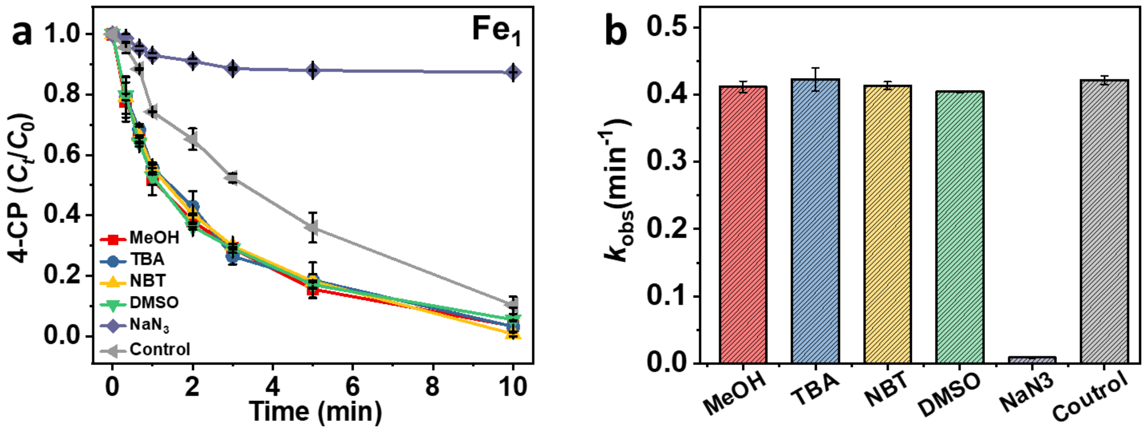

Figure S20. (a) 4-CP degradation efficiency after the addition of quenchers, (b) k_{obs} of quenching tests by Fe_1 activated PMS. (Conditions: 4-CP = 0.1 mM, PMS = 0.5 mM, catalysts = 0.5 g L^{-1}, MeOH = TBA = 500 mM, NBT = PMSO = 2.5 mM, NaN_3 = 5 mM).

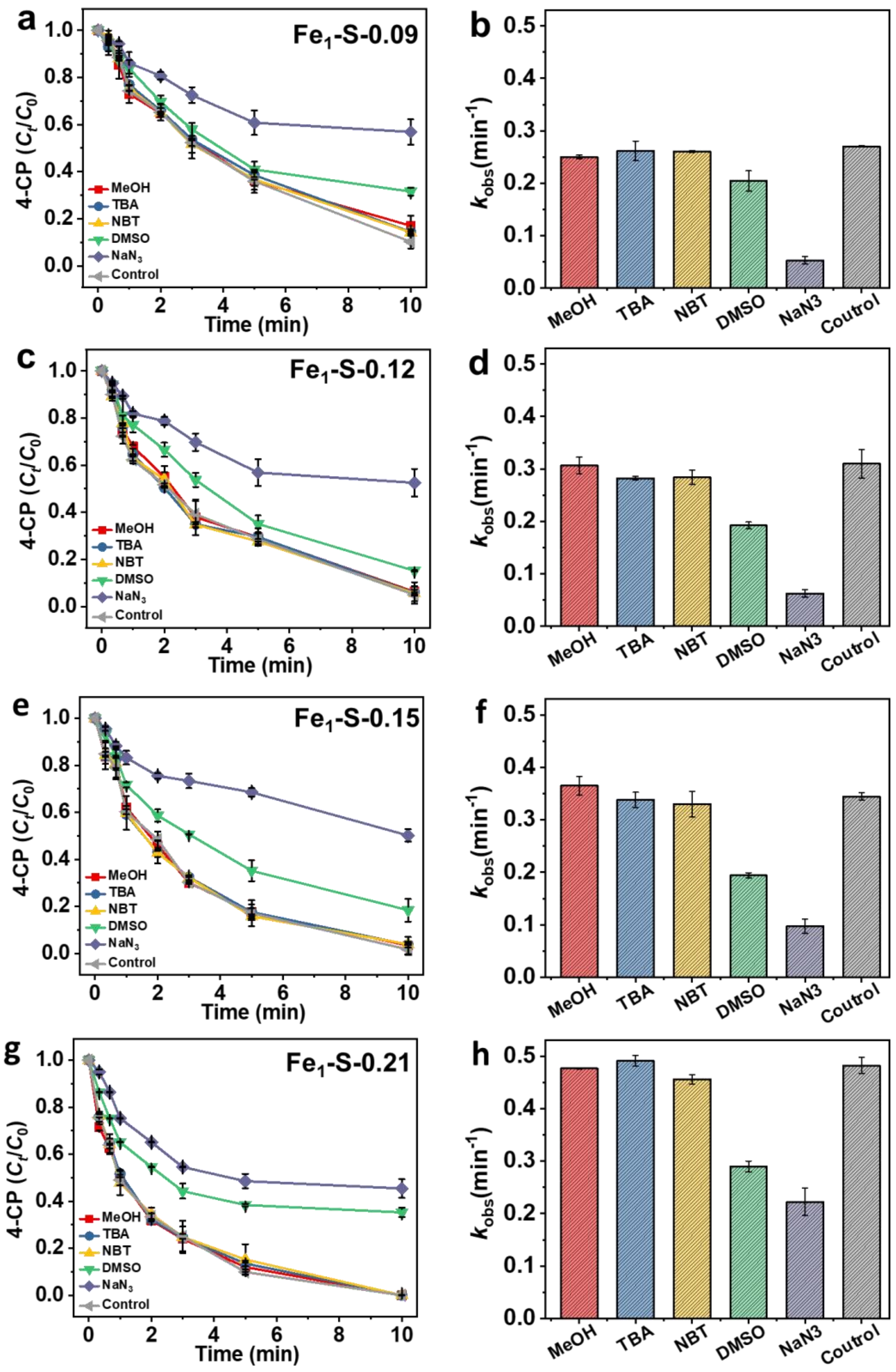

Figure S21. 4-CP degradation efficiency after the addition of quenchers and k_{obs} of quenching tests by Fe_1-S with different doping ratios, activated PMS. (Conditions: 4-CP = 0.1 mM, PMS = 0.5 mM, catalysts = 0.5 g L^{-1}, MeOH = TBA = 500 mM, NBT = PMSO = 2.5 mM, NaN_3 = 5 mM).

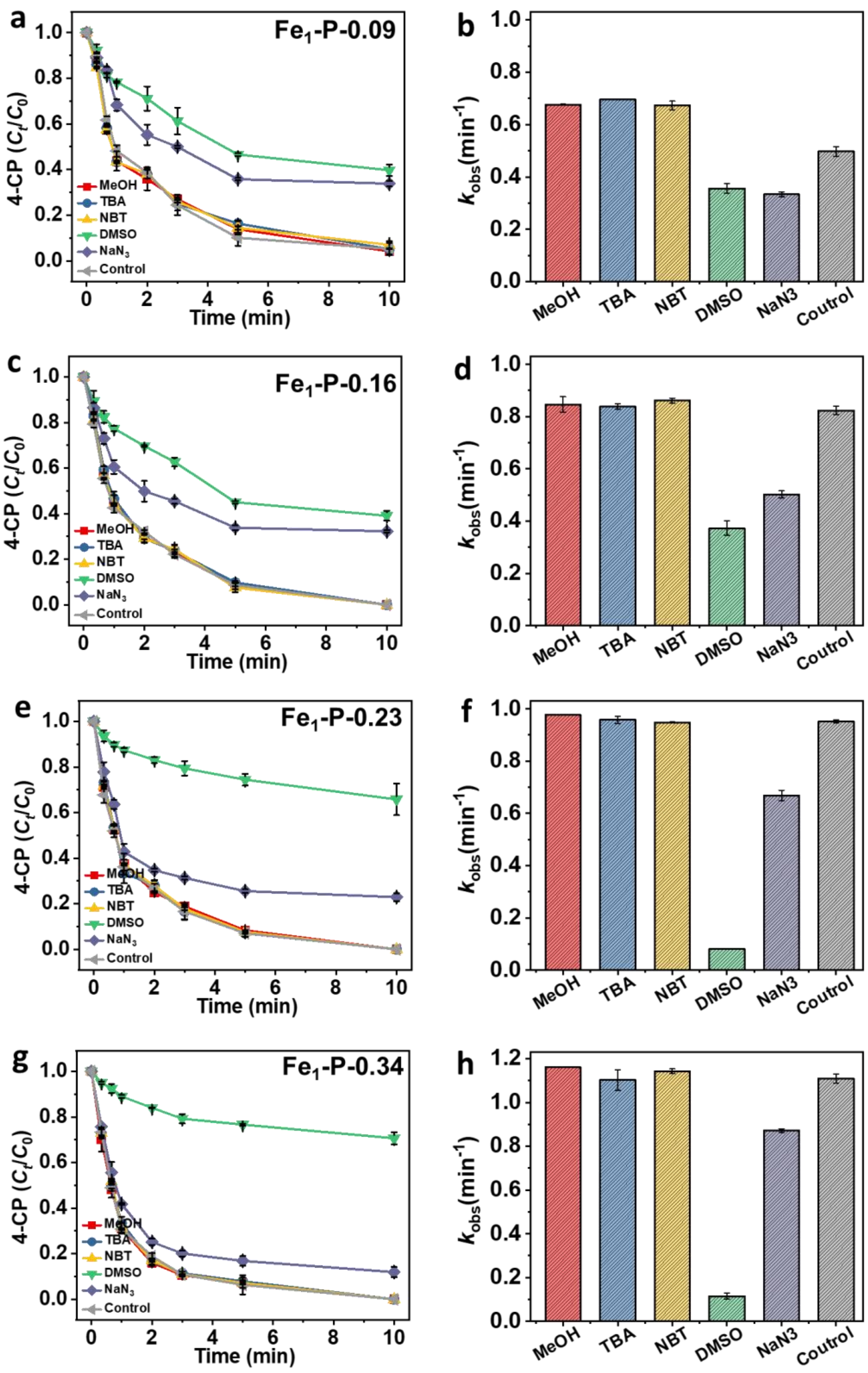

Figure S22. 4-CP degradation efficiency after the addition of quenchers and k_{obs} of quenching tests by Fe_1-P with different doping ratios of activated PMS. (Conditions: 4-CP = 0.1 mM, PMS = 0.5 mM, catalysts = 0.5 g L^{-1}, MeOH = TBA = 500 mM, NBT = PMSO = 2.5 mM, NaN_3 = 5 mM).

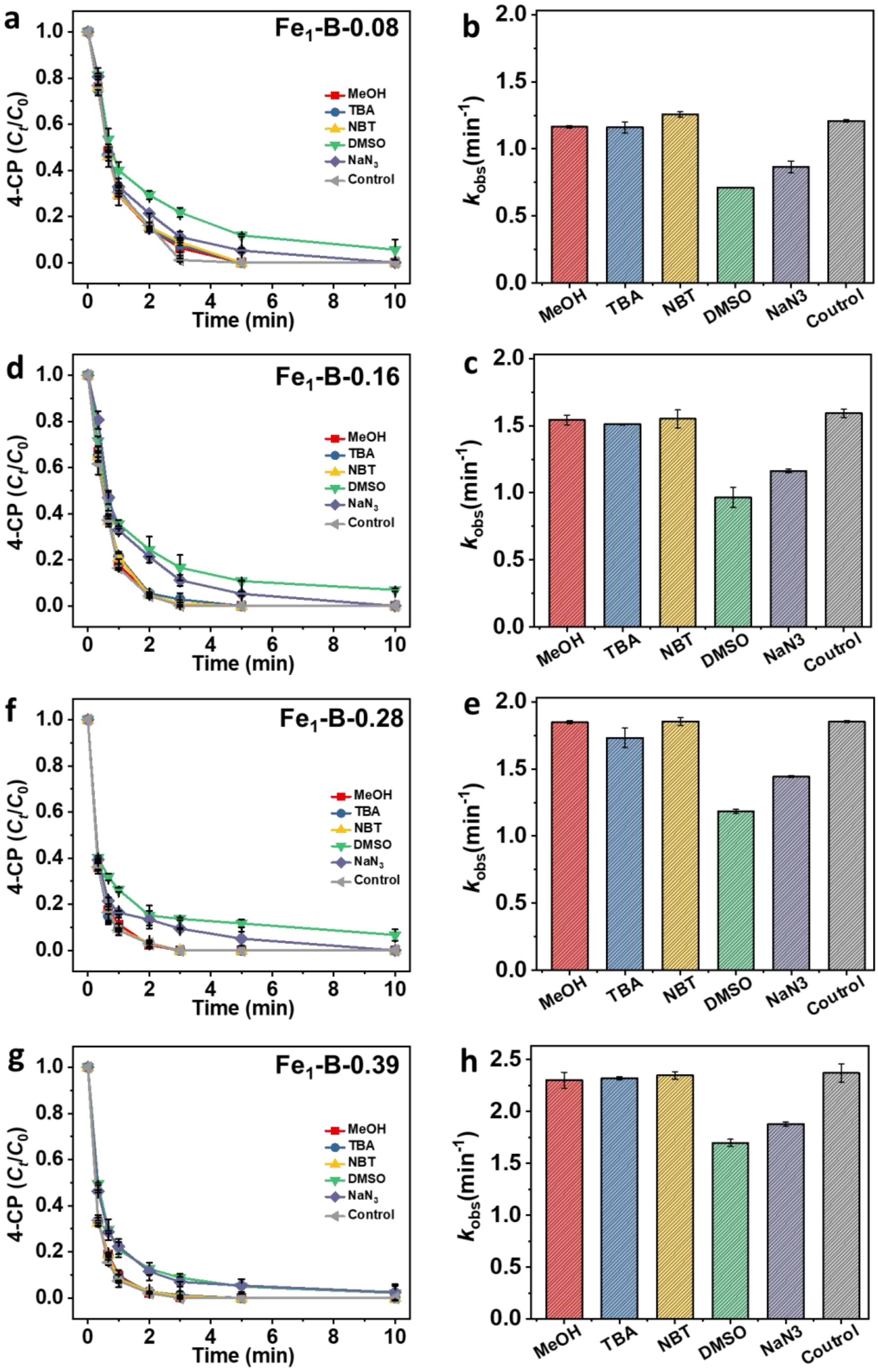

Figure S23. 4-CP degradation efficiency after the addition of quenchers and k_{obs} of quenching tests by Fe_1-B with different doping ratios of activated PMS. (Conditions: 4-CP = 0.1 mM, PMS = 0.5 mM, catalysts = 0.5 g L^{-1}, MeOH = TBA = 500 mM, NBT = PMSO = 2.5 mM, NaN_3 = 5 mM).

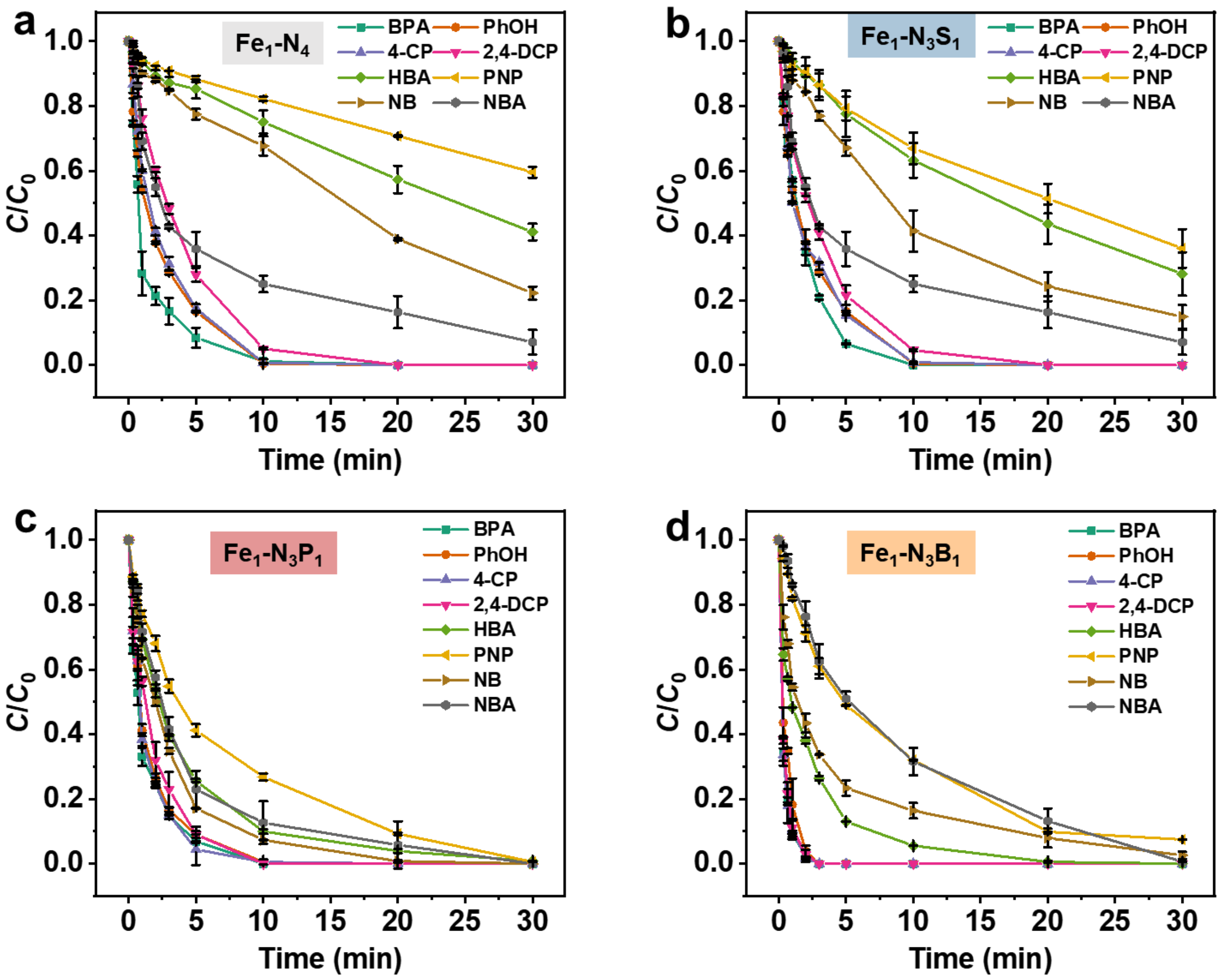

Figure S24. The degradation efficiency of various aromatic pollutants by Fe_1-N_4, Fe_1-N_3B_1, Fe_1-N_3P_1, and Fe_1-N_3B_1 activated PMS systems. (Conditions: initial pH = 5.6, T = 25 ± 2°C, pollutants = 0.1 mM, PMS = 0.5 mM, catalysts = 0.5 g L^{-1}).

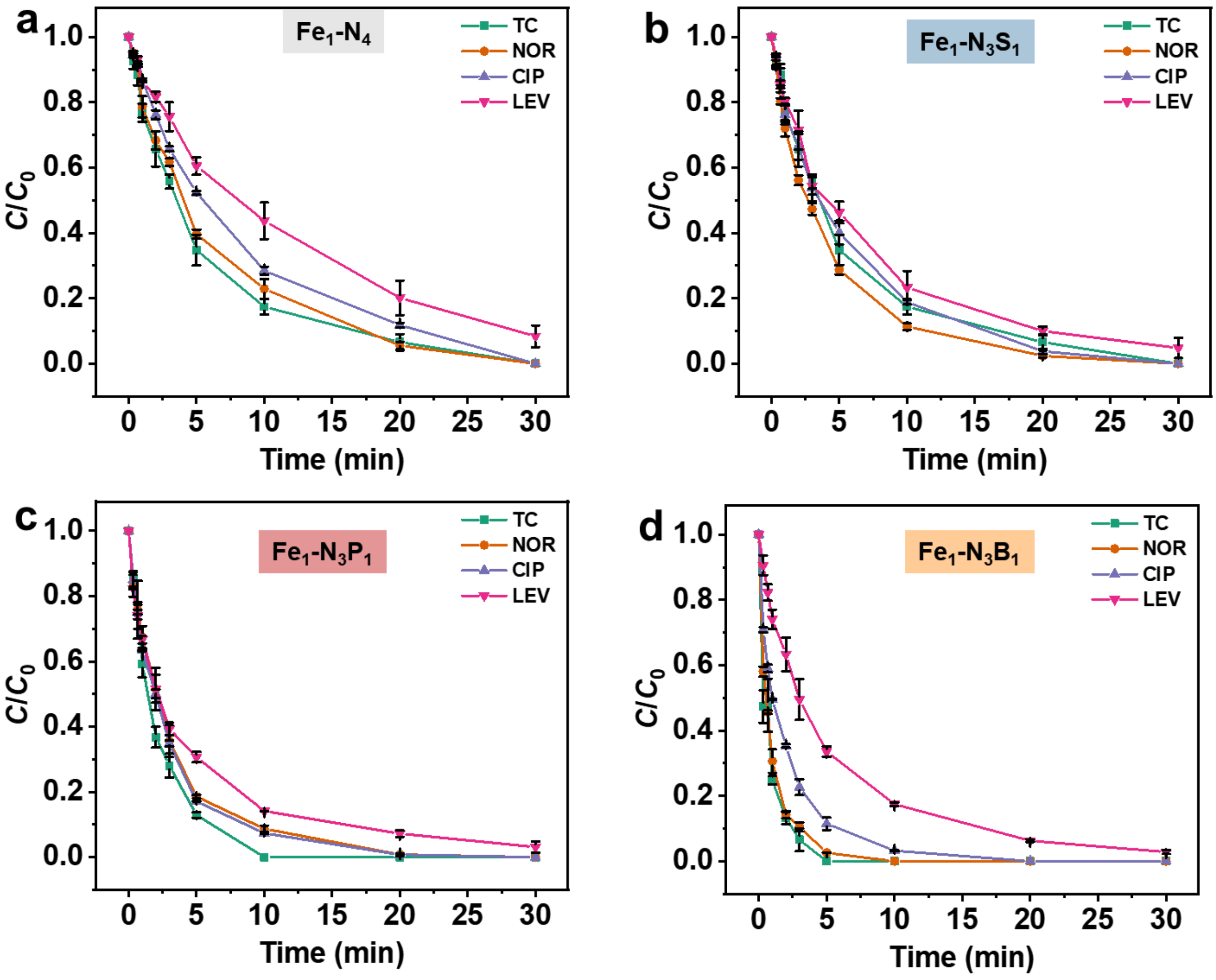

Figure S25. The degradation efficiency of various antibiotics by Fe_1-N_4, Fe_1-N_3B_1, Fe_1-N_3P_1, and Fe_1-N_3B_1 activated PMS systems. (Conditions: initial pH = 5.6, T = 25 ± 2°C, pollutants = 0.1 mM, PMS = 0.5 mM, catalysts = 0.5 g L^{-1}).

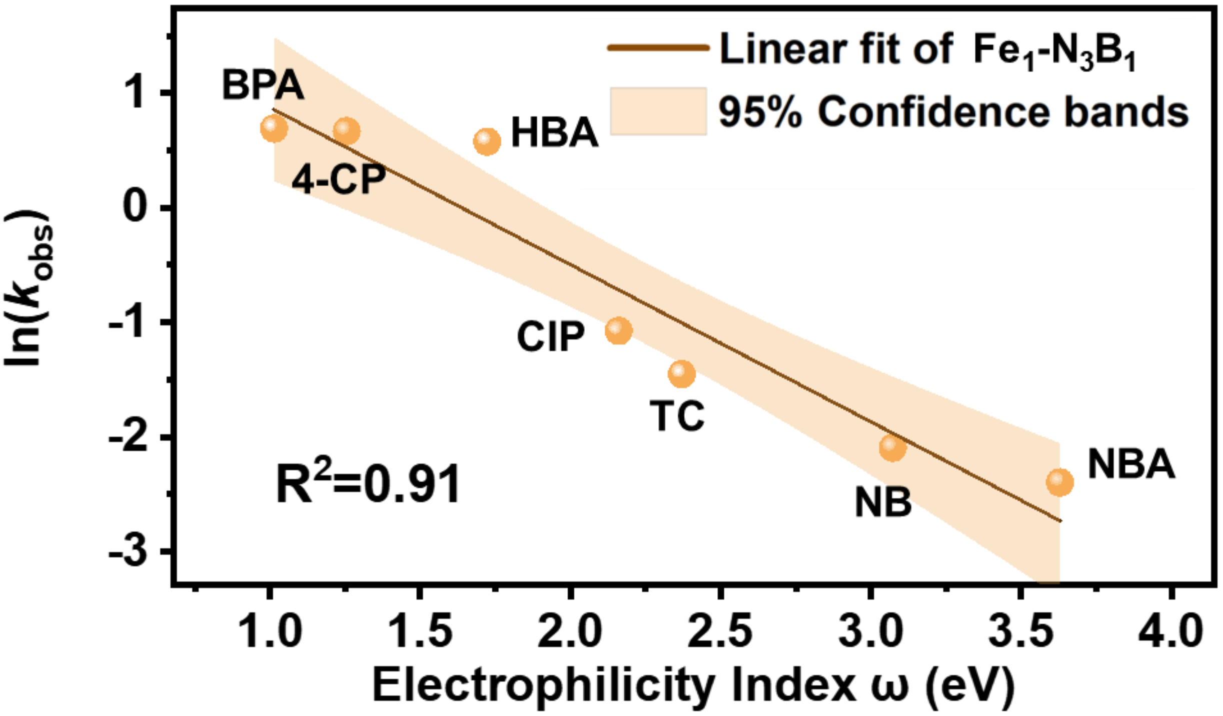

Figure S26. Linearity between the electrophilic indexes of different pollutants and their ln(k_{obs}) values in Fe_1-N_3B_1/PMS systems.

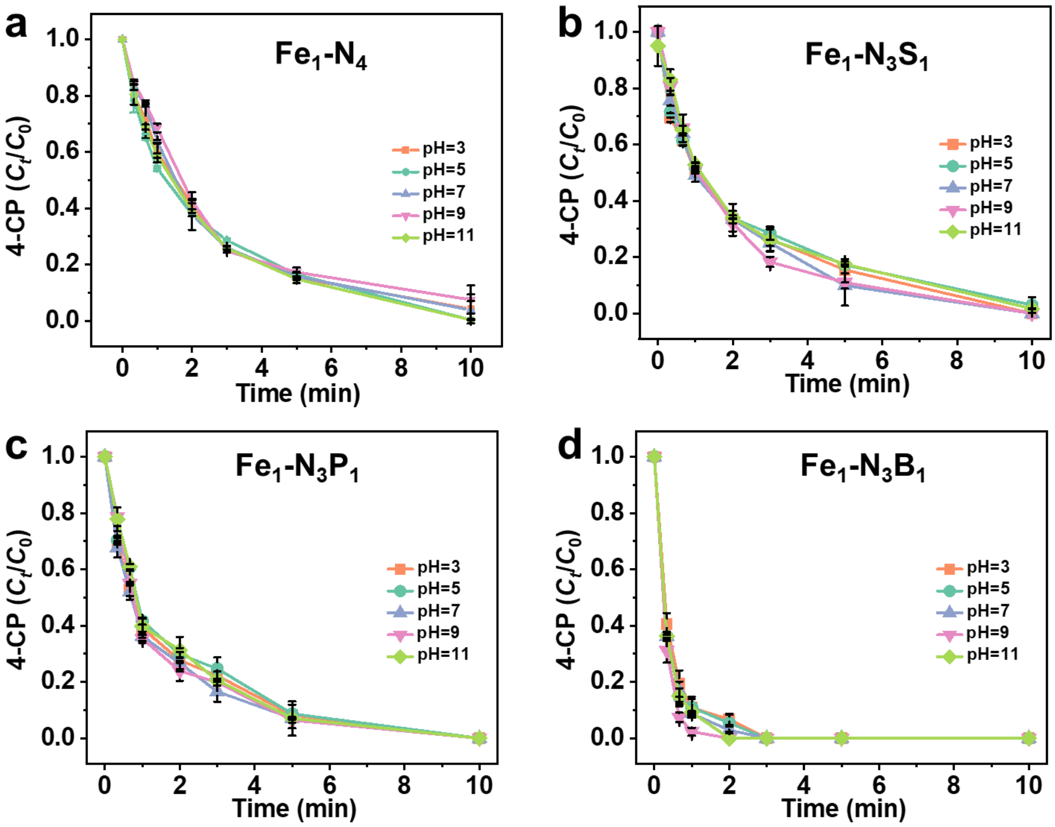

Figure S27. The 4-CP degradation efficiency in (a) Fe_1-N_4, (b) Fe_1-N_3S_1, (c) Fe_1-N_3P_1, and (d) Fe_1-N_3B_1 activated PMS systems at different initial pH. (Conditions: 4-CP = 0.1 mM, PMS = 0.5 mM, catalysts = 0.5 g L^{-1}).

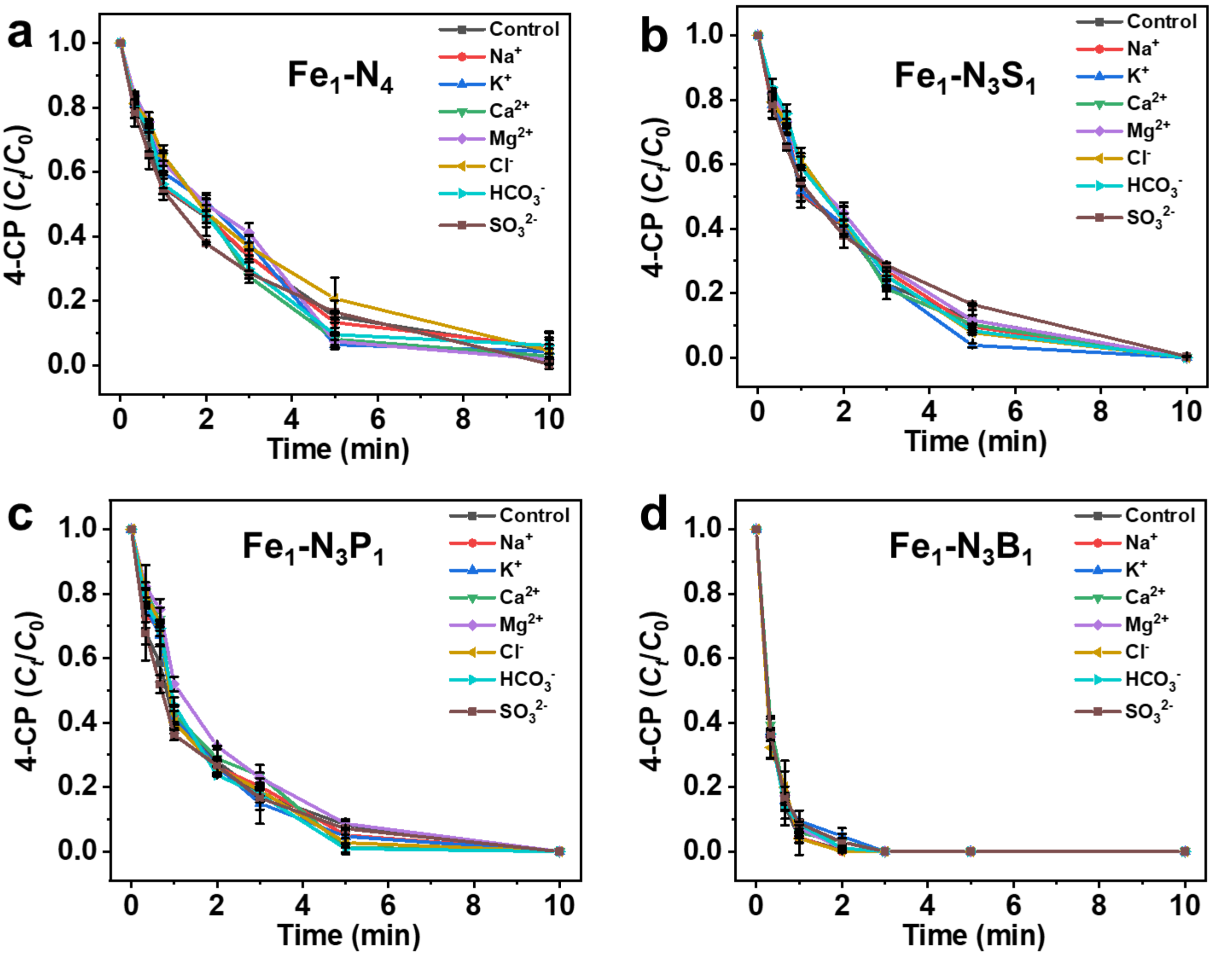

Figure S28. The 4-CP degradation efficiency in (a) Fe_1-N_4, (b) Fe_1-N_3S_1, (c) Fe_1-N_3P_1, and (d) Fe_1-N_3B_1 activated PMS systems with background ions. (Conditions: 4-CP = 0.1 mM, PMS = 0.5 mM, catalysts = 0.5 g L^{-1}).

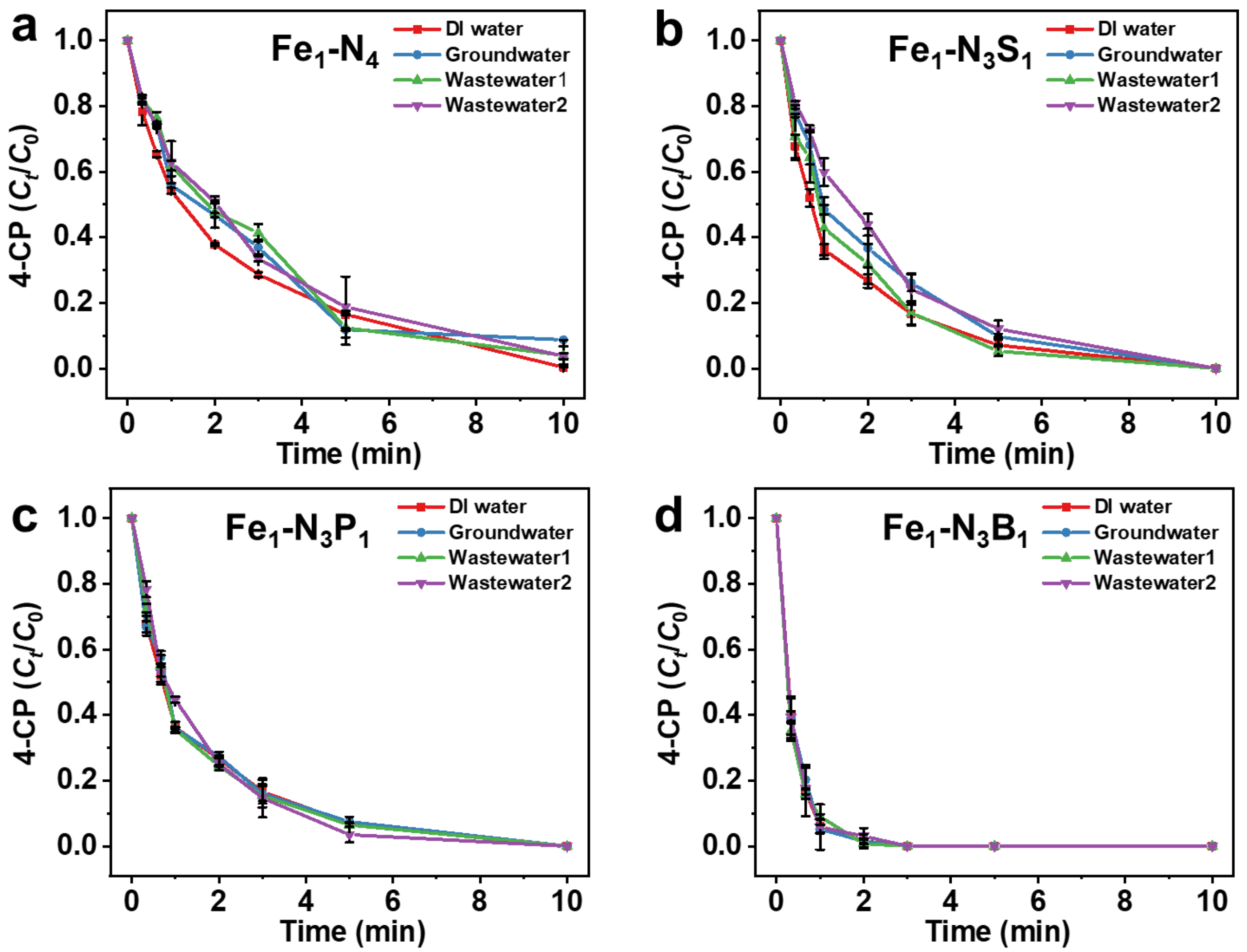

Figure S29. The 4-CP degradation efficiency in (a) Fe_1-N_4, (b) Fe_1-N_3S_1, (c) Fe_1-N_3P_1, and (d) Fe_1-N_3B_1 activated PMS systems at different waters. (Conditions: 4-CP = 0.1 mM, PMS = 0.5 mM, catalysts = 0.5 g L^{-1}).

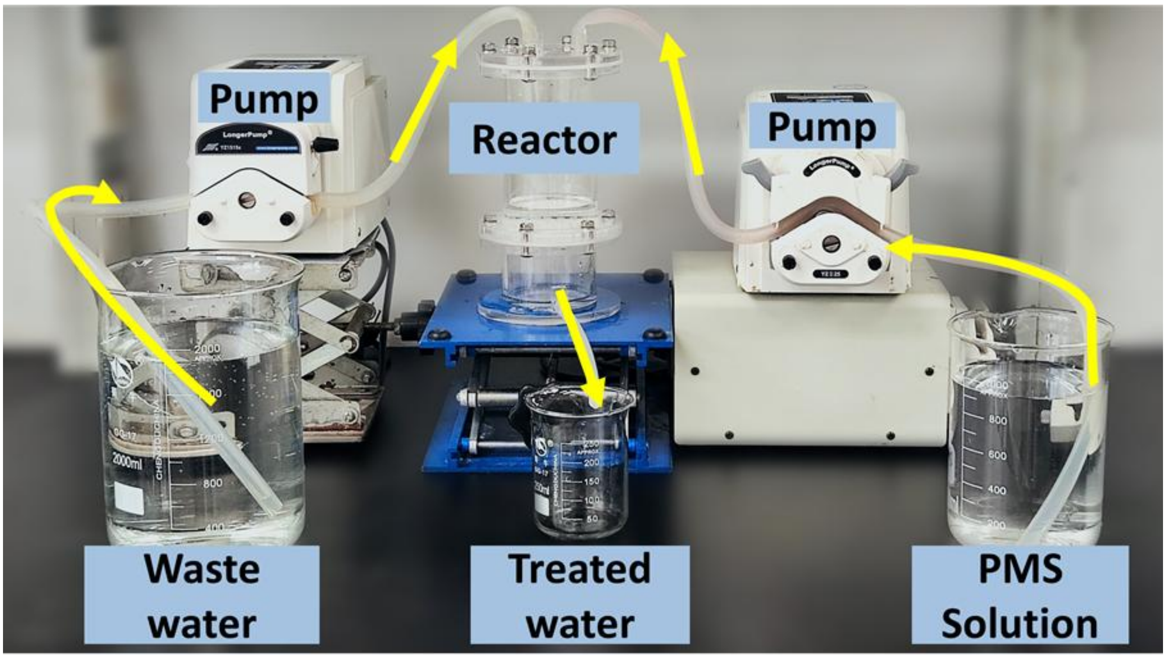

Figure S30. The physical diagram of the continuous flow membrane reaction device. In this test, the dosage of Fe_1-N_4, Fe_1-N_3B_1, Fe_1-N_3P_1, and Fe_1-N_3B_1 was 200 mg, and the flow rate was 1 mL/min. Samples were taken at 30-minute intervals for a total of 10 hours.

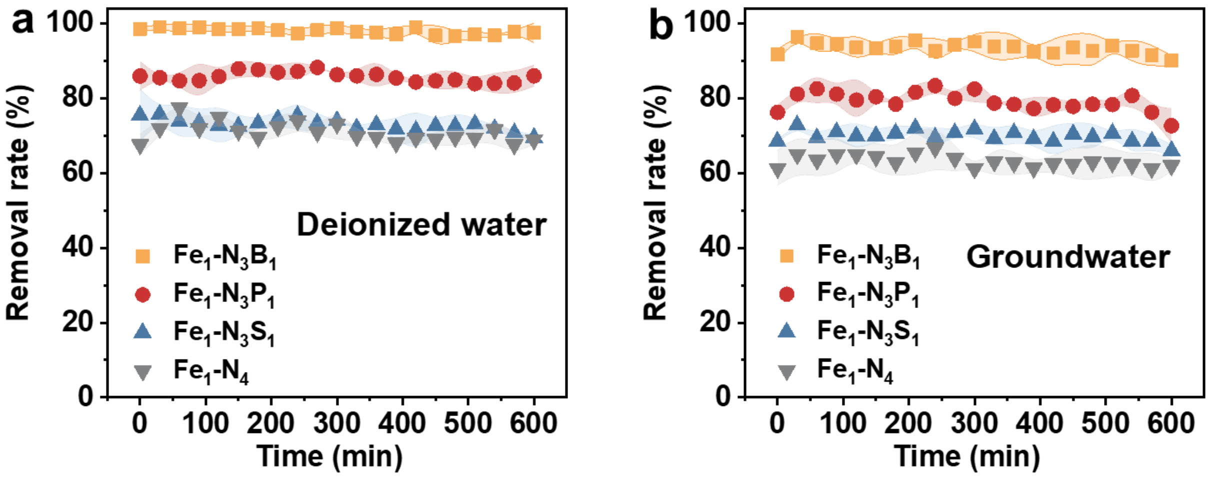

Figure S31. The removal rate of 4-CP during the 600-minute device experiment in deionized water and groundwater. (Conditions: 4-CP = 0.1 mM, PMS = 1 mM).

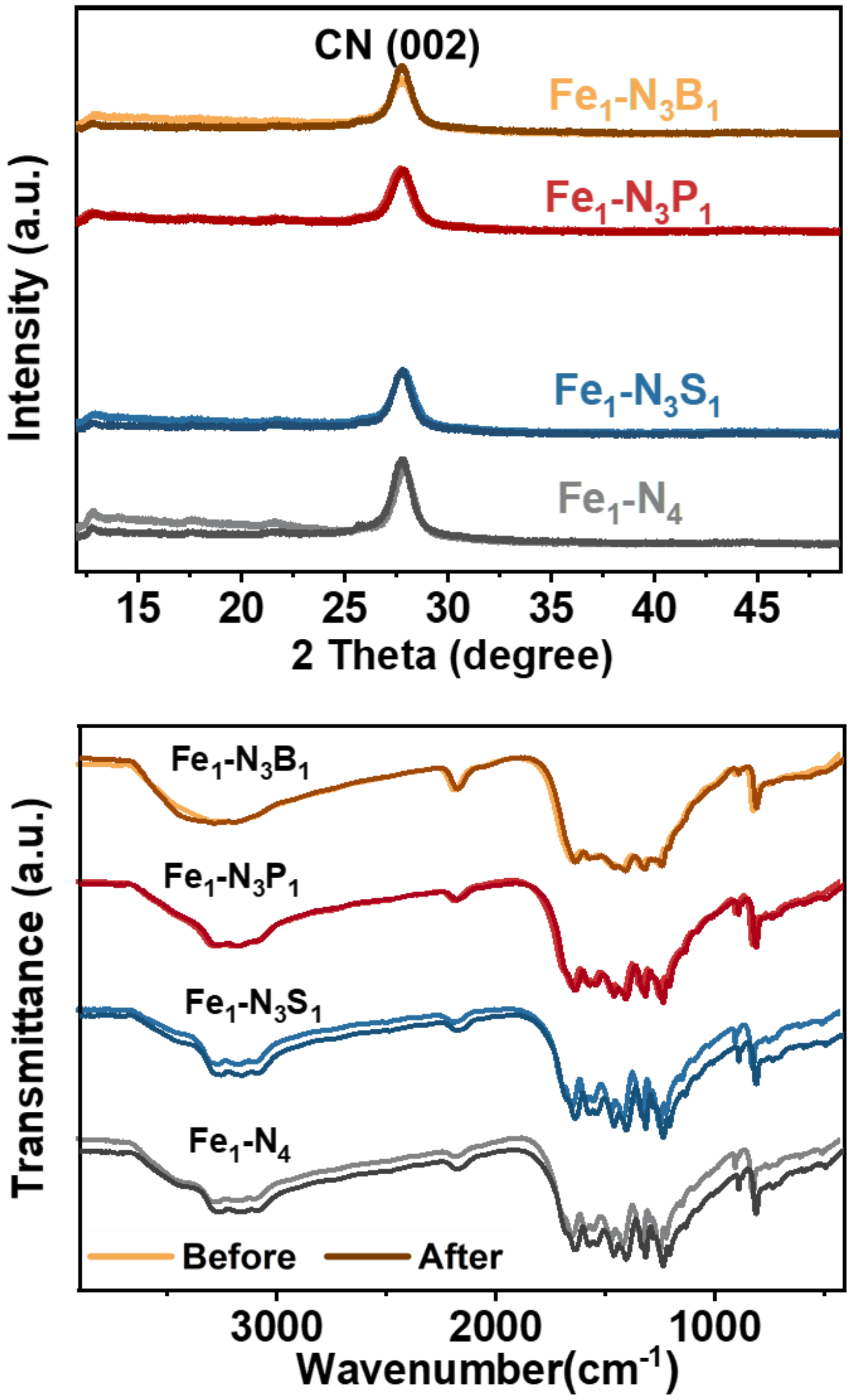

Figure S32. XRD patterns and FTIR spectra of the Fe_1-N_4, Fe_1-N_3B_1, Fe_1-N_3P_1, and Fe_1-N_3B_1 before and after the reaction.

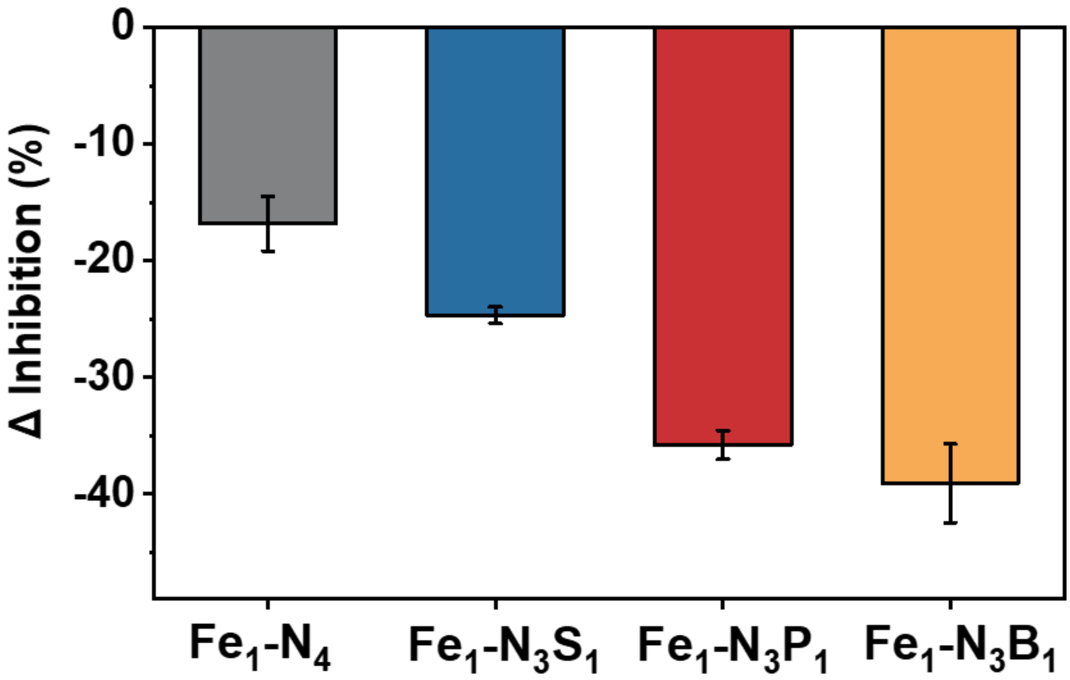

Figure S33. The change of the inhibition rate in the solution of luminescent bacteria solution and the solution after the reaction. (Conditions: 4-CP = 0.1 mM, PMS = 0.5 mM, catalysts = 0.5 g L^{-1}).

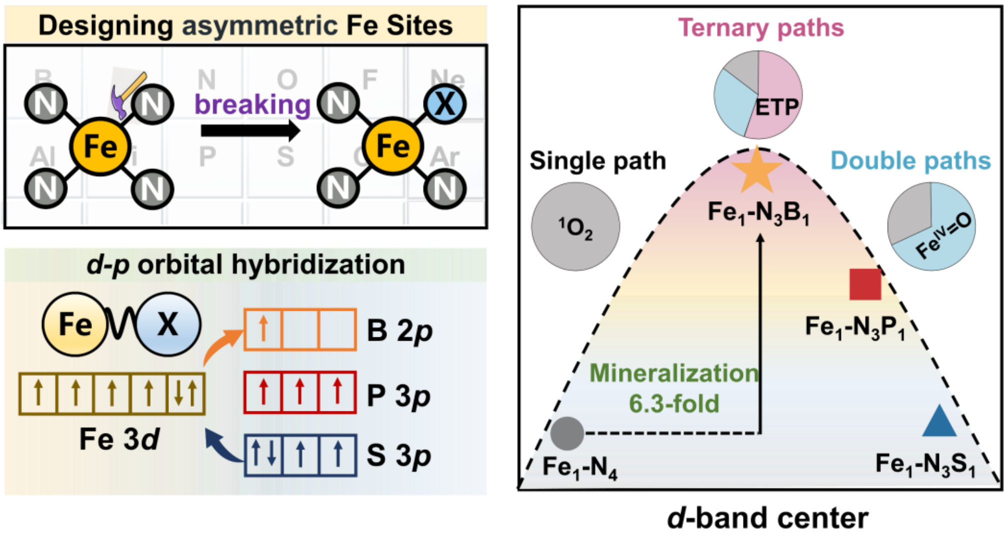

Figure S34. Summary of the results.

3. Supplementary Tables

Table S1. Fe and *p*-block element content of samples.

Samples	Fe content[wt. %][a]	X content[wt. %][a]		X/Fe molar ratio
Fe_1	11.38±0.48	\	\	\
Fe_1-S-0.09	11.99±0.98		0.60±0.01	0.088±0.009
Fe_1-S-0.12	11.11±0.13	S	0.80±0.04	0.127±0.008
Fe_1-S-0.15	10.50±0.02		0.90±0.0	0.149±0.002
Fe_1-S-0.21	10.19±0.14		1.22±0.02	0.209±0.002
Fe_1-P-0.09	9.69±0.26		0.52±0.01	0.096±0.001
Fe_1-P-0.16	12.15±0.56	P	1.10±0.03	0.165±0.013
Fe_1-P-0.24	9.83±0.42		1.29±0.01	0.237±0.007
Fe_1-P-0.36	10.95±0.02		2.19±0.03	0.362+0.005
Fe_1-B-0.08	11.83±1.21		0.19±0.01	0.086±0.012
Fe_1-B-0.18	11.73±0.53	B	0.41±0.01	0.183±0.005
Fe_1-B-0.28	10.56±0.26		0.57±0.04	0.279±0.014
Fe_1-B-0.39	12.03±0.10		0.90±0.02	0.389±0.006

Table S2. EXAFS fitting parameters at the Fe K-edge for various samples.

Sample	Shell	ᵃN	ᵇR(Å)	ᶜσ²(Å²)	R factor
Fe_1-N_4	Fe−N18.1	2.37	2.09	0.01	0.011
	Fe−N22.1	1.64	2.39	0.01	
Fe_1-N_3S_1	Fe−N18.1	2.45	2.18	0.006	0.009
	Fe−S8.1	1.40	2.17	0.006	
Fe_1-N_3P_1	Fe−N18.1	2.83	2.18	0.01	0.006
	Fe−P8.1	0.97	2.15	0.01	
Fe_1-N_3B_1	Fe−N18.1	1.09	2.07	0.008	0.012
	Fe−N27.1	1.89	2.86	0.008	
	Fe−B8.1	1.19	2.23	0.008	

[a]N: coordination numbers; [b]R: bond distance; [c]σ^2: change in the Debye-Waller factors relative to that of the reference compound. [e]R factor: goodness of fit.

Table S3. The variation in the charge of elements in the Fe_1-N_4 and Fe_1-N_3X_1.

Catalyst	Element	Charge variation
Fe_1-N_4	N4	1.03
	N8	0.97
	N18	1.01
	N22	0.99
	Fe1	−1.06
Fe_1-N_3S_1	N17	1.08
	N21	0.99
	N26	0.94
	S1	−0.22
	Fe1	−0.98
Fe_1-N_3P_1	N17	1.02
	N21	0.91
	N26	0.94
	P1	−0.28
	Fe1	−0.91
Fe_1-N_3B_1	N4	0.90
	N17	0.88
	N21	0.91
	B1	−0.66
	Fe1	−0.85

Table S4. A comparison of TOC in the recently reported Fenton-like catalysts for the degradation of 4-CP.

	path	Catalyst	PMS (mM)	pollutant	TOC	Time (min)	Ref.
Single path	$^1O_2$	P-Mn-TPs	3.2	PhOH	62.9%	60	[4]
	$^1O_2$	$BiVO_4@Bi_2S_3$	3	TC	93.8%	35	[5]
	ETP	FeCo-DAC	0.4	/	75.0%	15	[6]
	ETP	FeSA-N-C	2.6	BPA	56.9%	15	[7]
	Fe^{IV}=O	Fe-N-C c	0.65	SMX	52.1%	180	[8]
	ETP	e/rGO	0.35	phenolic	51.1%	120	[9]
	ETP	Cu-Co_3O_4/OVs	0.3	OFX	35.4%	12	[10]
	$^1O_2$	Fe_1	0.5	4-CP	21.3%	10	/
Double paths	$^1O_2+Fe^{IV}$=O	SFC	0.6	BPA	30.0%	90	[11]
	$^1O_2+Fe^{IV}$=O	$Co_{SAs\text{-}NCs}$/CN	3.9	NFX	76.4%	60	[12]
	$^1O_2$+ETP	$NiCo_2O_4$	0.32	TC	36.7%	30	[13]
	$^1O_2$+ETP	Mn-CN	2.6	diclofenac	80.0%	60	[14]
	$^1O_2$+ETP	Fe-SA	1	PhOH	55.1%	30	[15]
	$^1O_2+\cdot OH$	MIL-101(Fe)	1	SMX	46.2%	30	[16]
	$O_2^{\cdot-}+{}^1O_2$	Fe/Mn@NBC	3	BPA	63.09%	20	[17]
	$^1O_2+Fe^{IV}$=O	Fe_1-S	0.5	4-CP	34.6%	10	/
	$^1O_2+Fe^{IV}$=O	Fe_1-P	0.5	4-CP	45.1%	10	/
Ternary paths	$^1O_2+SO_4^{\cdot-}+\cdot OH$	Fe_3O_4/S-WO_3	1	Chloroxylenol	45.4%	10	[18]
	$\cdot OH+SO_4^{\cdot-}+{}^1O_2$	CoNC/NHCNTs	1.95	TC	89.07 %	30	[19]
	$^1O_2+Fe^{IV}$=O+ETP	Fe_1-B	0.5	4-CP	85.2%	10	/
	ETP+$O_2^{\cdot-}+{}^1O_2$	110-Cu	1	BPA	94.55%	30	[20]
	$\cdot OH+SO_4^{\cdot-}+{}^1O_2$	FeCu/OPB-1	0.65	BPA	70%	90	[21]
Radical pathway	$^1O_2+\cdot OH$	Fe/CNT	5	SMX	98.4%	30	[22]
	$SO_4^{\cdot-}$	FeSeS@C	1.5	diatrizoic acid	76.8%	60	[23]
	$\cdot OH+SO_4^{\cdot-}$	Cu_1–O_v/TiO_2	0.3	BPA	65%	8	[24]

Catalyst	Loading (wt%)	PMS (mM)	pollutant	k_{obs} (min^{-1})	Ref.
$Fe-P_1N_3$	0.77	0.24	BPA	0.397	[25]
Fe/N-SAC	1.15	0.81	4-CP	0.3	[26]
Mn-C-N	0.01	0.65	BPA	0.21	[14]
$Cu-In_2O_3/O_v$	1.00	1.00	TC	0.265	[27]
$Fe-N_3C_1$	0.75	0.30	SIZ	0.124	[28]
10-SAFe-CN	3.77	0.65	o-phenylphenol	0.161	[29]
Fe-Co DAC	2.30	0.40	BPA	0.23	[30]
Fe-NC-MP-5	1.16	1.00	BPA	0.2	[31]
$Mn-N_5-C$	4.07	4.00	4-CP	0.4	[32]
$Cu-N_4/C-B$	1.05	0.65	BPA	0.560	[33]
Fe-SAC	1.75	0.50	4-CP	0.8	[34]
$D-FeN_4-C$	0.69	0.20	SIZ	0.616	[35]
SACu@NBC	3.14	1.31	BPA	0.006	[36]
Fe-N-C	3.10	0.50	4-CP	0.39	[37]
$B-FeN_4CA$	1.66	0.20	SMX	0.485	[38]
BvBN/Fe	0.19	0.25	BPA	0.492	[39]
CoNBC600	0.23	1.97	APAP	0.46	[40]
Fe_1-N_4	11.38	0.50	4-CP	0.41	This work
$Fe_1-N_3S_1$	10.19	0.50	4-CP	0.44	This work
$Fe_1-N_3P_1$	9.83	0.50	4-CP	0.95	This work
$Fe_1-N_3B_1$	10.56	0.50	4-CP	1.80	This work

Table S5. A comparison of k_{obs} and loading in the recently reported catalysts.

Table S6. The *d*-band of Fe and the PMS* in the recently reported catalysts.

Catalyst	*d*-band	PMS*	Ref.
SA-Fe-N_4	−1.05	−2.96	[41]
Fe-P_1N_3	−0.84	−0.66	[25]
Fe-N_4	−0.91	−1.35	[25]
Fe_2P	−1.35	−1.05	[28]
Fe−N_4	−1.20	−2.06	[28]
Fe−N_3C_1	−1.00	−2.25	[28]
Fe−N_2C_2	−0.69	−2.38	[28]
Fe−N_3C_1	−1.27	−2.37	[42]
Fe-N_4	−0.39	−2.20	[42]
FeN_4−C	−1.11	−2.19	[35]
D−FeN_4−C	−0.32	−2.75	[35]
B-FeN_4CA	−1.22	−2.87	[38]
P-FeN_4CA	−1.47	−2.59	[38]
FeN_4CA	−1.35	−2.71	[38]
Fe_1-N_4	−1.27	−3.11	This work
Fe_1-N_3S_1	−0.89	−3.40	This work
Fe_1-N_3P_1	−0.96	−3.38	This work
Fe_1-N_3B_1	−1.01	−3.25	This work

Table S7. Relative parameters of different pollutants.

Organic matter	E_{HOMO} (eV)	E_{LUMO} (eV)	ΔE=E_{LUMO}-E_{HOMO}	Chemical potential (μ)	Electrophilicity index (ω)
BPA	-5.96	-0.62	5.34	-3.29	1.01
4-CP	-6.44	-0.97	5.47	-3.71	1.25
HBA	-6.86	-1.63	5.23	-4.25	1.72
CIP	-6.57	-2.16	4.41	-4.37	2.16
TC	-6.25	-2.35	3.9	-4.30	2.37
NB	-7.99	-3.04	4.95	-5.52	3.07
NBA	-8.28	-3.50	4.78	-5.89	3.63

Reference [34] for HOMO and LOMO values.

Table S8. The HPLC analysis conditions for different substrates.

Substrates	Flow ($ml\cdot min^{-1}$)	λ (nm)	CH_3OH (%)	Acetonitrile (%)	H_2O (%)	CH_3COOH (%)	HCOOH (%)	H_3PO_4 (%)
BPA	1	278	70		30			
4-CP	1	225		55	45			
2,4-DCP	1	286		20		80		
PhOH	1	270		30	70			
HBA/BQ	1	244		30	70			
NB	1	260	60		40			
NBA	0.8	270		50				50
NOX	1	278		20				80
CIP	1	272	30				70	
LEV	1			20				80
TC	1	357		80				20
PMSO/$PMSO_2$	1	230		20		80		

Table S9. Properties of the water sample.

Parameter	Groundwater	Wastewater 1 (Secondary effluent)	Wastewater 1 (textile wastewater)
UV_{254}	0.06	0.09	0.10
TC (mg C/L)	24.47	36.95	38.45
IC (mg C/L)	1.02	4.52	3.49
TOC (mg C/L)	35.70	47.70	57.90
COD (mg/L)	2.31	18.62	48
Cl^{-} (mg/L)	35.70	47.70	214.78
SO_4^{2-} (mg/L)	55.55	82.37	97.73
NO_3^{-} (mg/L)	62.84	17.63	137.64
PO_4^{3-} (mg/L)	10.56	6.47	1.23
Ca^{2+} (mg/L)	5.26	6.47	15.79
K^{+} (mg/L)	0.50	8.77	12.49
Na^{+} (mg/L)	14.69	31.03	56.48
Mg^{2+} (mg/L)	2.16	1.95	3.45
pH	8.06	7.91	7.7

4. References

[1] X. Hu, M. Zhu, *Environ. Sci. Technol.* **2024**, *58*, 10415-10444.

[2] Z. Li, Z. Guo, X. Wu, X. Jiang, H. Li, J. Xu, K. Yang, D. Lin, *ACS Nano* **2023**, *17*, 22859-22871.

[3] Z. Pan, X. Jiang, X. Feng, Y. Liu, W. Dong, Y. Chen, C. Li, B. Yang, J. Hou, J. Zhang, L. Zhu, D. Lin, J. Xu, *Environ. Sci. Technol.* **2025**, *59*, 5382-5393.

[4] H. Meng, J. Zhou, C. Nie, W. Li, D. Li, Y. Zhang, Z. Ao, *J. Hazard. Mater.* **2025**, *492*, 138031.

[5] K. Yang, K. Li, Y. Yang, W. Li, S. Liu, *Chem. Eng. J.* **2025**, *512,* 162289.

[6] F. Wang, Y. Gao, H. Fu, S. S. Liu, Y. Wei, P. Wang, C. Zhao, J. F. Wang, C. C. Wang, *Appl. Catal. B Environ.* **2023**, *339*, 123178.

[7] T. Yang, S. Fan, Y. Li, Q. Zhou, *Chem. Eng. J.* **2021**, *419*,129590.

[8] Y. Gao, G. Wang, X. Wang, X. Dong, X. Zhang, *J. Hazard. Mater.* **2024**, *463*, 132888.

[9] Y. Zhang, Z. Zhang, *Chem Catal.* **2024**, *4*, 101056.

[10] Y. H. Li, C. Y. Chen, S. Gao, C. C. Wang, Y. Li, M. Liu, H. Chu, Z. Pan, S. Zhan, H. Ji, *Angew. Chem. Int. Ed.* **2025**, *64*, e202507772.

[11] L. Liu, A. Wang, J. Hu, H. Hou, S. Liang, J. Yang, *Chemosphere* **2023**, *331*, 138783.

[12] C. Hao, T. Li, Y. Xie, J. X. Zhou, F. Chang, L. Luo, Q. Liu, A. Abdukayum, G. Hu, *Adv. Funct. Mater.* **2024**, *35*, 2414036.

[13] Y. Pan, X. Zhang, T. Wu, B. Shao, T. Li, Q. He, Z. Chen, L. Zhou, S. Liu, X. Huang, Z. Liu, *Chem. Eng. J.* **2024**, *481*, 148506.

[14] Y. X. Huang, K. Y. Chen, S. X.Wang, S. Y. Zhao, L. Q. Yu, B. C, Huang, R. C. Jin, *Appl. Catal. B Environ.* **2024,** *341*, 123324.

[15] H. Z. Liu, X. X. Shu, M. Huang, B. B. Wu, J. J. Chen, X. S. Wang, H. L. Li, H. Q. Yu, *Nat. Commun.* **2024**, *15*, 2327.

[16] K. Zhu, W. Qin, Y. Gan, Y. Huang, Z. Jiang, Y. Chen, X. Li, K. Yan, *Chem. Eng. J.* **2023**, *470*, 144190.

[17] Z. Zhang, H. Ding, Y. Li, J. Yu, L. Ding, Y. Kong, J. Ma, *Sep. Purif. Technol.* **2022**, *283*, 120136.

[18] J. Lu, Y. Zhou, L. Ling, Y. Zhou, *Chem. Eng. J.* **2022**, *446*, 137067.

[19] X. Zhao, L. Duan, M. Chen, P. Yang, Q. Liu, Y. Liu, H. Zhang, Z. He, G. Hu, Y. Zhou, *Chem. Eng. J.* **2024**, *485*, 149928.

[20] J. Wang, J. Fan, J. Yao, X. Wu, C. Gao, Z. Wei, Y. Li, *Chem. Eng. J.* **2023**, *461*, 142024.

[21] J. Sun, D. Zhang, D. Xia, Q. Li, *Chem. Eng. J.* **2023**, *471*, 144832.

[22] M. Chen, T. Yang, Q. Lei, X. Gan, S. Mao, H. Zhao, *Angew. Chem. Int. Ed.* **2025**, *64*, e202416921.

[23] Q. Zhong, Y. Xue, Z. Qi, Y. Sun, L. Wu, D. Sun, C. Xu, K. Ri, S. Yang, J. Zhu, Q. Ji, Y. Liu, S. Li, H. He, *Appl. Catal. B Environ.* **2025**, *360*, 124539.

[24] Z. Yang, X. Xu, Z. Li, C. Liu, J. Jiang, S. Zhang, J. Miao, W. Liu, W. Liu, Z. Zou, Z. Li, *Angew. Chem. Int. Ed.* **2025**, *64*, e202503901.

[25] Z. Yang, X. Yang, G. An, D. Wang, *Appl. Catal. B Environ.* **2023**, *330*, 122618.

[26] Q. Tian, J. Chang, X. Peng, L. Geng, B. Gao, Q. Li, Y. Gao, X. Xu, *Angew. Chem. Int. Ed.* **2025**, *64*, e202503995.

[27] Z. Zhao, P. Wang, C. Song, T. Zhang, S. Zhan, Y. Li, *Angew. Chem. Int. Ed.* **2023**, *62*, e202216403.

[28] Z. Wu, B. Huang, X. Wang, C.-S. He, Y. Liu, Y. Du, W. Liu, Z. Xiong, B. Lai, *Environ. Sci. Technol.* **2023**, *57*, 14046-14057.

[29] P. Duan, J. Pan, W. Du, Q. Yue, B. Gao, X. Xu, *Appl. Catal. B Environ.* **2021**, *299*, 120714.

[30] F. Wang, Y. Gao, H. Fu, S. S. Liu, Y. Wei, P. Wang, C. Zhao, J. F. Wang, C. C. Wang, *Appl. Catal. B Environ.* **2023,** 339, 123178.

[31] L. Zhang, Y. Sun, R. Ge, W. Zhou, Z. Ao, J. Wang, *Appl. Catal. B Environ.* **2023**, *339*, 123130.

[32] K. Yin, Y. Shang, D. Chen, B. Gao, Q. Yue, X. Xu, *Appl. Catal. B Environ.* **2023**, *338*, 123029.

[33] X. Zhou, M.-K. Ke, G.-X. Huang, C. Chen, W. Chen, K. Liang, Y. Qu, J. Yang, Y. Wang, F. Li, H.-Q. Yu, Y. Wu, *Proc. Natl. Acad. Sci. USA* **2022**, *119*, e2313387121.

[34] J. Guo, Y. Wang, Y. Shang, X. Xu, *Proc. Natl. Acad. Sci. USA* **2024,** *121, e2313387121.*

[35] Z. Wu, Z. Xiong, B. Huang, G. Yao, S. Zhan, B. Lai, *Nat. Commun.* **2024**, *15*, 7775.

[36] J. Pan, B. Gao, P. Duan, K. Guo, M. Akram, X. Xu, Q. Yue, Y. Gao, *Mater. Chem. A* **2021**, *9*, 11604-11613.

[37] C. Cheng, W. Ren, F. Miao, X. Chen, X. Chen, H. Zhang, *Angew. Chem. Int. Ed.* **2023**, *62*, e202218510.

[38] Z. Wang, A. Xie, Z. Li, W. Chu, X. Lu, J. Zeng, H. Zhao, *Water Res.* **2025**, *268*, 122699.

[39] J. Zhen, J. Sun, X. Xu, Z. Wu, W. Song, Y. Ying, S. Liang, L. Miao, J. Cao, W. Lv, C. Song, Y. Yao, M. Xing, *Angew. Chem. Int. Ed.* **2024**, *63*, e202402669.

[40] H. Meng, J. Zhou, Y. Zhang, J. Cui, Y. Chen, W. Zhong, Y. Chen, C. Q. Jia, *Appl. Catal. B Environ.* **2025**, *366*, 125038.

[41] C. Liu, J. Li, X. He, J. Yue, M. Chen, J. P. Chen, *Proc. Natl. Acad. Sci. USA* **2024**, *121*, e2322283121.

[42] Y. Sun, J. Cao, Q. Li, D. Li, Z. Ao, *Mater. Chem. A* **2023**, *11*, 16586-16594.
